# Climate change/global warming/climate emergency versus general climate research: comparative bibliometric trends of publications

**DOI:** 10.1016/j.heliyon.2021.e08219

**Published:** 2021-10-19

**Authors:** Rafael M. Santos, Reza Bakhshoodeh

**Affiliations:** a School of Engineering, University of Guelph, Guelph, Ontario, N1G 2W1, Canada; b Department of Civil, Environmental and Mining Engineering, University of Western Australia, Perth, 6009, Australia

**Keywords:** Bibliometric analysis, Scientometrics, Human influence on climate, Natural control of climate, Improving climate monitoring, Climate variability, Climate models, CO_2_

## Abstract

This article presents and discusses the scientific publication record from 1910 to 2020 on two topics: "climate" (CL) and "climate change/global warming/climate emergency" (CC/GW/CE). The goal is to comparatively visualize how these two distinct publication records have evolved over time, from different classification perspectives, using publication ratios as the key indicator. It is found that research output related to the Earth's contemporary changing climate overtook that of general climate research in 2010, and the publication ratio (CC/GW/CE)/(CL) has been expanding in the last decade. There are significant differences in the publication countries and sources between the two topics. Differentiation factors that affect the level of research output and engagement on the climate challenge include island versus landlocked nations, specialized versus general scientific journals, academic versus institutional organizations. The future of the publication records is discussed, such as the emergence of new terms to refer to the climate challenge, such as “climate emergency”.

## 1 Introduction

The climate of a region is its average or typical weather over a long period of time; for example, the climate of Antarctica is freezing cold, and Hawaii is warm and sunny. Climate change, therefore, is a long-term change in the typical or average weather of a region; in the last few decades, industrial and human activities have led to gradually accelerating changes in the climate, including an annually incremental increase in the average surface temperature, which has been defined as climate change (IPCC, 2014). Climate change also has noticeable negative impacts on other parts of the planet, like changes in ecosystems and desertification, rise in sea level, flooding, and drought (Hisano et al., 2018; Ouhamdouch et al., 2019). The Intergovernmental Panel on Climate Change (IPCC) defines climate change as “a change in the state of the climate that can be identified… by changes in the mean and/or the variability of its properties and that persists for an extended period” (IPCC, 2018). Climate Change (CC), Global Warming (GW) and more recently Climate Emergency (CE) have been, in the past decade and longer, terms synonymous with the greatest sustainability challenge of the 21^st^ century (Munasinghe, 2010; Kyte, 2014; Princiotta and Loughlin, 2014; Martens et al., 2016).

Climate change mitigation is a technological measure aiming to reduce the amount of anthropogenic emissions of greenhouse gases (GHG) (Fawzy et al., 2020), and can be divided mainly into: (i) mitigation technologies, which focus on reducing fossil-based CO_2_ emissions, including nuclear power, renewable energies, and carbon capture and storage (Ricke et al., 2017; Bustreo et al., 2019); (ii) negative emissions technologies, which aim to capture and sequester atmospheric carbon to reduce carbon dioxide levels, and include approaches such as BECCS (bioenergy with carbon capture and storage), DACCS (direct air carbon capture and storage), enhanced rock weathering, and ocean fertilization (Goglio et al., 2020; Khalidy and Santos, 2021; Lezaun, 2021); and (iii) geoengineering techniques that change the Earth's radiative energy budget to stabilize or reduce global temperatures, such as stratospheric aerosol injection, and marine cloud brightening (Lockley et al., 2019; Osman et al., 2020). Controlling and reversing climate change is expected to be a major concern and undertaking for mankind in the forthcoming decades.

Bibliometric analysis is a popular technique commonly employed to investigate the internal relationships in the body of scientific outputs in the literature. This method is helpful for researchers who are interested in but unfamiliar with a specific field to understand the status of this field quickly. Various bibliometric studies have explored different topics related to climate change (Demiroglu and Hall, 2020), global warming (Marx et al., 2017), climate change's impact on human health, agriculture, and water resource management (Wang et al., 2014; Janssen et al., 2006; Li et al., 2011; Wei et al., 2015), and various scientific (Oliveira et al., 2020) and technological (Sobreira et al., 2020) questions. Studies that merge traditional bibliometrics with scientific topics also term these types of studies scientometrics (Janmaijaya et al., 2018; Sobreira et al., 2020). Such studies often rely on the most comprehensive literature databases available, including Web of Science and Scopus (Sobreira et al., 2020; Salmerón-Manzano and Manzano-Agugliaro, 2017; Macchi Silva et al., 2019). It is also common for such studies to span several decades (Janmaijaya et al., 2018; Oliveira et al., 2020), and cover regional (Marx et al., 2017; Demiroglu and Hall, 2020) to global (Salmerón-Manzano and Manzano-Agugliaro, 2017; Oliveira et al., 2020) topics.

In the last years, scientific publications and reports by scholars that study different aspects of climate change have rapidly increased (Aleixandre-Benavent et al., 2017). Aleixandre-Benevant et al. (2017) evaluated that the number of publications increase by over six-fold between 2005 and 2014. They used social networks to conclude that the United States is at the center of much of the research, and found relationships between keywords to find to which fields of research climate change research is primarily interconnected with (main keywords were: CO_2_, adaptation, model, temperature, and impact). According to the results of Fu and Waltman (2021), the number of publications on climate change topics in a country reflects the priorities set by its government to cover some of the existing issues. Geography and level of economic development were other factors associated with the scientific output of various countries or regions. Fu and Waltman (2021) also point to how the purpose of the research has been shifting in the last two decades from that concerned with the causes and effects of climate change to measures to reverse or incentivize the reversal of climate change. Due to the growing scientific and public attention to climate change, researchers have used the bibliometric method to characterize the intellectual landscape of climate change, including the impact of climate change on migration (Milán-García et al., 2021), tourism (Fang et al., 2018), and infectious diseases (Li et al., 2020).

An important aspect of bibliometric studies is the choice of search string used to retrieve publications from databases (Haunschild et al., 2016). Using too restrictive or specific keywords (e.g., simply “climate change”) can lead to an incomplete search record, so authors frequently use combinations and variations of keywords. For example: Aleixandre-Benevant et al. (2017) utilized [“climate change” OR “climate changes” OR “climatic change” OR “climatic changes”]; Fu and Waltman (2021) utilized [“climate chang∗” OR “climatic chang∗” OR “climate variabilit∗” OR “climatic variabilit∗” OR “global warming” OR “climate warming” OR “climatic warming”]; and Tan et al. (2021) utilized the largest combination among these three, [“climate change∗” OR “climatic change∗” OR “climatic variation” OR “climatic oscillation” OR “environmental risk∗” OR “environmental exposure” OR “environmental externalities” OR “ecological risk∗” OR “eco-risk∗” OR “climatic risk∗” OR “ecological management∗” OR “ecological governance” OR “ecological control” OR “environmental governance, environmental management∗” OR “environmental control” OR “environmental improvement” OR “eco-environmental risk∗” OR “low carbon” OR “carbon emission∗” OR “cost of emission reduction” OR “emission reducing potential” OR “emission reduction”]. Evidently, the search of Tan et al. (2021) would lead to inclusion of papers not related to climate change, such as those related to general climate research and those related to all forms of environmental impact and pollution. Fu and Waltman's search string also runs the risk of including general climate research via the term “climate variability”, but is the only one of the three to have included the term “global warming”. Haunschild et al. (2016) present a detailed discussed on how truncation and other operators can be used to narrowed down a bibliometric search to a specific area of research (climate change in their case), and also how additional keywords can then be used to split a large dataset into sub-sets based on specific sub-areas of the research field (e.g., the effects of climate change on ice and snow using the search terms [“∗ice∗” OR “∗glacier∗” OR “∗snow∗” OR “∗frost∗”] or on oceanic currents using the search terms [“∗el nino∗” OR “∗elnino∗” OR “∗southern oscillation∗” OR “∗enso∗” OR “∗Walker circulation∗” OR “∗north atlantic oscillation∗” OR “∗nao∗”]). Two things can be concluded from inspecting the various search strings used by authors of bibliometric studies: (i) it is critical to find a good balance between inclusion and exclusion of articles, and this is done by careful selection of search terms, focusing on the ones known to be frequently associated with the research topic, and by the use of the truncation (∗) operator; and (ii) there has not been a bibliometric study that has attempted to separate and analyze the unique research records related to climate change research from those related to general climate research. These are two important motivators on our present work.

In this article, we aim to comparatively explore the bibliometric and scientometric data on two topics: general “climate” research and “climate change/global warming/climate emergency” research. The former relates to research that builds on our understanding of what naturally governs the Earth's climate, and how the climate regulates natural processes on the Earth's surface; the latter relates to research that investigates what is causing the Earth's climate to change rapidly, primarily as a result of anthropogenic drivers, and what effects climate change has on the Earth's systems, and what could be done to mitigate or adapt to this. An inspiration we have used for this work is the historical importance of the work of British engineer Guy Callendar, who in 1938 pointed to the anthropogenic contribution to global temperature rise (Callendar, 1938), at a time before climate change research took off. That is, climate change research originated from general climate research, and at some point in the 20^th^ century, as will be presented later on in this article, became a unique field of research with a unique publication record.

Apart from the novel comparative topical theme, another differentiator of this article is its use of publication ratio values. We define the publication ratios as the number of publications in a category in one record over that in another record, which help us to distinguish and contrast CC/GW/CE versus general climate (CL) research. This approach differs from other comparative studies (e.g., Baek et al. (2020), Arana Barbier (2020), Wang et al. (2021)), in that the traditional approach for comparing records is to plot or tabulate the data of each record separately, and then compare the trends seen in each record. The publication ratio method allows more direct and precise comparisons, as are shown in this article. Yet another differentiator is that this article is hypothesis-driven; that is, hypotheses (presented below) are posed to guide the collection and analysis of the bibliometric and scientometric data. The testing of hypotheses allows for evaluation of the quality and effectiveness of the data analysis performed, and thus acts as a verification mechanism that often is lacking in traditional literature reviews. past studies on climate change do not attempt to isolate or exclude papers that relate to general climate research. To this end, we hereafter explore the publication trends of two records (CL and CC/GW/CE), since the topical terms appeared in the journal records in the early part of the 20^th^ century, to test the following hypotheses:

1. It is possible to substantially distinguish the scientific literature that pertains to the study of the aforementioned climate challenge (or solutions for mitigating it) from studies that address gaining a better understanding of the earth's climate itself, using topical keyword searches.
2. The scientific literature has become so enriched in works addressing the climate challenge that it surpassed climate research in terms of the number of publications sometime in the late part of the 20^th^ century.
3. The scientific literature that pertains to the climate challenge is at least partly distinct from that on climate research in terms of the venue of publication, country of origin of studies, and organizations that have conducted these works.

The present study is global in scope and covers a century of data, as it looks to highlight key moments in the publication record and scientific advancement histories, in addition to the temporal and various categorical trends. The following research questions have been formulated to contrast CL research versus CC/GW/CE research via hypothesis testing: (i) what are the dynamics of the conceptual structure of CC/GW/CE versus CL research; (ii) when the scientific record has become more enriched in CC/GW/CE versus CL research; (iii) in which countries the climate challenge has become the dominant topic and are there any relationships between countries and the dominant scientific topic?

## 2 Methodology

**Figure 1:**
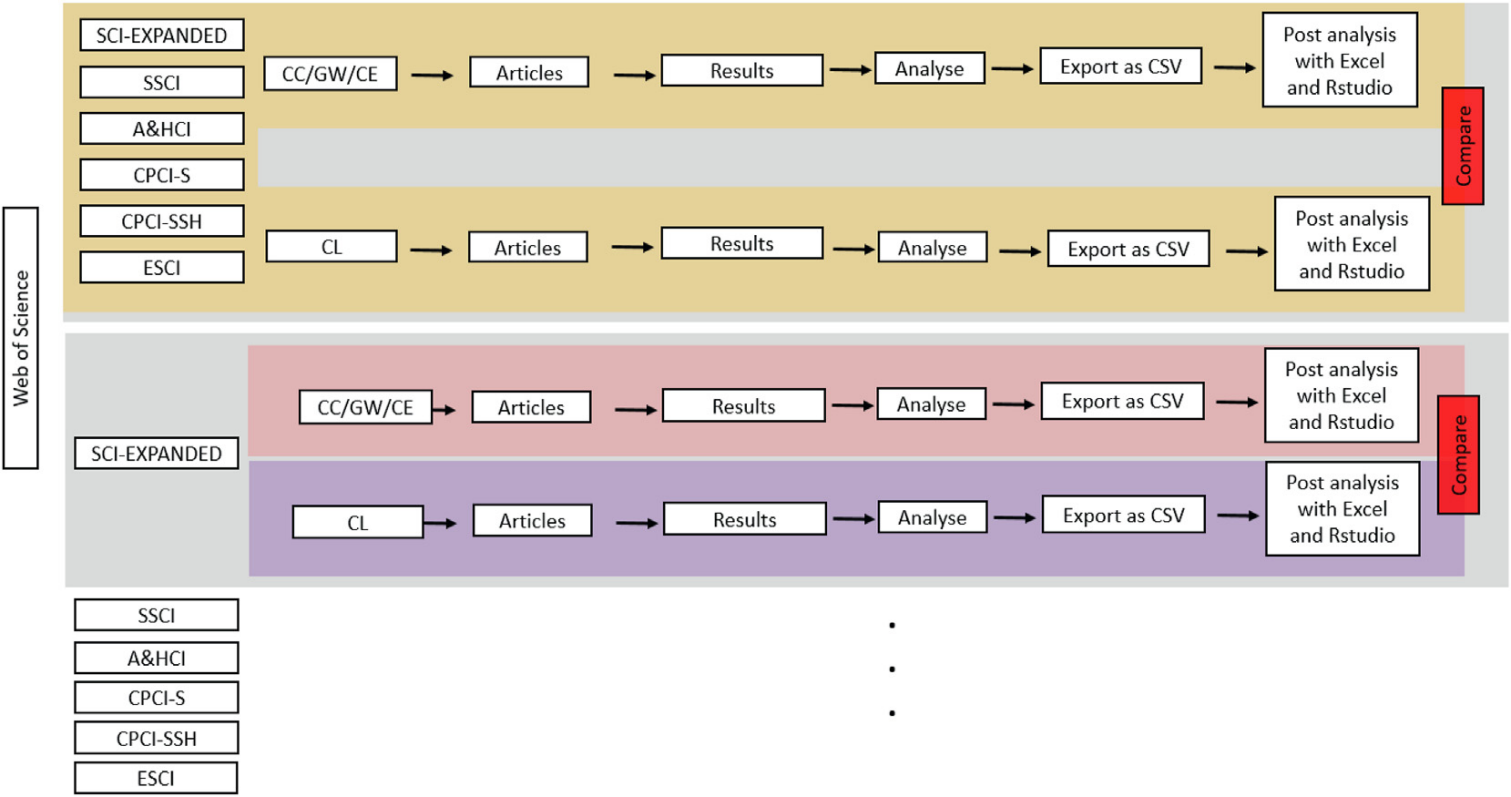
Protocol used for the bibliometric study.

Web of Science (WoS) was used to search the scientific literature and collect the relevant publication data for analysis. The searches were conducted on August 7^th^, 2021 (for 1900 to complete 2020 data); all data were collected within a short time on those days to obtain a snapshot of the publication record. Figure 1 shows the protocol used for this bibliometric study, which is classified into five steps detailed below.

Step one: The search used a time span of 1900–2020 and all indexes within the Web of Science Core Collection, namely: Science Citation Index Expanded: SCI-EXPANDED (1900–2020), Social Sciences Citation Index: SSCI (1900–2020), Arts & Humanities Citation Index: A&HCI (1975–2020), Conference Proceedings Citation Index - Science: CPCI–S (1990–2020), Conference Proceedings Citation Index - Social Science & Humanities: CPCI-SSH (1990–2020), and Emerging Sources Citation Index: ESCI (2015–2020). The two search strings used were: (i) TOPIC: ("climat∗ chang∗" OR "global warming∗" OR "climat∗ emergenc∗"); and (ii) TOPIC: ("climate" NOT ("climat∗ chang∗" OR "global warming∗" OR "climat∗ emergenc∗")). The former search string was used to collect papers related to research on the climate challenge (CC/GW/CE), and the latter search string was used to collect papers related to general research on the Earth's climate (CL). That is, these search strings tested Hypothesis 1.

Step two: In the results section, the document type was refined to ‘Article’. These searches yield 245,391 on the CC/GW/CE topic and 228,280 papers on the CL topic. The use of the NOR logical operator in the CL search string ensures that the two records are unique; that is, there are no repeating papers.

To verify if the search string used for CC/GW/CE research may have missed a substantial portion of research papers that did not use the three searched keywords, a third search was conducted using the following search string: TOPIC: ((("greenhouse gas∗" OR "GHG∗") NOT ("climate" OR "climate change" OR "global warming" OR "climate emergency"))). This search yielded 29,943 articles. This represents 11.56% of articles obtained with the combined search strings. In the Discussion and Conclusions section, the omission of these articles from the data analysis is explained.

Step three: The search results were at first analyzed using the Analyze Results feature of WoS. On the analysis page, it is possible to download tab-delimited text files containing a set of publication data according to the WoS category selected. Data files were obtained for the following four categories: publication years; organizations-enhanced; source titles; countries/regions. The data from these text files were then imported into Microsoft Excel for further processing and analysis. These data and analyses enabled testing Hypotheses 2 and 3. More details on the data handling procedure are provided in the Data Analysis section.

One additional procedure used was to recover keywords from the search records. This was done using the Export feature of WoS on the search results page to generate Excel files containing various attributes of each paper, including the keywords. Keywords were compiled from the top 100 cited papers from both topics (CC/GW/CE and CL) as of August 7^th^, 2021. These keywords were used to generate word clouds using the software Wordle (Feinberg, 2020).

Step four: A bibliometric analysis including keywords co-occurrence, countries collaboration, most relevant words, beamplots and affiliations was also performed on the full search results, which was exported from WoS as a bibtex or CSV file, using the bibliometrix package (Aria and Cuccurullo, 2017) in RStudio software Version 1.2.5001 (R Core Team, 2019). The bibliometrix R-package provides a set of tools for quantitative research in bibliometrics and scientometrics. It is written in the R language (Ihaka, 1998), which is an open-source environment and ecosystem (Aria and Cuccurullo, 2017). The codes used in this paper are provided at the end of the Supplementary Materials. Beamplot percentile data was obtained from author profiles found within Web of Science.

These data and analyses enabled testing Hypotheses 2 and 3, which are shown in the Supplementary Materials. This package uses the meta-data in the Web of Science citations to calculate and rank country production, journal sources, and country collaborations.

Step five: The previous steps (step one to four) were for all selected databases. In this step, each database was separately selected for analysis. Therefore, steps 2 to 4 were repeated again, and the results from each search were imported to Excel and Rstudio for further analysis.

## 3 Publication record highlights

From 1910 to 1970, the publications record for CC/GW/CE shows only eight entries, with 1971 being the first year with multiple (three) records. In fact, a line can be drawn at 1970 with the publication of Berton's paper titled “Carbon dioxide and its role in climate change” (Benton, 1970). This is the first of the papers on record to specifically address contemporary anthropogenic climate change. It does not mean that only in 1970 the role of humans on climate change was understood; such hypothesis dates to decades earlier. But it may be one of the first papers to consistently use the term “climate change” to describe the observed phenomena (increasing atmospheric concentration of greenhouse gases and increasing global surface temperatures, as discussed in the paper). Another evidence of this shift in terminology is that two of the three 1971 papers, those by Frisken (1971) and Kopec (1971), address the pressing climate challenge. This is in contrast with the first paper on this record, the 1910 Nature article by Lockyer titled “Does the Indian climate change?” (Lockyer, 1910), which discusses short term observations of changing frequency of monsoons to conclude that the climate on the sub-continent varies from year to year, with both short- and long-term trends, but the influence of humans on these trends is not addressed, in fact, the opposite (the natural control of climate) is inferred. Notably, both Frisken (1971) and Kopec (1971) highlight that by the early 1970's it is well accepted that humans can have a significant effect on the climate by air emissions, but that at that time (when CO_2_ atmospheric concentration have just surpassed 320 ppm (Benton, 1970)), the role of nature was still deemed stronger than the role of humans.

One way to contrast the publication record of CC/GW/CE versus that of CL is to compare their most cited works. Tables S-1 and S-2 in the Supplementary Materials also present the classification of 50 top cited papers for each record. Briefly reviewing these papers (i.e., reading the paper's aims and conclusions) helps understanding if the two data records contain the required topical selection of CC/GW/CE versus CL. Based on the results from these tables, for CC/GW/CE records, 45 papers are correctly classified, two are unusually misclassified, and three of them are outliers. While for CL records, 41 papers are correctly classified in CL subject, eight are misclassified, and one is an outlier. Within the top 5 most cited papers of each record, seven of the ten papers are correctly classified, two are unusually misclassified, and one is an outlier, as follows. Table 1 presents the publication history of the first authors of the five most cited journal articles from each record (according to Tables S-1 and S-2), in terms of the three most cited papers authored or co-authored by these first authors, on any topic (in some cases, the same topic as the record, and in some cases on other topics). Notable, the first most cited paper of each of these authors is also their highly cited paper according to Tables S-1 and S-2.

**Table 1:** The first three most cited journal articles of the five first authors of the highest cited papers from the two records (CC/GW/CE and CL), as tabulated in Tables S-1 and S-2.

**CC/GW/CE Record’s top 5 most cited**^1^**papers’ first authors**		**Top three most cited**^1^**papers of the first authors →**											
**First author name**	**Author’s first publication (year)**	**First highest cited paper (matches those in Table S-1)**				**Second highest cited paper**				**Third highest cited paper**			
		**Reference**	**Citations**	**Document Type**	**Subject**	**Reference**	**Citations**	**Document Type**	**Subject**	**Reference**	**Citations**	**Subject**	**Document Type**
Rayner, Nick	1985	(Rayner et al., 2003)	6,427	Article	CC	(Uppala et al., 2005)	5,610	Review	CL	(Reynolds et al., 2002)	3,208	CL	Article
Parmesan, Camille	1987	(Parmesan and Yohe, 2003)	6,216	Article	CC	(Walther et al., 2002)	6,071	Review	CC	(Parmesan, 2006)	4,964	CC	Review
Kottek, Markus	2005	(Kottek et al., 2006)	4,896	Article	CL	(Rubel and Kottek, 2010)	509	Article	CC	(Rubel and Kottek, 2011)	48	CL	Editorial Material
Thomas,Chris D.	1984	(Thomas et al., 2004)	4,324	Article	CC	(Chen et al., 2011)	2,375	Article	CC	(Biesmeijer et al., 2006)	1,643	CC	Article
Allen, Craig D.	1994	(Allen et al., 2010)	3,731	Article	CC	(McDowell et al., 2008)	2,153	Review	CC	(Breshears et al., 2005)	1,366	CC	Article
**CL Record’s top 5 most cited**^1^**papers’ first authors**		**Top three most cited**^1^**papers of the first authors →**											
**First author name**	**Author’s first publication (year)**	**First highest cited paper (matches those in Table S-2)**				**Second highest cited paper**				**Third highest cited paper**			
		**Reference**	**Citations**	**Document Type**	**Subject**	**Reference**	**Citations**	**Document Type**	**Subject**	**Reference**	**Citations**	**Subject**	**Document Type**
Kalnay, Eugenia	1976	(Kalnay et al., 1996)	21,389	Article	CL	(Kistler et al., 2001)	3,316	Article	CL	(Mesinger et al., 2006)	2,349	CL	Artile
Hijmans, Robert J.	1996	(Hijmans et al., 2005)	12,994	Article	CL	(Elith et al., 2006)	5,047	Article	CL	(Fick and Hijmans, 2017)	2,089	CL	Article
Taylor, Karl E	1976	(Taylor et al., 2012)	8,578	Article	CC	(Taylor, 2001)	3,495	Article	Out of scope	(Meehl et al., 2007)	2,059	CC	Article
Phillips, Steven J.	1969	(Phillips et al., 2006)	8,303	Article	CL	(Elith et al., 2006)	5,047	Article	CL	(Phillips and Dudik, 2008)	3,375	Out of scope	Article
Tenenbaum, Joshua B.	1991	(Tenenbaum et al., 2000)	7,281	Report	Out of scope	(Tenenbaum et al., 2011)	654	Review	Out of scope	(Steyvers and Tenenbaum, 2005)	620	Out of scope	Article

1 WoS Core citations as of August 7^th^ 2021.

Rayner et al. (2003) present sea ice and sea surface temperature and nighttime marine air temperature data sets, starting from 1871. That is, the study covers parameters pertinent for climate change research and the contemporary post-industrial revolution period attributed to anthropogenic climate change; after 18 years since their first publication in 1985, the first author reached their most cited paper in 2003. Parmesan and Yohe (2003) showed that climate change effects on living systems could be discerned from non-climatic effects by looking for systematic trends over diverse species and geographic regions; after 16 years since their first publication in 1987, the first author had reached their most cited paper in 2003. Kottek et al. (2006) provide a climate classification map update valid for the second half of the 20^th^ century, which was updated from the original 1961 Wladimir Köppen map. One motivation for this update was that climate changes have occurred and thus up-to-date global temperature and precipitation data sets were required to update the geographical distribution of the various climate zones (equatorial, arid, warm temperate, snow, and polar, and the various sub-classifications); one year after the first author's first publication on 2005, they reached their most cited paper in 2006. Thomas et al. (2004) showed how climate change leads to species-level extinction. They concluded that 18%–35% of species would be committed to extinction by 2050 because of climate change, in part because of habit loss due to changes in biome; after 20 years since the first author's first publication in 1984, they had reached their most cited paper in 2004. Allen et al. (2010) studied the effect of climate change and drought on trees mortality risks. They concluded that there is a direct relationship between tree mortality rates and heat severity and climate change; after 16 years since the first author's first publication in 1994, they had reached their most cited paper in 2010. All of these five highly cited papers are thus correctly classified under the CC/GW/CE topic. In addition, most (7 out of 10) of their second and third most cited papers are in the same research area (CC/GW/CE) as the record.

Kalnay et al. (1996) investigated how improvements to climate monitoring can avoid misinterpretation of climate variations that are not a result of climate change. The study is not concerned about studying climate change directly, even if the advances can benefit climate change research, and after 20 years since the first author's first publication in 1976, they had reached their most cited paper in 1996. Hijmans et al. (2005) developed a method for very high-resolution interpolation of temperature and precipitation climate data, which can be used to generate accurate climate surfaces (i.e., continuous grids); and after nine years since the first author's first publication in 1996, they had reached their most cited paper in 2005. This advance can help improve the analysis of climate change since more accurate values are obtained, though this was not the main aim of the study. An example was provided on how for Madagascar, the newly interpolated data set does not show direct evidence of climate change between 1930 and 1990. Another example stated that an insufficiently dense station network could lead to erroneous climate change conclusions. Phillips et al. (2006) present a model of the distribution of biological species due to geographic distribution, including climatic variables and conditions. The model was posed as being able to predict the movement of species due to climate change, such as invasive species, but this was not the study's main aim; after 37 years since the first author's first publication in 1969, they had reached their most cited paper in 2006. These are the three out of five highly cited papers correctly classified under CL. In addition, nearly all (5 out of 6) of their second, third most cited papers are also classified as CL research.

Tenenbaum et al. (2000) is the outlier. This paper does have relevance for CL research, as it pertains to the development of nonlinear algorithms to find trends in complex and large data sets, such as climate data sets, and is certainly not about CC/GW/CE. So while correctly classified, due to the use of the word “climate” in the abstract, the paper's topic is largely mathematical rather than about natural or engineering sciences. Taylor et al. (2012) is the paper that was unusually misclassified. This article does not have an abstract registered in WoS, and the article's single keyword registered in WoS is "climate" (the article itself does not have a keywords list). The article is in fact, about CC/GW/CE research; thus, the unusually incomplete record for this article caused it to be misclassified. These two papers highlight that the CL record is less robust than the CC/GW/CE record, particularly because of the CL record's less specific search string. While a weakness, the more analytical data processing presented in the Data Analysis section will show that this record is still useful for contrasting against the CC/GW/CE to yield dataset level (as opposed to paper-by-paper) trends and conclusions.

Table S-3 in the Supplementary Materials shows the top 5 cited paper in both records, which highlighted in blue (similar to the first column of Table 1) and 5 top papers in terms of citations which have cited these papers which are highlighted in grey. Almost all papers in each row are following the main papers’ topics (paper in the first column). For example, all articles that cited Hijmans et al. (2005) and Kalnay et al. (1996) were about climate modelling. Table S-4 in the Supplementary Materials lists the top 5 hot papers in both records which are highlighted in blue, and the top 5 hot papers in terms of citations that have cited the paper in the first column, which are highlighted in grey. According to WoS, hot papers are those that have been published in the last two years and have received enough citations to place them in the top 0.1% of papers in their academic fields. These papers demonstrate potential research hotspots and future research directions, providing readers with a more comprehensive understanding of these two studies. COVID-19 topics are one of the hottest topics due to the current situation and pandemic that most countries are dealing with; these papers cover the impact of COVID-19 on various aspects of our climate such as air pollution and microplastics. Furthermore, the majority of COVID-19-related papers are about climate change, which is classified in the CC/GW/CE record. Other hot topics in both records include air quality and wildlife conservation, such as insect extinction.

Bornmann and Marx first introduced beamplots in 2014 to better visualize the citation impact and productivity of researchers. In addition, beamplots are used to see performance variation over time to make more informed decisions about research impact and evaluation (Bornmann and Marx, 2014). The beamplot represents a single frame of an author's output (the citation performance of an author's entire publication list), which reflects how it varies over time. In the beamplot, each dot represents a specific publication and its position is based on its publication year and its normalized citation percentile score (0–100). For example, a score of 90 for an article means that the article is among the top 10% most cited publications of the subject area, document type, and year.

For the first authors of the top 3 most cited papers in each record, as listed in Table 1, the citation percentiles of their first authors were higher after publishing these articles, except in the case of Camile Parmesan, first author of Parmesan and Yohe (2003). Figure S-1 in the Supplementary Materials shows the beamplots of these six authors from both records. For example, in the case of N.A. Rayner, who has published the highest cited paper in the CC/GW/CE record, the mean citation percentile of their papers published after their highest cited paper (Rayner et al., 2003) has increased from 58% to 80%. Likewise but to a much lesser extent, for Eugenia Kalnay, who has published the highest cited paper in the CL record, the citation percentile was increased from 64.7% to 65.3%. This indicates that these authors either had more impactful research output following the publication of their most cited paper, or became better or more widely known after that date and hence received more citations to their latter work than their earlier work. The citation percentile of Camille Parmesan, who has the second highest cited paper (Parmesan and Yohe, 2003) in the CC/GW/CE record, was lower after 2003, when they published their highest cited paper, than before 2003. Figure S-1 shows that their citation percentiles during 2009 and 2010 were zero, which were for five book chapters, and causes this difference between the citation percentile before and after publishing their highest cited paper in 2003. Excluding these book chapters from the beamplot analysis leads to the conclusion that the author's performance actually improved after 2003. In summary, it is commonly the case that highly cited papers, whether they be on CC/GW/CE or CL topics, typically boost an author's citation profile.

**Figure 2:**
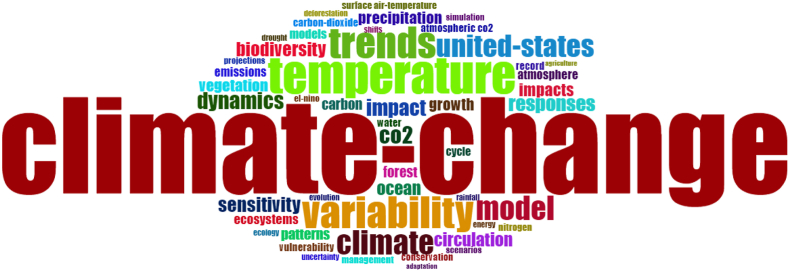
Word cloud of keywords from top 100 most cited papers on CC/GW/CE research.

**Figure 3:**
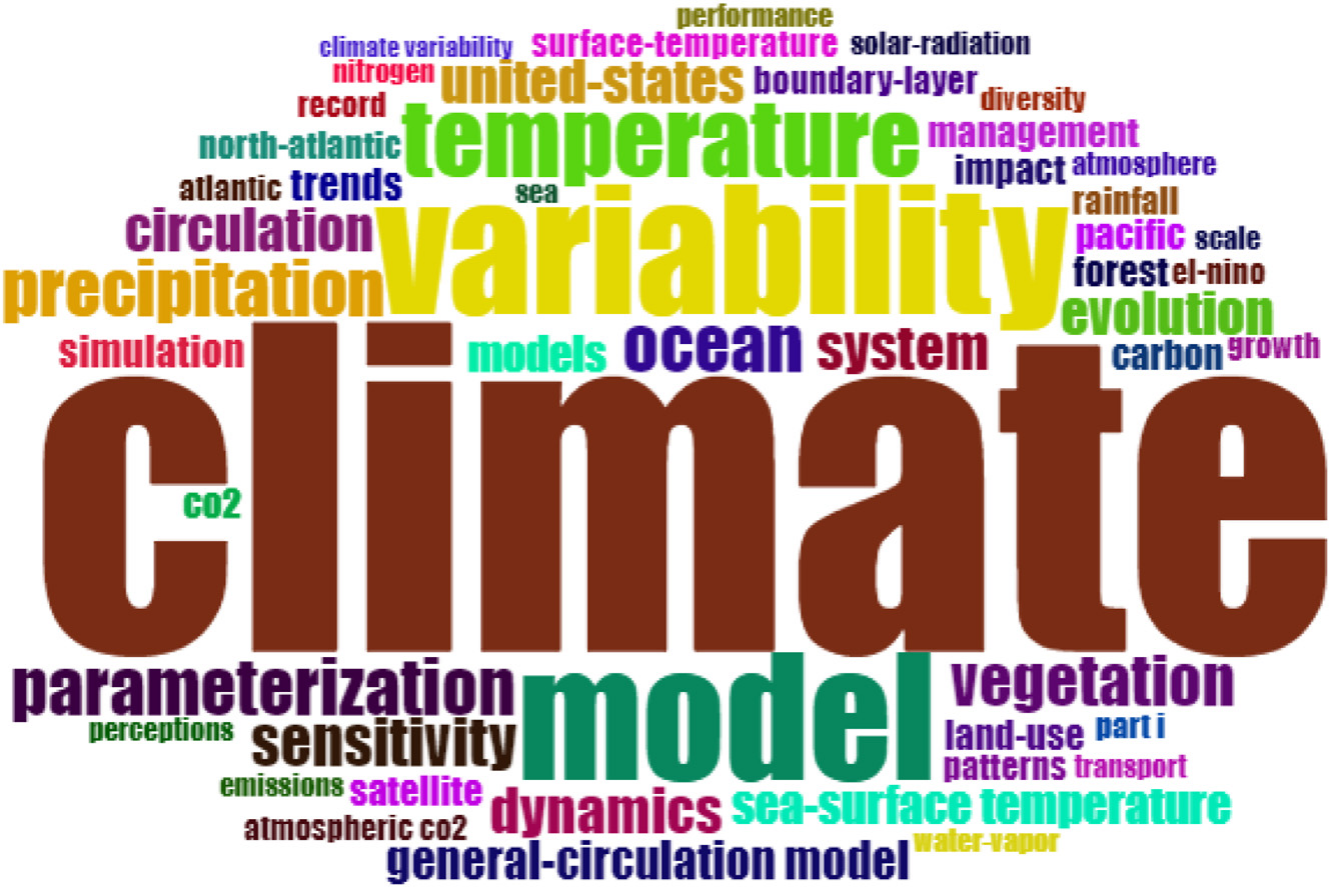
Word cloud of keywords from top 100 most cited papers on CL research.

Figures 2 and 3 present the word clouds generated for the keywords extracted from the top 100 most cited papers in each record. In contrast to the aforementioned analysis of the top 5 most cited papers in each record, which showed significant differences in the two records, the word clouds are qualitatively less precise. Table 2 also presents the top 10 words frequency for the top 100 cited papers in each record. It is understandable that research on CC/GW/CE will use many similar keywords to more general research on the Earth's climate, thus several terms are similarly enlarged on both clouds. For example, model, variability, temperature, precipitation and circulation are some of the main words on both clouds. In fact, the vast majority of words from Figure 2 also appear in Figure 3, even if in a different size. Climate change and CO_2_ are the two terms in Figure 2 that are particularly distinct from Figure 3, which is expected given that these are key topics of CC/GW/CE research. Figure S-2 in the Supplementary Materials also shows the word dynamic of both records over time. Based on the results from this figure, “climate change” and “climate” terms had the highest increase over time in terms of occurrence in articles. The conclusion from word clouds is that they are visually interesting, but are not ideal tools to evaluate two unique but topically similar publication records. As aforementioned, the Data Analysis section presents more deeply analytical comparisons between the two records, from which clearer trends can be seen.

**Table 2:** Word frequency of top ten used words in Figures 2 and 3.

CC/GW/CE record		CL record	
Word	Frequency ↓	Word	Frequency ↓
climate-change	140	climate	129
temperature	55	variability	51
variability	47	model	50
trends	46	temperature	31
climate	36	ocean	22
model	35	parameterization	22
united-states	33	precipitation	22
co2	26	vegetation	20
dynamics	25	sensitivity	19
impact	24	dynamics	18

Figure S-3 in the Supplementary Materials shows the co-occurrence analysis of keywords using the bibliometrix package in Rstudio in order to find research focus (Aria and Cuccurullo, 2017). Based on results from this figure, “climate change”, “climate”, and “variability” were the most frequent words among all keywords from 500 top most cited papers, which were similar to the key topics from the word clouds (Figures 2 and 3).

## 4 Data analysis

This section is sub-divided into the four categories of data collection and analysis of the publication records: (i) year of publication; (ii) country (corresponding author's) of publication; (iii) source (i.e., journal) of publication; and (iv) organization (corresponding author's) of publication.

### 4.1 Year of publication

**Figure 4:**
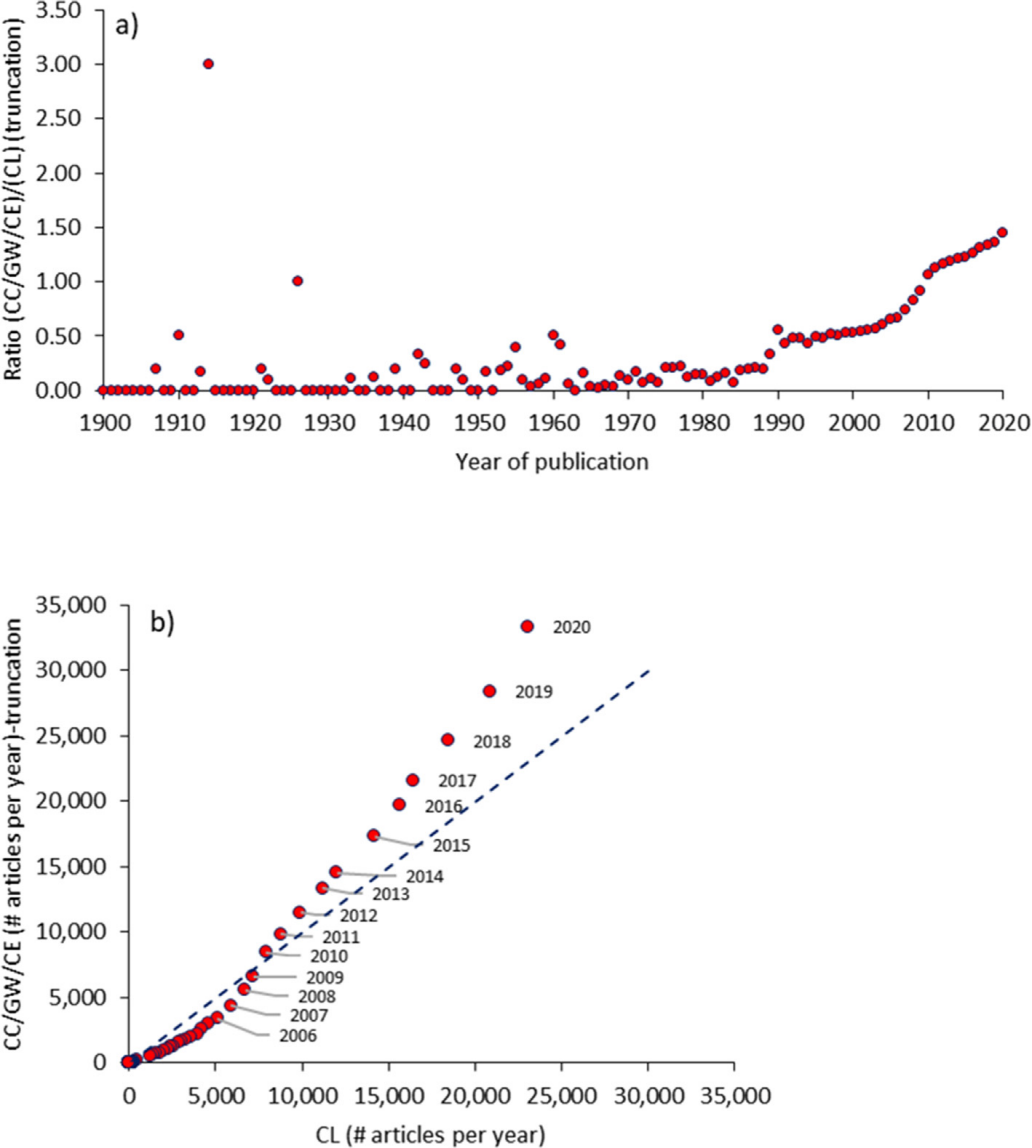
Data for CC/GW/CE and CL records for year of publication: a) Publication ratio ((CC/GW/CE)/(CL)) as a function of time (years); b) Number of publications per year in the CC/GW/CE record versus those in the CL record (dashed line illustrates the 1:1 mark), which are labelled with the year of each ratio for the period 2006 to 2020 (over this recent period, the annual CL publications continuously increased).

Figure 4 and Table S-5 in the Supplementary Materials present the data analysis for the year of publication, ranging from 1910 to 2020. The number of articles published per year in the two publication records (CC/GW/CE and CL) was compiled from WoS. For each year, a ratio of the number of articles in the CC/GW/CE record over the number of articles in the CL record was calculated. This ratio is plotted as a function of time in Figure 3a. The purpose of this ratio is to help visualize when the scientific record became more enriched in CC/GW/CE versus general CL research; that is when the ratio surpasses a value of one. This occurred in 2010, and the ratio has since increased to 1.36 in 2019 and then to 1.45 in 2020 (a full-year record). Notably, before 1989, the ratio was consistently smaller than 0.1, meaning that CC/GW/CE research was scarce for much of the 20^th^ century. The exceptions in 1910, 1939 and 1941 are due to the very small number of CL publications on record for those decades. From 1989 onwards, the ratio increases nearly every year (in fact, it increases 28 out of 31 times, and every year since 1997).

The number of publications in both records rose by orders of magnitude over the last several decades, and Figure 4b helps to visualize this climb. The CL record crossed 100 publications per year in 1975 versus 1990 for the CC/GW/CE record. The CL record crossed 1,000 publication per year also first, in 1991, followed by the CC/GW/CE record in 1996. Then both records breached 10,000 articles in a year in 2012. This coincides almost exactly with the 2010 threshold when the CC/GW/CE record overtook the CL in the number of publications per year. Points on Figure 4b above the dashed line indicate the records from the last decade, while those below the dashed line correspond to the pre-2010 record.

**Figure 5:**
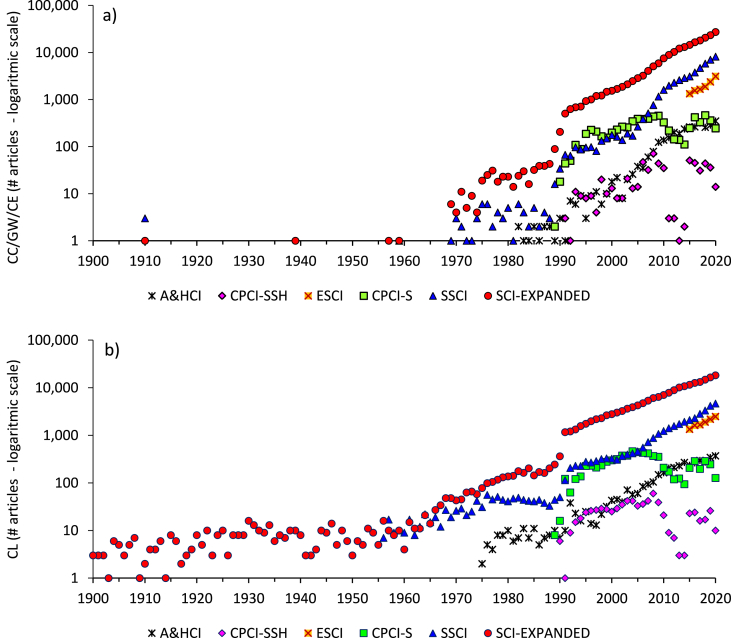
Data for the number of articles per year for all databases in logarithmic scale: a) CC/GW/CE and b) CL.

Figure 5 breaks down the two data records for the number of articles per year ranging from 1910 to 2020 according to the databases that make up the Web of Science Core Collection. The purpose of this analysis is to visualize if any unusual or sudden changes in the underlying databases could contribute to the trends observed in the full data sets. This could include the effect of databases entering the coverage of the Core Collection in a particular year, or the databases changing their coverage at some point in time. Figure 5 shows that the two largest and oldest databases, namely SCI-EXPANDED and SSCI (with coverage commencing in 1900), have similar temporal trends, to each other and to the Core Collection, given that they make up the most substantial portion of the latter. The ESCI is a newer database (started in 2015), and the A&HCI is a database with a focus on research areas far from the theme of climate research, hence the smaller size of its records in this analysis; their data set trends also are also in overall agreement. Figure 5 is plotted on a log-scale to magnify trends of the smaller data sets, and variability in the two CPCI data sets is evident for both records. Likely this variability is at least partly related with variable number of conference proceedings being indexed by Web of Science each year, and underlying changes in the types of venues used for publication of peer-reviewed papers. Notwithstanding, the small numbers of these data sets (in the order of tens to hundreds of papers per year in the last decades) have insignificant impact on the trends of the much larger overall Core Collection data sets. Tables S-6 and S-7 in the Supplementary Materials show the number of articles in the CL and CC/GW/CE records per year for all databases.

### 4.2 Country/region of publication

**Figure 6:**
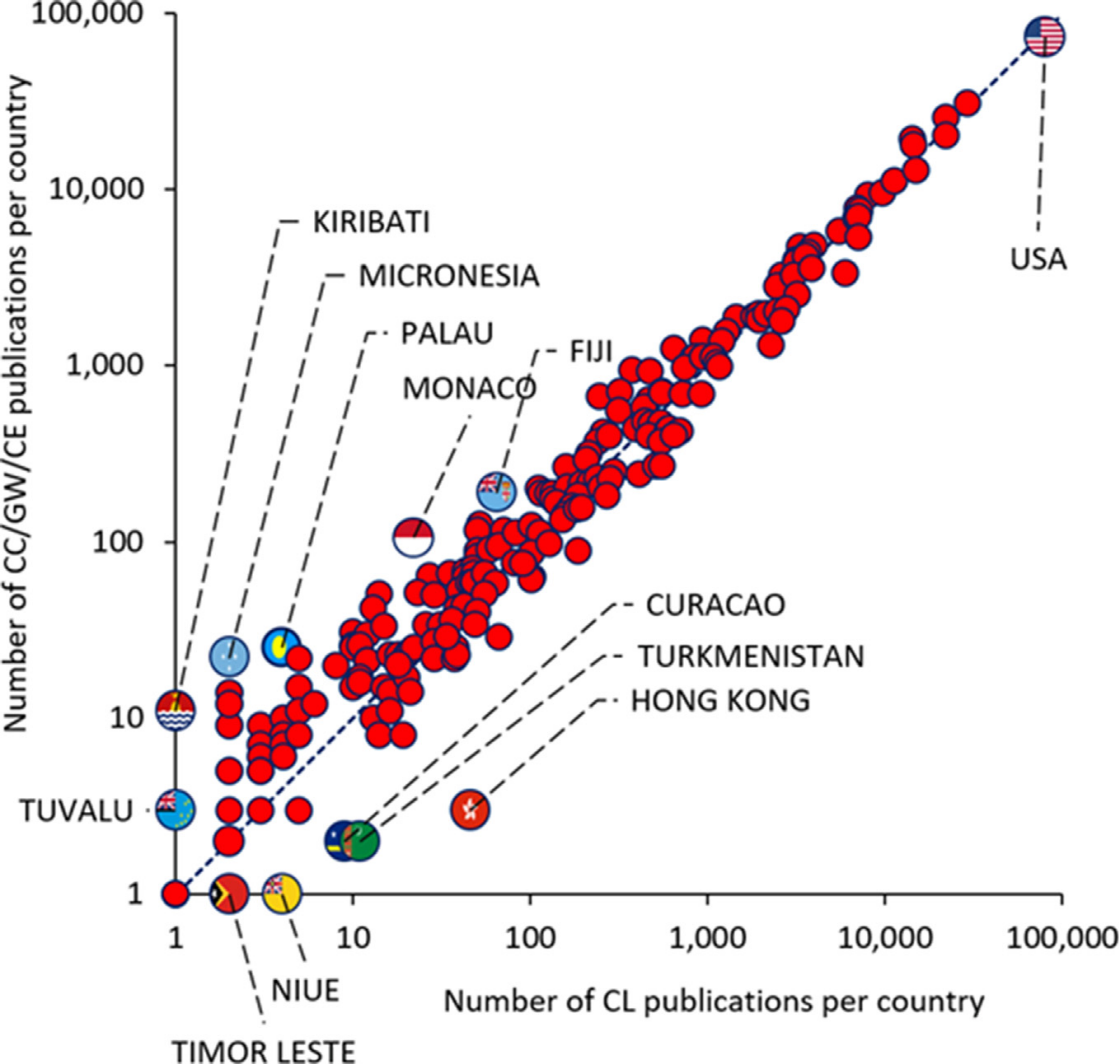
Data for CC/GW/CE and CL records for country/region of publication: Number of publications per country/region in the CC/GW/CE record versus those in the CL record (dashed line illustrates the 1:1 mark), which are labeled with the names of outlier countries.

Figure 6, Figures S-4 and S-5 in the Supplementary Materials, and Table S-8 in the Supplementary Materials present the data analysis for the country (or region) of publication for the full records ranging from 1910 to 2020. The number of articles published per country/region in the two publication records (CC/GW/CE and CL), from 1910 to 2020, was compiled from WoS. For each country/region, a ratio of the number of articles in the CC/GW/CE record over the number of articles in the CL record was calculated. This ratio is plotted for each country/region in Figure S-4, ordered from largest to smallest ratio. Countries/regions with a ratio greater than one have been more engaged in CC/GW/CE research, while those with a ratio smaller than one have been more engaged in CL research. The number of countries/regions for which a ratio was calculated is 210. An additional 32 countries of regions did not have a ratio calculated, either due to no CC/GW/CE or CL articles on record (this occurs for small states such as Equatorial Guinea and Turks and Caicos). In addition, countries that no longer exist and became part of other countries were merged with their successor countries which include the Soviet Union with Russia; Western Germany with Germany; Serbia Montenegro with Serbia; Yugoslavia with Serbia; Czechoslovakia with the Czech Republic; and Swaziland with Eswatini. The number of studies in these countries before and after merging is shown in Table S-7 in the Supplementary Materials. Figure S-4 shows that slightly more than half of the countries/regions have a ratio greater than one, indicating that the climate challenge has become a dominant scientific topic in many parts of the world. It is notable that the majority of countries/regions with ratios greater than 2 are island states, such as Philippines (ratio = 2.21), Fiji (ratio = 2.92), Bahamas (ratio = 3.23), Palau (ratio = 6.25), Micronesia (ratio = 11), and Kiribati (ratio = 11). This highlights that small island states are at most risk of the catastrophic effects of climate change, particularly rising sea levels (Vitousek et al., 2017; Horton et al., 2014; Nunn, 2009; King and Harrington, 2018; Widlansky et al., 2015).

In contrast, countries and regions with a ratio lower than 0.50 tend to be those of lower gross domestic product, those in arid regions of the world, or those landlocked nations, such as Albania, Djibouti, Algeria and Turkmenistan. Other countries of interest to view ratios for are those with long publication history (USA (0.91), England (1.17), France (0.86), Germany (0.92)) and the emerging/fast growing economies (China (1.07), India (1.00), Brazil (0.76), South Africa (1.27)). The average ratio of these eight countries is very close to 1 (0.99), showing that such countries contribute with diverse research. On a case-by-case basis, it may be possible to claim which countries are more engaged in the climate challenge, but this has to also take into account the fact that a rich amount of older literature from some countries, when CL research was dominant, may be holding back their ratio, but that it does not mean that currently, these countries are just as engaged as others in CC/GW/CE research.

Figure 6 provides a different view of the country/region publication records. By plotting the number of CC/GW/CE publications for each country/region versus the number of CL publications in the same country/region, it is possible to see a focusing effect about the 1:1 dashed line. Countries that have published more, have more diverse body of literature and tend towards the 1:1 line (the USA is the highest point). Countries that have published less are more likely to be more engaged in recent research and thus have more CC/GW/CE articles than CL articles. Notable outliers with more than 10 CL publications (i.e., farthest from the 1:1 line and with a robust body of literature) are Fiji (with the ratio of 2.92) and Monaco (with the ratio of 4.82), both above the line.

#### 4.2.1 Country collaboration

Figure S-6 shows the countries of collaboration of the first authors of the five most cited articles in the CC/GW/CE and CL records, based on the affiliations listed in these authors' articles. For the authors from the CC/GW/CE record, apart from the countries of affiliation of the authors (the USA, England and Austria), the most frequent countries of collaboration have been Germany, the USA, Australia, France and Spain. For the CL record, apart from the country of affiliation of the authors (all from the USA), the most frequent countries of collaboration have been England, Germany, Australia, Canada, China, and Japan. Notably, there is more variety in collaboration in the articles from the CL record. For example, Robert J. Hijmans has collaborated with Peru and the Philippines, which have serious problems with droughts and floods (Elith et al., 2006; Fick and Hijmans, 2017). Perhaps unsurprisingly, these highly cited authors collaborate most with anglophone and European countries far more than with others. With climate changing affecting every country on Earth, and disproportionally threatening populations in smaller and less wealthy countries, it would be great to see in the near future deeper engagement of lead researchers directly with the scientific communities in those countries. Among the top 500 articles in the CC/GW/CE record some of these engagements are happing, as seen in Figure S-8 in the Supplementary Materials. Moreover, according to Figures S-5, S-7 and S-8 in the Supplementary Materials, the countries' scientific production and collaboration for the CC/GW/CE record are significantly more diverse than that of the CL record. As mentioned earlier, small island states are at most risk of the catastrophic effects of climate change, particularly rising sea levels (Vitousek et al., 2017; Horton et al., 2014; Nunn, 2009; King and Harrington, 2018; Widlansky et al., 2015), which is reflected in the countries’ scientific production and collaboration maps for the CC/GW/CE record (Figures S-5 and S-8).

### 4.3 Source of publication

**Figure 7:**
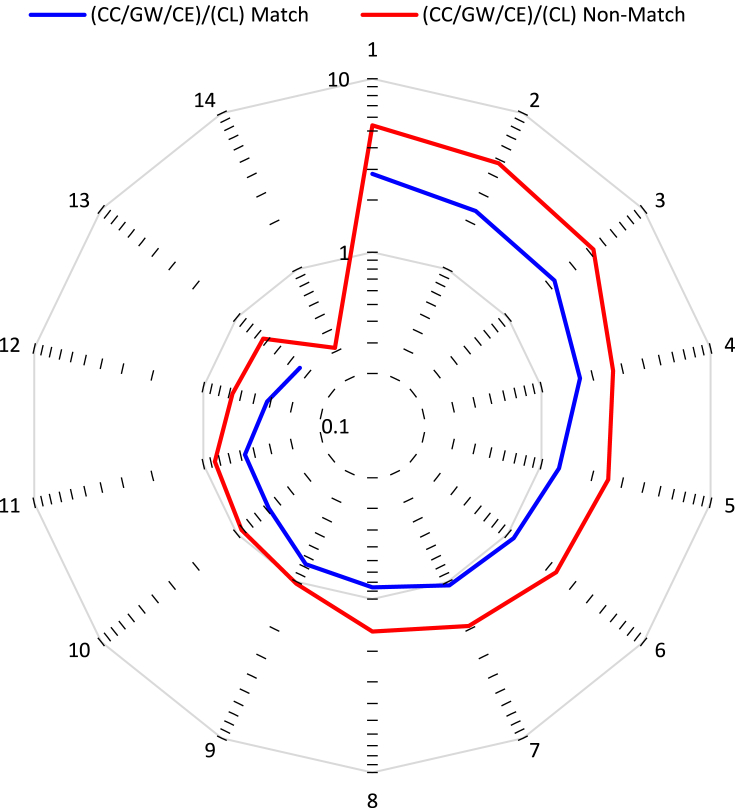
Publication ratio ((CC/GW/CE)/(CL)) for 27 journals, categorized as ‘match’ or ‘non-match’ based on appearance or not in both top 20 lists, ordered from highest to lowest ratio (one curve contains 13 entries and the other 14).

Table 3, Figure 7, present the data analysis for the source (i.e., journals indexed in WoS) of publication for a part of the records, ranging from 1910 to 2020. The sources analyzed are the top 20 venues of publications from each record in terms of the number of publications in each source. The top 20 were chosen to make the analysis manageable from a reporting and graphing perspective. The top 20 of the CC/GW/CE record represents 18.20% of all articles in this record, and the top 20 of the CL record represents a very similar 18.05% of that record. It is deemed that observations and trends made from the top 20 will be valid as a proxy for the trends of the full record.

**Table 3:** Top 20 sources of articles from the two records (CC/GW/CE and CL). The number of articles in each source, the percentage of the total number of articles in the full record, and the publication ratios ((CC/GW/CE)/(CL)). Bolded entries are the top 20 of each record, and bolded values reflect the entries that are top 20 on both records (i.e., “match”).

CC/GW/CE Sources ↓	Articles	% of 245,391	CL Sources ↓	Articles	% of 228,280	Ratio ((CC/GW/CE)/(CL))
Atmospheric Chemistry and Physics	761	0.310	**AtmosphericChemistry andPhysics**	2,420	1.060	0.31
Atmospheric Environment	638	0.260	**AtmosphericEnvironment**	1,001	0.438	0.64
**ClimateDynamics**	**1,809**	**0.737**	**ClimateDynamics**	**3,189**	**1.397**	**0.57**
**Climatic** C**hange**	3,458	1.409	Climatic Change	811	0.355	4.26
Earth and Planetary Science Letters	707	0.288	**Earth andPlanetaryScienceLetters**	1,053	0.461	0.67
**EnergyPolicy**	1,545	0.630	Energy Policy	623	0.273	2.48
**EnvironmentalResearchLetters**	1,901	0.775	Environmental Research Letters	839	0.368	2.27
Forest Ecology and Management	1,416	0.577	**ForestEcology andManagement**	921	0.403	1.54
**GeophysicalResearchLetters**	**2,805**	**1.143**	**GeophysicalResearchLetters**	**4,825**	**2.114**	**0.58**
**GlobalChangeBiology**	3,364	1.371	Global Change Biology	625	0.274	5.38
**InternationalJournal ofClimatology**	**1,777**	**0.724**	**InternationalJournal ofClimatology**	**2,309**	**1.011**	**0.77**
**Journal ofCleanerProduction**	2,323	0.947	Journal of Cleaner Production	487	0.213	4.77
**Journal ofClimate**	**2,212**	**0.901**	**Journal ofClimate**	**5,279**	**2.313**	**0.42**
**Journal ofGeophysicalResearchAtmospheres**	**1,786**	**0.728**	**Journal ofGeophysicalResearchAtmospheres**	**5,213**	**2.284**	**0.34**
**Journal ofHydrology**	**1,783**	**0.727**	**Journal ofHydrology**	**1,405**	**0.615**	**1.27**
**PalaeogeographyPalaeoclimatologyPalaeoecology**	**1,597**	**0.651**	**PalaeogeographyPalaeoclimatologyPalaeoecology**	**1,866**	**0.817**	**0.86**
**PLOSONE**	**3,602**	**1.468**	**PLOSONE**	**1,638**	**0.718**	**2.20**
**Proceedings of theNationalAcademy ofSciences of the United States of America(PNAS)**	1,625	0.662	Proceedings of the National Academy of Sciences of the United States of America (PNAS)	849	0.372	1.91
**QuaternaryInternational**	**1,421**	**0.579**	**QuaternaryInternational**	**1,352**	**0.592**	**1.05**
**QuaternaryScienceReviews**	**1,625**	**0.662**	**QuaternaryScienceReviews**	**1,478**	**0.647**	**1.10**
Remote Sensing	1,220	0.497	**RemoteSensing**	1,189	0.521	1.03
**Science of theTotalEnvironment**	**3,278**	**1.336**	**Science of theTotalEnvironment**	**1,387**	**0.608**	**2.36**
**ScientificReports**	**2,228**	**0.908**	**ScientificReports**	**1,319**	**0.578**	**1.69**
**Sustainability**	**2,663**	**1.085**	**Sustainability**	**942**	**0.413**	**2.83**
Theoretical and Applied Climatology	1,207	0.492	**Theoretical andAppliedClimatology**	1,412	0.619	0.85
**Water**	1,867	0.761	Water	704	0.308	2.65
Water Resources Research	923	0.376	**WaterResourcesResearch**	1,001	0.438	0.92

Table 3 presents the number of articles from each record that appear in these journals. A total of 27 journals appear in Table 3, organized in alphabetic order since there is a partial overlap of the top 20 from each record; in fact, there are 13 journals that are common to both top 20's (these are referred to as ‘match’ journals), and 14 journals in each top 20 that is not in the other top 20 (these are referred to as ‘non-match’ journals). The top journal in the CC/GW/CE record is PLOS ONE, with 3,602 articles representing 1.468% of the full record. The top journal in the CL record is the Journal of Climate, with 5,279 articles representing 2.284% of the full record. Both of these journals are in both top 20 lists, with PLOS ONE having a stronger record of CC/GW/CE articles as given by a ratio (as previously calculated to compare records) of 2.20, while the Journal of Climate has a stronger record of general CL research, with a 0.42 ratio. The ‘match’ journal with the highest ratio (2.36) is Science of the Total Environment, while the Journal of Geophysical Research Atmospheres has the lowest ratio of 0.34. The range of ratios is larger for ‘non-match’ journals. Here the highest ratio is 2.83 for the journal of Sustainability, and the smallest ratio is 0.31 for the journal of Atmospheric Chemistry and Physics. These ratios agree with the perception that can be taken about each of these journals. Journals like PLOS One, Science of the Total Environment and Global Change Biology appeal to more applied areas of research, including the applied sciences and engineering, and have wider aims and scopes, while journals such as the Journal of Climate, Journal of Geophysical Research Atmosphere and Atmospheric Chemistry and Physics appeal to more fundamental and specialized research.

Figure 7 helps to highlight the different scopes of the 27 journals listed in Table 3. Here, the ratios for each journal are plotted and categorized according to ‘match’ or ‘non-match’ journals. The spider plot shows data arranged from largest to smallest ratio for each category (i.e., each line). Had there been no significant difference in the distribution of the articles across the various journals, both of these lines would be very close to the value of 1. It is clear that the ‘non-match’ line deviates the most from 1, with nearly every value much higher or much lower than 1. Even the ‘match’ line deviates significantly from 1. The ratio value closest to 1 from both categories is that of the journal Remote Sensing (0.94). This is not surprising as remote sensing is a climate monitoring technique that can be used both for general climate research as well as to track changes in the climate due to anthropogenic effects (Kuenzer et al., 2011, Levizzani and Cattani, 2019; Milesi and Churkina, 2020). What can be concluded from this analysis is that journals and authors are able to distinguish the research topics sufficiently to steer more CC/GW/CE research to certain journals and more general CL research to other journals. This helps to confirm that these two topics are distinct in practice.

### 4.4 Organization of publication

**Figure 8:**
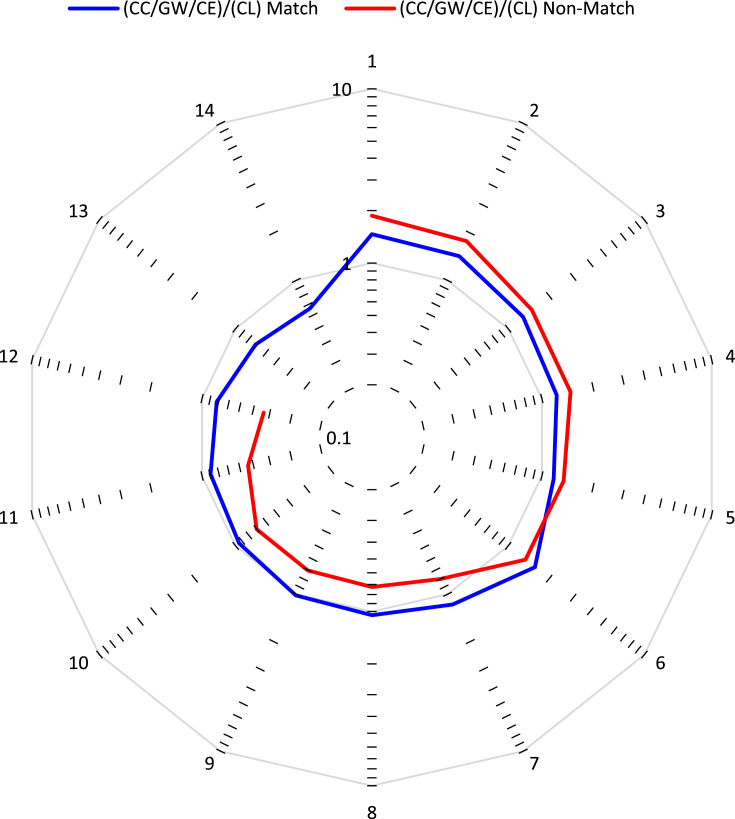
Publication ratio ((CC/GW/CE)/(CL)) for 26 organizations, categorized as ‘match’ or ‘non-match’ based on appearance or not in both top 20 lists, ordered from highest to lowest ratio (one curve contains 14 entries and the other 12).

Table 4, Figure 8, present the data analysis for organization (i.e., universities, research institutes, and other research-intensive organizations, associated with the corresponding author's primary affiliation) of publication, for a part of the records, ranging from 1910 to 2020. As with sources, the organizations analyzed are the top 20 from each record, and an identical data analysis procedure was used here. The top 20 of the CC/GW/CE record represents 36.72% of all articles in this record, and the top 20 of the CL record represents a slightly higher 38.53% of that record. A total of 26 organizations appear in Table 4, signifying that there are 14 common organizations within the top 20 (i.e., ‘match’ organizations), and 12 ‘non-match’ organizations. Here, the top three organizations are the same on both records, with the Chinese Academy of Sciences (China) being slightly more engaged in CC/GW/CE research than the the University of California System (USA) and the Centre National de la Recherche Scientifique (France); the ratios of these three organizations are very close to 1 (1.22, 1.05, and 1.02, respectively). In fact, the ratios of these organizations are much closer to 1, on average, than those of the journals. The highest ratio among the 26 organizations is 1.87 for the United States Forest Service, and the lowest ratio is 0.43 for the National Aeronautics Space Administration. This suggests that academic organizations may have more varied research, and hence ratios closer to 1, while governmental organizations may be more focused on a particular line of research, and thus rations more different than 1. Although such a conclusion would require analysis of a large set of organizations, and is complicated by some countries having organizations that have a dual academic and institutional role.

**Table 4:** Top 20 organizations of articles from the two records (CC/GW/CE and CL). The number of articles from each organization, the percentage of total number of articles in the full record, and the publication ratios ((CC/GW/CE)/(CL)). Bolded entries are the top 20 of each record, and bolded values reflect the entries that are top 20 on both records (i.e., “match”).

CC/GW/CE Organizations ↓	Articles	% of 245,391	CL Organizations ↓	Articles	% of 228,280	Ratio ((CC/GW/CE)/(CL))
**CentreNational de laRechercheScientifique(CNRS)**	**8,441**	**3.440**	**CentreNational de laRechercheScientifique(CNRS)**	**8,302**	**3.636**	**1.02**
**ChineseAcademy ofSciences**	**13,820**	**5.632**	**ChineseAcademy ofSciences**	**11,292**	**4.946**	**1.22**
Columbia University	2,358	0.961	**ColumbiaUniversity**	2,945	1.290	0.80
**CommonwealthScientificIndustrialResearchOrganisation(CSIRO)**	3,348	1.364	Commonwealth Scientific Industrial Research Organisation (CSIRO)	2,500	1.095	1.34
**ConsejoSuperior deInvestigacionesCientificas(CSIC)**	**3,524**	**1.436**	**ConsejoSuperior deInvestigacionesCientificas(CSIC)**	**2,742**	**1.201**	**1.29**
**HelmholtzAssociation**	**4,751**	**1.936**	**HelmholtzAssociation**	**5,327**	**2.333**	**0.89**
**Institut national de recherche pour l'agriculture, l'alimentation et l'environnement (INRAE)**	2,872	1.170	Institut national de recherche pour l'agriculture, l'alimentation et l'environnement (INRAE)	1,946	0.852	1.48
**Institut de recherche pour le developpement(IRD)**	**3,330**	**1.357**	**Institut de recherche pour le developpement(IRD)**	**2,835**	**1.242**	**1.17**
Max Planck Society	1,969	0.802	**Max Planck Society**	2,723	1.193	0.72
National Aeronautics Space Administration (NASA)	2,479	1.010	**NationalAeronauticsSpaceAdministration(NASA)**	5,713	2.502	0.43
National Center for Atmospheric Research (NCAR)	1,952	0.795	**NationalCenterforAtmosphericResearch(NCAR)**	3,643	1.596	0.54
**NationalOceanicandAtmosphericAdmininstration(NOAA)**	**3,566**	**1.453**	**NationalOceanicandAtmosphericAdministration(NOAA)**	**5,392**	**2.362**	**0.66**
**RussianAcademy ofSciences**	**2,740**	**1.117**	**RussianAcademy ofSciences**	**3,344**	**1.465**	**0.82**
**StateUniversitySystem of Florida**	**3,370**	**1.373**	**StateUniversitySystem ofFlorida**	**2,898**	**1.269**	**1.16**
**United StatesDepartment ofAgriculture(USDA)**	**4,408**	**1.796**	**United StatesDepartment ofAgriculture(USDA)**	**3,009**	**1.318**	**1.46**
**United StatesDepartment ofEnergy(DOE)**	**3,353**	**1.366**	**United StatesDepartment ofEnergy(DOE)**	**3,554**	**1.557**	**0.94**
**UnitedStatesDepartment of theInterior**	**4,234**	**1.725**	**United StatesDepartment of theInterior**	**2,694**	**1.180**	**1.57**
**United StatesForestService**	2,533	1.032	United States Forest Service	1,354	0.593	1.87
**United StatesGeologicalSurvey**	3,670	1.496	United States Geological Survey	2,462	1.078	1.49
**University of CaliforniaSystem**	**9,289**	**3.785**	**University of CaliforniaSystem**	**8,850**	**3.876**	**1.05**
**University of ChineseAcademy ofAciences**	**4,632**	**1.888**	**University of ChineseAcademy ofSciences**	**3,248**	**1.423**	**1.43**
University of Colorado Boulder	2,343	0.955	**University of Colorado Boulder**	3,339	1.462	0.70
**University of ColoradoSystem**	**2,513**	**1.024**	**University of ColoradoSystem**	**3,515**	**1.539**	**0.71**
**University of London**	3,100	1.263	University of London	2,307	1.010	1.34
University System of Maryland	1,850	0.754	**UniversitySystem of Maryland**	2,620	1.147	0.71
**WageningenUniversityResearch**	2,634	1.073	Wageningen University Research	1,482	0.649	1.78

Figure 8, in contrast to Figure 7, makes it clear that organizational information is not enough to place research as belonging to one record or another. Both the ‘match’ and ‘non-match’ lines deviated by small extents away from the ratio of 1 level, with the ‘non-match’ line deviating more, as would be expected. For comparison with the earlier case, one of the ratio values closest to 1 from both categories is that of the University of California System (1.05). Notable about this organization is that it consists of nine campuses offering comprehensive education, with varying levels of research excellence (e.g., excellent (Berkeley), very good (Davis), less highly ranked (Riverside), emerging (Merced) (Gibson et al., 2014). This can explain the diversity of research output, covering both CC/GW/CE and CL topics. Of course, this diversity of topic is an average since 1900, and it is possible that in recent years the research in many organizations has shifted towards the climate challenge, as the year and country trends presented earlier suggest.

## 5 Discussion

Here, we can revisit the three hypotheses and reach conclusions about their claims. With regards to hypothesis 1, it was possible to distinguish the scientific literature linked to CC/GW/CE research from that pertaining to CL research using the two search strings tested. This was verified by reviewing the scope of a number of highly cited papers in both records and is further supported by the trends seen with regards to years of publication, country/region of publication, and source of publication. That is, in these three categories, the publication records showed significant numerical and graphical differences, and these could at times be explained rationally, with basis on data interpretation. Evidence was found that the two publication records contained some misinterpreted publications and outliers, but it is deemed that the consistency of trends observed signify that these issues are minor and acceptable given the simplicity of the publication record assemblage method. A third search string, related to the topic of greenhouse gases, was also tested, but its data did not become part of this study's analysis. The reason for this is that such search string finds many articles that discuss the emissions of greenhouse gases (e.g., from flue gas stacks (Su et al., 2009) or from livestock farming (Herrero et al., 2013)) and technologies to control or mitigate these emissions (e.g., via carbon sequestration (Santos et al., 2013) or green energy (Panepinto et al., 2013)). As such, these articles fall outside the scope of the two topical records of interest here. This is further evidence that the two search strings used are effective in reaching their intended goal.

With hypothesis 1 confirmed, it is also possible to confirm hypothesis 2. The publication year data clearly shows that the scientific literature has become enriched in CC/GW/CE works in relation to CL works. This is despite both of these records experiencing massive growth over the decades (from under 100 articles per year in the first half of the 20^th^ century to over 10,000 articles per year in recent years). It is clear that the more pressing the climate challenge becomes, and its effects actually witnessed (Mahé et al., 2013), the more research is being undertaken to forecast the avoidable or unavoidable impacts (Ito et al., 2020; Döll et al., 2020). It is difficult to foresee for how much longer the publication ratio devised in this study will continue to rise, globally or country-by-country, as climate research will become increasingly important in the framework of a sustainable society, so it will be worth revisiting this in a decade or more. Perhaps by then another keyword could be added to complement CC/GW/CE. As of August 7^th^ 2021, 85.82% of this record is retrieved using only CC and excluding (via the NOT operator) the other two search terms (("climat∗ chang∗" NOT ("global warming∗" OR "climat∗ emergenc∗"))); this compares to only 8.32% of the record that only contains GW (("global warming∗" NOT ("climat∗ chang∗" OR "climat∗ emergenc∗"))), and a mere 0.0248% of the record that only contains CE (("climat∗ emergenc∗" NOT ("global warming∗" OR "climat∗ chang∗"))). A simple search for CE yields 61 articles, 53 of which published since 2019, and the oldest from 2011 (McMichael, 2011) being the most cited to date. This shows that this popular term (in the greater public sphere) is not yet commonly used scientifically; will it eventually be?

Hypothesis 3 was partly confirmed. The data and its interpretation show that the two publications records have distinct differences in terms of size (i.e., the number of publications) when it comes to the originating country/region and venue (journal) of publication. Yet, the two records are nearly indistinguishable when the criteria used are the organizations responsible for producing the work. As was explained, research organizations have broad research interests, and it is understandable that the same departments and research groups that perform CC/GW/CE research also tend to perform CL research. Of course, this would not be the case at the researcher level since expertise for these two topics of research is sufficiently different. WoS allows data analysis at the researcher (i.e., corresponding author level). However, in addition to the number of entries being very large (there are over 100,000 corresponding authors listed in the most recent CC/GW/CE and CL records), there is ambiguity with common author names (i.e., same last name and the same first letter of the first name), making any possible analysis less accurate. Such analysis would thus require close scrutiny at the article level.

## 6 Conclusions

This article presented and discussed the scientific publication record from 1910 to 2020 on two topics: "climate" and "climate change/global warming/climate emergency". The goal is to comparatively visualize how these two distinct publication records have evolved over time, from different classification perspectives, using publication ratios as the key indicator, which were presented as three hypotheses. To test our hypotheses, we defined publication ratios as the number of publications in a category in one record over that in another record, which allowed us to distinguish and contrast CC/GW/CE versus general CL research. The hypotheses can also be expressed as the following questions: (i) what are the dynamics of the conceptual structure of CC/GW/CE versus general CL research; (ii) when has the scientific record in CC/GW/CE versus general climate (CL) research become more enriched; (iii) which countries have made the climate challenge the dominant topic, and are there any links between countries and the dominant scientific topic? The following are the answers to these questions, which present the study's conclusions:

- The journal name and scope had a direct relationship with the number and ratio of publications in these two records; for example, journals like PLOS One, Science of the Total Environment, and Global Change Biology appealed to more applied areas of research, including the applied sciences and engineering, and have wider aims and scopes, while journals such as the Journal of Climate, Journal of Geophysical Research Atmosphere, and Journal of the Atmospheric Sciences appealed to more fundamental and specialized research.
- Governmental organizations focused more on a specific line of research (publication ratios farther from a value of 1), whereas academic organizations' research areas were broader and covered a wide range of topics (publication ratios closer to 1).
- It was discovered that research output related to the Earth's current changing climate surpassed that of general climate research in 2010, and the publication ratio (CC/GW/CE)/(CL) has been increasing over the last decade.
- Among other countries, island states such as the Philippines, Fiji, Bahamas, Palau, Micronesia, and Kiribati had the highest ratios, highlighting the fact that small island states are most vulnerable to the catastrophic effects of climate change, particularly rising sea levels.
- Ideas for future bibliometric studies that employ our hypothesis-driven approach and the use of publication ratios as the key trends indicator include: (i) inspecting more closely how non-scientific publications, such as those indexed by databases such as SSCI and A&HCI, have been evolving in covering the topics of climate change, global warming and the climate emergency; (ii) comparing the scientific literature that studies the causes and effects of climate change to the scientific literature that develops ways of mitigating or adapting to climate change or being resilient to it; (iii) identifying important topical gaps in the literature review record (e.g., well-cited articles or articles published in high impact journals that have not been covered in literature reviews); among other possibilities.

## Declarations

### Author contribution statement

Rafael M. Santos: Conceived and designed the experiments; Analyzed and interpreted the data; Wrote the paper.

Reza Bakhshoodeh: Performed the experiments; Analyzed and interpreted the data; Contributed reagents, materials, analysis tools or data; Wrote the paper.

### Funding statement

This research did not receive any specific grant from funding agencies in the public, commercial, or not-for-profit sectors.

### Data availability statement

Data will be made available on request.

### Declaration of interests statement

The authors declare no conflict of interest.

### Additional information

No additional information is available for this paper.

## Appendix A Supplementary data

Supplementary Materials-Santos and Bakhshoodeh Supplementary Materials-Santos and Bakhshoodeh
