# Supplementary material for: Climate change/global warming/climate emergency versus general climate research: comparative bibliometric trends of publications
Source: Heliyon. 2021 Oct 19;7(11):e08219. doi: 10.1016/j.heliyon.2021.e08219 (PMC8571708; doi:10.1016/j.heliyon.2021.e08219)
Supplement: Supplementary Materials-Santos and Bakhshoodeh [file mmc1.pdf]

Table S-1. Top 50 most cited articles classification in the CC/GW/CE record.

**CC/GW/CE record**								
**Reference**	**Year**	**Source**	**Country**	**Organization**	**Citations**	**Correctly classified**	**Incorrectly classified**	**Out of scope**
(Rayner et al., 2003a)	2003	J. Geophys. Res.-Atmos.	USA	Hadley Ctr Climate Predict & Res	6,114	x		
(Parmesan and Yohe, 2003)	2003	Nature	USA	Univ Texas	5,998	x		
(Kottek et al., 2006a)	2006	Meteorol. Z.	Austria	Univ Vet Med Vienna	4,413		x	
(Thomas et al., 2004b)	2004	Nature	England	Univ Leeds	4,184	x		
(Allen et al., 2010a)	2010	For. Ecol. Manage.	USA	US Geol Survey	3,474	x		
(Moss et al., 2010)	2010	Nature	USA	Univ Maryland	3,441	x		
(Lal, 2004)	2004	Science	USA	Ohio State Univ	3,164	x		
(Steffen et al., 2015)	2015	Science	Sweden	Stockholm Univ	2,996	x		
(Westerling et al., 2006)	2006	Science	USA	Univ Calif San Diego	2,865	x		
(Perez-Lombard et al., 2008)	2008	Energy Build.	Spain	Univ Seville	2,860	x		
(Root et al., 2003)	2003	Nature	USA	Stanford Univ	2,844	x		
(Guenther et al., 1995)	1995	J. Geophys. Res.-Atmos.	USA	Univ Colorado	2,778	x		
(Tilman et al., 2011)	2011	Proc. Natl. Acad. Sci. U. S. A.	USA	Univ Minnesota	2,658	x		
(Wang et al., 2016)	2016	Lancet	USA	Inst Hlth Metr & Evaluat	2,635	x		
(Charlson et al., 1992)	1992	Science	USA	Univ Washington	2,579	x		
(Adger, 2006)	2006	Glob. Environ. Change-Human Policy Dimens.	England	Univ E Anglia	2,552	x		
(Schmidt et al., 2011)	2011	Nature	Switzerland	Univ Zurich	2,521	x		
(Lloyd and Taylor, 1994)	1994	Funct. Ecol.	Australia	Australian National University	2,500	x		
(Held and Soden, 2006)	2006	J. Clim.	USA	Univ Miami	2,478	x		
(Vicente-Serrano et al., 2010)	2010	J. Clim.	USA	Inst Pirena Ecol	2,475	x		
(Cox et al., 2000)	2000	Nature	USA	Meteorol Off	2,466	x		
(Vorosmarty et al., 2000)	2000	Science	USA	Univ New Hampshire	2,456	x		
(Legates and McCabe, 1999)	1999	Water Resour. Res.	USA	Louisiana State Univ	2,421		x	
(Butchart et al., 2010)	2010	Science	USA	United Nations	2,393	x		
(Raich and Schlesinger, 1992)	1992	Tellus Ser. B-Chem. Phys. Meteorol.	USA	Iowa State Univ Sci & Technol	2,346	x		
(Fargione et al., 2008)	2008	Science	USA	Univ Minnesota	2,319	x		
(Smit and Wandel, 2006)	2006	Glob. Environ. Change-Human Policy Dimens.	Canada	Univ Guelph	2,290	x		
(Alexander et al., 2006)	2006	J. Geophys. Res.-Atmos.	England	Met Off	2,218	x		
(Meehl et al., 2007)	2007	Bull. Amer. Meteorol. Soc.	USA	Natl Ctr Atmospher Res	2,025	x		
(Emanuel, 2005)	2005	Nature	USA	Program Atmospheres Oceans & Climate	2,025	x		
(Dixon et al., 1994)	1994	Science	USA	Trexler & Associates Inc	2,015	x		
(Batjes, 2014)	2014	Eur. J. Soil Sci.	Netherlands	ISRIC	1,955	x		
(Batjes, 1996)	1996	Eur. J. Soil Sci.	Netherlands	ISRIC	1,955	x		

(Turner et al., 2003)	2003	Proc. Natl. Acad. Sci. U. S. A.	USA	Clark Univ	1,896	x		
(Jones et al., 2003)	2003	Eur. J. Agron.	USA	Univ Florida	1,883	x		
(Allouche et al., 2006)	2006	J. Appl. Ecol.	Israel	Hebrew Univ Jerusalem	1,866			x
(Lelieveld et al., 2015)	2015	Nature	Germany	Max Planck Inst Chem	1,863	x		
(Meinshausen et al., 2011)	2011	Clim. Change	Germany	Potsdam Inst Climate Impact Res PIK	1,854	x		
(Naghavi et al., 2017)	2017	Lancet	USA	Univ Washington	1,842	x		
(Webster et al., 2005)	2005	Science	USA	Georgia Inst Technol	1,834	x		
(Waycott et al., 2009)	2009	Proc. Natl. Acad. Sci. U. S. A.	Australia	James Cook Univ	1,827	x		
(Schar et al., 2004)	2004	Nature	Switzerland	Swiss Fed Inst	1,823	x		
(Dai, 2013)	2013	Nat. Clim. Chang.	USA	SUNY Albany	1,815	x		
(Deutsch et al., 2008b)	2008	Proc. Natl. Acad. Sci. U. S. A.	USA	Univ Washington	1,803	x		
(Collins et al., 2006)	2006	J. Clim.	USA	Natl Ctr Atmospher Res	1,773	x		
(Vos et al., 2017)	2017	Lancet	USA	Univ Washington	1,756	x		
(Trenberth and Hurrell, 1994)	1994	Clim. Dyn.	USA	National Ctr Atmospheric Res	1,754			x
(Menzel et al., 2006)	2006	Glob. Change Biol.	Germany	Tech Univ Munich	1,750	x		
(Perry et al., 2005)	2005	Science	England	Univ E Anglia	1,748	x		
(Laxminarayan et al., 2013)	2013	Lancet Infect. Dis.	USA	Ctr Dis Dynam Econ & Policy	1,733			x

Table S-2. Top 50 most cited articles classification in the CL record.

CL record								
**Reference**	**Year**	**Source**	**Country**	**Organization**	**Citations**	**Correctly classified**	**Incorrectly classified**	**Out of scope**
(Kalnay et al., 1996)	1996	B Am Meteorol Soc	USA	Natl Ctr Environm Predict	20,771	X		
(Hijmans et al., 2005)	2005	Int J Climatol	USA	Univ Calif Berkeley	12,438	X		
(Taylor et al., 2012)	2012	B Am Meteorol Soc	USA	Lawrence Livermore Natl Lab	7,962		x	
(Phillips et al., 2006)	2006	Ecol Model	USA	AT&T Labs Res	7,745	X		
(Tenenbaum et al., 2000a)	2000	Science	USA	Stanford Univ	7,015			x
(Zachos et al., 2001)	2001	Science	USA	Univ Calif Santa Cruz	6,120	x		
(Foley et al., 2005)	2005	Science	USA	Univ Wisconsin	6,030		x	
(Uppala et al., 2005b)	2005	Q J Roy Meteor Soc	England	European Ctr Medium Range Weather Forecasts	5,524	x		
(Hurrell, 1995)	1995	Science	USA	Nat Ctr Atmospheric Res	5,506	x		
(Rockstrom et al., 2009)	2009	Nature	Sweden	Stockholm Univ	5,401	x		
(Peel et al., 2007a)	2007	Hydrol Earth Syst Sc	Australia	Univ Melbourne	5,259	x		
(Mantua et al., 1997)	1997	B Am Meteorol Soc	USA	Univ Washington	4,650	x		
(Mlawer et al., 1997)	1997	J Geophys Res-Atmos	USA	Atmospher & Environm Res Inc	4,361	x		
(Hooper et al., 2005)	2005	Ecol Monogr	USA	Western Washington Univ	4,298	x		
(Wright et al., 2004)	2004	Nature	Australia	Macquarie Univ	4,221	x		
(Hansen et al., 2013)	2013	Science	USA	Univ Maryland	4,010		x	
(Scheffer et al., 2001)	2001	Nature	USA	Wageningen Univ	3,959	x		
(Arnold et al., 1998)	1998	J Am Water Resour As	USA	ARS, USDA, Temple	3,930	x		
(Petit et al., 1999)	1999	Nature	USA	Lab Glaciol & Geophys Environm	3,684	x		
(Harris et al., 2014)	2014	Int J Climatol	England	Univ E Anglia	3,537	x		
(Hong et al., 2006)	2006	Mon Weather Rev	USA	Yonsei Univ	3,303	x		
(Chen and Dudhia, 2001a)	2001	Mon Weather Rev	USA	Natl Ctr Atmospher Res	3,261	x		
(Xie and Arkin, 1997)	1997	B Am Meteorol Soc	USA	Natl Weather Serv	3,219	x		
(Foley et al., 2011)	2011	Nature	USA	Univ Minnesota	3,185	x		
(Galloway et al., 2008)	2008	Science	USA	Univ Colorado	3,098	x		
(Reynolds et al., 2002)	2002	J Climate	USA	Natl Ctr Environm Predict	3,095	x		
(Rienecker et al., 2011)	2011	J Climate	USA	Nasa, Global Modeling & Assimilat Off	3,080	x		
(Dansgaard et al., 1993)	1993	Nature	Iceland	Univ Iceland	3,066	x		
(Saha et al., 2010)	2010	B Am Meteorol Soc	USA	Ncep Nws Noaa	3,023	x		
(Mitchell and Jones, 2005)	2005	Int J Climatol	England	Univ E Anglia	2,947	x		
(Saji et al., 1999)	1999	Nature	Japan	Inst Global Change Res	2,869	x		
(Charlson et al., 1987)	1987	Nature	USA	Coombe Mill Exptl Stn	2,856	x		
(Grimm et al., 2008)	2008	Science	USA	Arizona State Univ,	2,821	x		
(Alvares et al., 2013b)	2013	Meteorol Z	Brazil	Forestry Sci & Res Inst Ipef	2,809	x		

(Large et al., 1994)	1994	Rev Geophys	USA	Natl Ctr Atmospher Res	2,677		x	
(Stuart et al., 2004)	2004	Science	USA	Iucn Species Survival Commiss Conservat	2,657		x	
(Bond et al., 2013)	2013	J Geophys Res-Atmos	USA	Univ Illinois	2,603		x	
(Jobbagy and Jackson, 2000)	2000	Ecol Appl	USA	Duke Univ	2,571	x		
(Berger and Loutre, 1991)	1991	Quaternary Sci Rev	Belgium	Inst Astron & Geophys G Lemaitre	2,483	x		
(Ramanathan et al., 2001)	2001	Science	USA	Univ Calif	2,428	x		
(Gorham, 1991)	1991	Ecol Appl	USA	Univ Minnesota	2,410	x		
(McGill et al., 2006)	2006	Trends Ecol Evol	Canada	Mcgill Univ	2,300		x	
(Smith et al., 2008a)	2008	J Climate	USA	Univ Maryland	2,295	x		
(Amabile et al., 1996)	1996	Acad Manage J	USA	Colgate Univ	2,292	x		
(Barnett et al., 2005)	2005	Nature	USA	Univ Calif	2,291	x		
(Ravishankara et al., 2009)	2009	Science	USA	Noaa, Div Chem Sci	2,251	x		
(Milliman and Syvitski, 1992)	1992	J Geol	USA	Woods Hole Oceanog Inst	2,251	x		
(Mesinger et al., 2006)	2006	B Am Meteorol Soc	USA	Ncep Environm Modeling Ctr	2,250	x		
(Chen et al., 2011b)	2011	Science	USA	Univ York	2,207		x	
(Monteith, 1977)	1977	Philos T Roy Soc B	England	Univ Nottingham	2,204	x		

Table S-3. Scientometric analysis of research hotspots: the 5 most cited papers in each record (CC/GW/CE and CL) shown in the first column, and in the remaining columns the top 5 papers in terms of citations that have cited each of the top cited papers in the first column.

**Top cited papers ↓**	**Top 5 papers in term of citations which have cited the paper in the first column →**														
	**First paper**	**Citation**	**Research hot spots**	**2nd paper**	**Citation**	**Research hot spots**	**3rd paper**	**Citation**	**Research hot spots**	**4th paper**	**Citation**	**Research hot spots**	**5th paper**	**Citation**	**Research hot spots**
**CC/GW/CE Record**															
(Rayner et al., 2003b)	(Uppala et al., 2005a)	5,610	Numerical weather prediction	(Reynolds et al., 2007)	2,370	Sea surface temperature	(Smith et al., 2008b)	2,352	Land-ocean surface temperature	(Compo et al., 2011)	2,235	Sea-level pressure	(Emanuel, 2005)	2,096	Hurricane intensity
(Parmesan and Yohe, 2003)	(Parmesan, 2006)	4,964	Ecological change	(Thomas et al., 2004a)	4,327	Extinction risk from climate change	(Chen et al., 2011a)	2,375	Extinction risk	(Chen et al., 2011a)	2,061	Soil moisture-climate interactions	(Deutsch et al., 2008a)	1,912	Impact of change on terrestrial organisms
(Kottek et al., 2006b)	(Peel et al., 2007b)	5,723	Climate classification	(Alvares et al., 2013a)	3,357	Climate classification	(Beck et al., 2018)	657	Climate classification	(Wright and Wimberly, 2013)	517	Land use change	(Rubel and Kottek, 2010)	509	Climate classification
(Thomas et al., 2004a)	(Srivastava et al., 2021)	102	Vegetation diversity	(Xia et al., 2021)	59	Structural concrete	(Thurner et al., 2021)	56	Rare species extinction	(Wan et al., 2021)	44	Photocatalytic reduction of CO_2	(Kechnebbou et al., 2021)	-	Impact of climate change on reptiles
(Allen et al., 2010b)	(Collins et al., 2013)	1,572	Asian summer monsoon	(Zhao and Running, 2010)	1,489	Terrestrial net primary production	(Choat et al., 2012)	1,221	Vulnerability of forests to drought	(Williams et al., 2013)	981	Forest drought stress and tree mortality	(Allen et al., 2015)	941	Tree mortality and forest die-off
**CL Record**															
(Kalnay et al., 1996)	(Dee et al., 2011)	15,958	Forecast model	(Uppala et al., 2005a)	5,610	Numerical weather prediction	(Kanamitsu et al., 2002)	4,099	Modelling (land–ocean fluxes)	(Chen and Dudhia, 2001b)	3,441	Numerical weather prediction	(Chen and Dudhia, 2011)	3,316	Numerical weather prediction
(Hijmans et al., 2005)	(Elith* et al., 2006)	5,048	Distribution models	(Alvares et al., 2013a)	3,357	Climate classification	(Alvares et al., 2013a)	2,111	Climate surface	(Pearson et al., 2007)	1,700	Species distribution modelling	(Daly et al., 2008)	1,576	Physiographically sensitive mapping of climatological temperature
(Taylor et al., 2012)	(Collins et al., 2013)	1,572	Asian summer monsoon	(Eyring et al., 2016)	1,356	Climate change modelling	(Hurrell et al., 2013)	1,259	Atmosphere model	(Ciais et al., 2014)	1,252	Carbon and Other Biogeochemical Cycles	(Jacob et al., 2014)	1,082	Regional climate change
(Phillips et al., 2006)	(Elith* et al., 2006)	5,048	Distribution models	(Phillips and Dudík, 2008)	3,375	Species distributions	(Elith and Leathwick, 2009)	3,149	Species distribution models	(Elith et al., 2011)	2,991	Species distribution model	(Pearson et al., 2007)	1,700	Species distribution modelling
(Tenenbaum et al., 2000b)	(Van der Maaten and Hinton, 2008)	9,948	Computer science/ dimensionality reduction	(Hinton and Salakhutdinov, 2006)	8,166	Computer science/ Reducing the Dimensionality of Data	(Bengio et al., 2013)	4,007	Data science/ deep learning	(Belkin and Niyogi, 2003)	3,986	Data science/ data representation	(Goodfellow et al., 2016)	3,683	Data science/ Deep learning

Table S-4. Scientometric analysis of research hotspots: the top 5 most cited "hot papers" (as classified by Web of Science) in each record (CC/GW/CE and CL) in the first column, and in the remaining columns the top 5 papers in terms of citations that have cited each of the hot papers in the first column.

Top "hot papers" ↓	Citation	Research hot spots	Top 5 hot papers in term of citations which have cited the paper in the first column →														
			**First paper**	**Citation**	**Research hot spots**	**2nd paper**	**Citation**	**Research hot spots**	**3rd paper**	**Citation**	**Research hot spots**	**4th paper**	**Citation**	**Research hot spots**	**5th paper**	**Citation**	**Research hot spots**
**CC/GW/CE Record**																	
**(Hersbach et al., 2020)**	1,065	Climate reanalysis	(Kelley et al., 2020)	44	General circulation model	(Petetin et al., 2020)	31	COVID-19 and pollution	(Ordonez et al., 2020)	23	COVID-19 and pollution	(von Schuckmann et al., 2020)	22	Sea-level rise	(Harrigan et al., 2020)	20	Global river discharge
**(Van Bavel et al., 2020)**	919	COVID-19 pandemic response	(Sibley et al., 2020)	134	Covid-19 and trust, attitudes toward government	(West et al., 2020)	133	Behaviour change	(Elmer et al., 2020)	120	COVID-19 and Comparisons of students' social networks	(Losada-Baltar et al., 2021)	102	COVID-19 and depression	(Alyami et al.)	81	Fear of COVID-19 Scale
**(Alyami et al.)**	448	Land-cover change	(Le Quere et al., 2020)	430	Covid-19 and pollution	(McDowell et al., 2020)	92	Pervasive shifts in forest dynamics	(Boucher et al., 2020)	85	Climate response to natural and anthropogenic forcings	(Forster et al., 2020)	73	Global climate and COVID-19	(Forster et al., 2020)	72	COVID-19 and pollution
**(Reid et al., 2019)**	407	Freshwater biodiversity	(Cardoso et al., 2020)	104	Insect extinctions	(Fadare and Okoffo, 2020)	94	Covid-19 face masks: A potential source of microplastic	(Weigand et al., 2019)	83	Biological monitoring by using short DNA	(Tickner et al., 2020)	77	Global freshwater biodiversity loss	(Aragaw, 2020)	55	Covid-19 face masks: A potential source of microplastic
**(Bastin et al., 2019)**	364	The global tree restoration potential	(Diaz et al., 2019)	247	The human impact on life on earth	(Liao et al., 2020)	130	Low-carbon footprint chemicals production	(Piao et al., 2020)	130	Global greening	(Chazdon and Brancalion, 2019)	68	Restoring forests	(Veldman et al., 2019)	64	The global tree restoration potential
**CL records**																	
**(Gossling et al., 2020)**	545	Pandemics, tourism and global change	(Sigala, 2020)	159	Tourism and covid-19	(Hall et al., 2020)	143	Pandemics, transformations and tourism	(Gursoy and Chi, 2020)	70	Effects of COVID-19 pandemic on hospitality industry	(Sharifi and Khavarian-Garmsir, 2020)	64	COVID-19 and urban planning	(Bae and Chang, 2021)	51	Tourism and COVID-19

**(Tobias et al., 2020)**	264	Air quality during the lockdown	(Mahato et al., 2020)	305	Air quality during the lockdown	(Dantas et al., 2020)	198	Air quality during the lockdown	(Sicard et al., 2020)	182	Air quality during the lockdown	(Collivignarelli et al., 2020)	181	Air quality during the lockdown	(Venter et al., 2020)	136	Air quality during the lockdown
**(Harris et al., 2020)**	258	Climate dataset	(Watts et al., 2021)	71	Health and climate change	(Friedlingstein et al., 2020)	68	Global carbon budget	(Burrell et al., 2020)	21	Climate change and desertification	(Haile et al., 2020)	14	Climate change and drought patterns	(Cucchi et al., 2020)	13	Space-time climate
**(Seibold et al., 2019)**	219	Arthropod decline in grasslands and forests	(Cardoso et al., 2020)	104	Insect extinctions	(Didham et al., 2020)	61	Insect extinctions	(Didham et al., 2020)	61	Insect extinctions	(Manenti et al., 2020)	35	Wildlife conservation	(Outhwaite et al., 2020)	34	Long-term biodiversity change
**(Zelinka et al., 2020)**	194	Global surface temperature response	(Sherwood et al., 2020)	83	Climate sensitivity/ocean warming contrast	(Notz et al., 2020)	58	Arctic sea ice	(Meehl et al., 2020)	56	Climate Sensitivity/ earth system models	(Kelley et al., 2020)	44	Atmospheric chemistry	(Lehner et al., 2020)	40	Partitioning climate projection uncertainty

Figure S-1. Beamplots of the first authors of the top 3 most cited papers (correctly classified) for CL and CC/GW/CE records. The blue lines indicate the mean citation percentile of papers published before their top cited paper, the magenta lines indicate the mean citation percentile of papers published after their top cited paper, and the green lines indicate the overall mean citation percentile.

**CL record**	**CC/GW/CE record**
Kalnay, Eugenia	Rayner, N. A
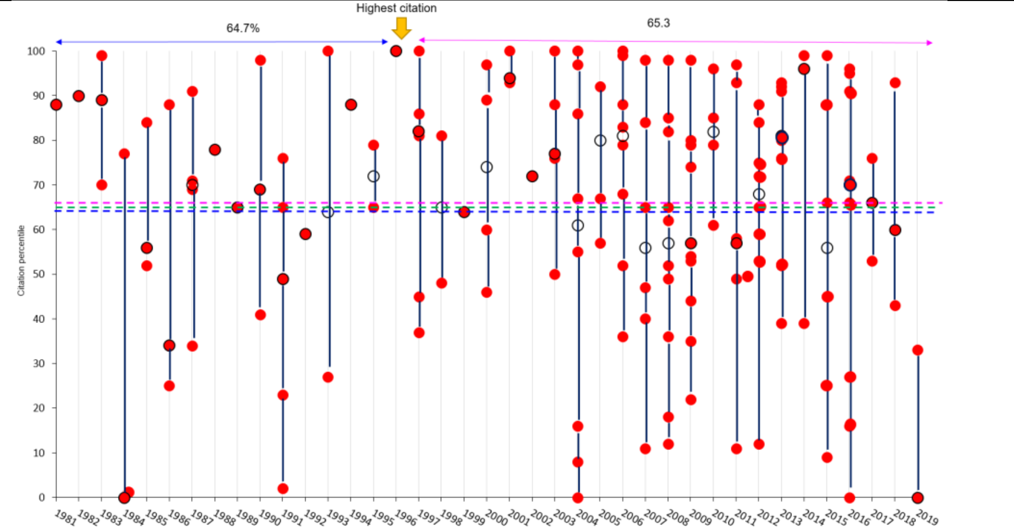	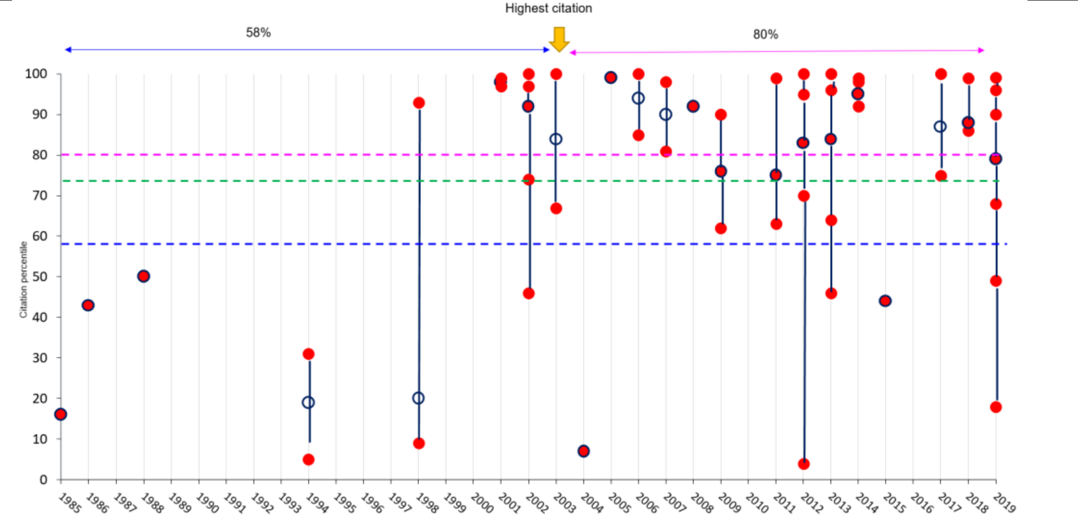

Hijmans, Robert J.

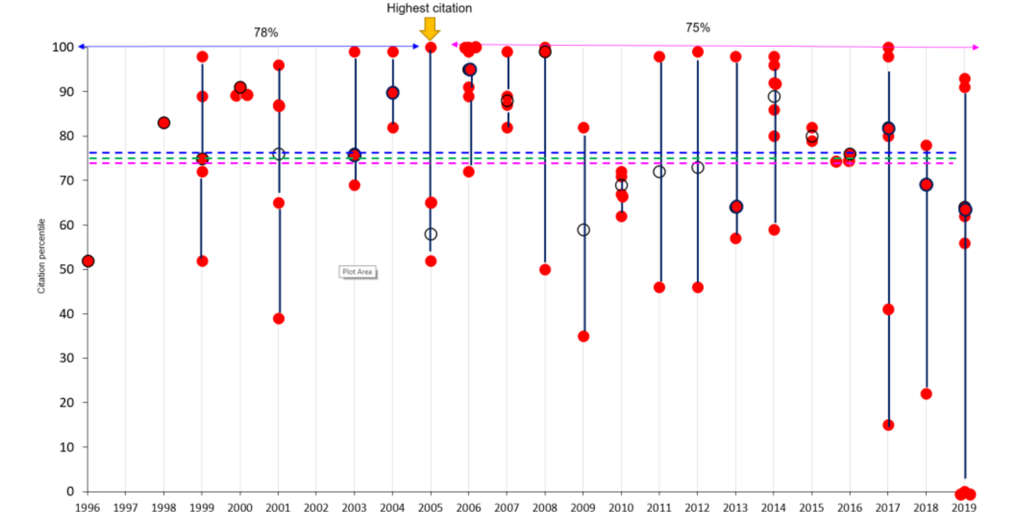

Parmesan, Camille

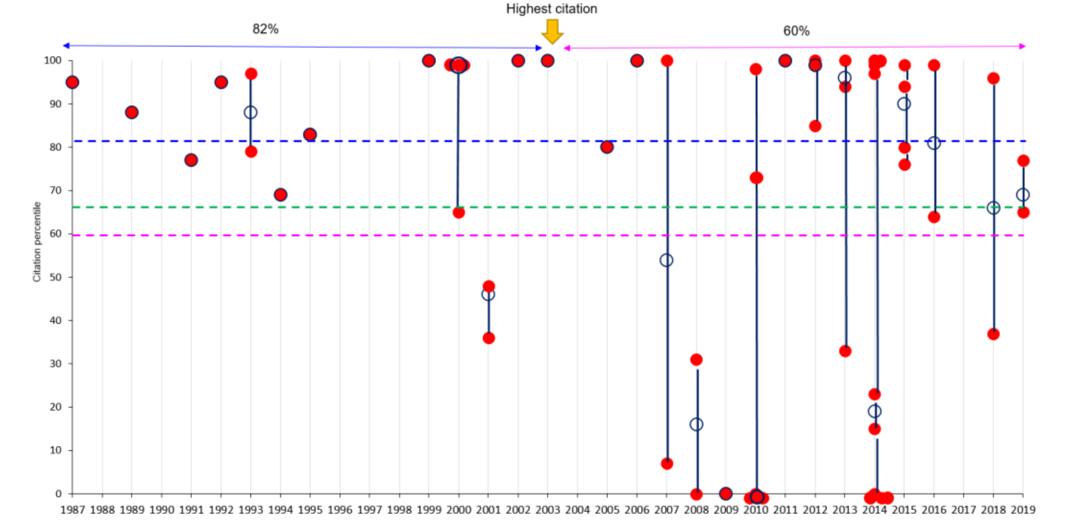

Phillips, Steven J.

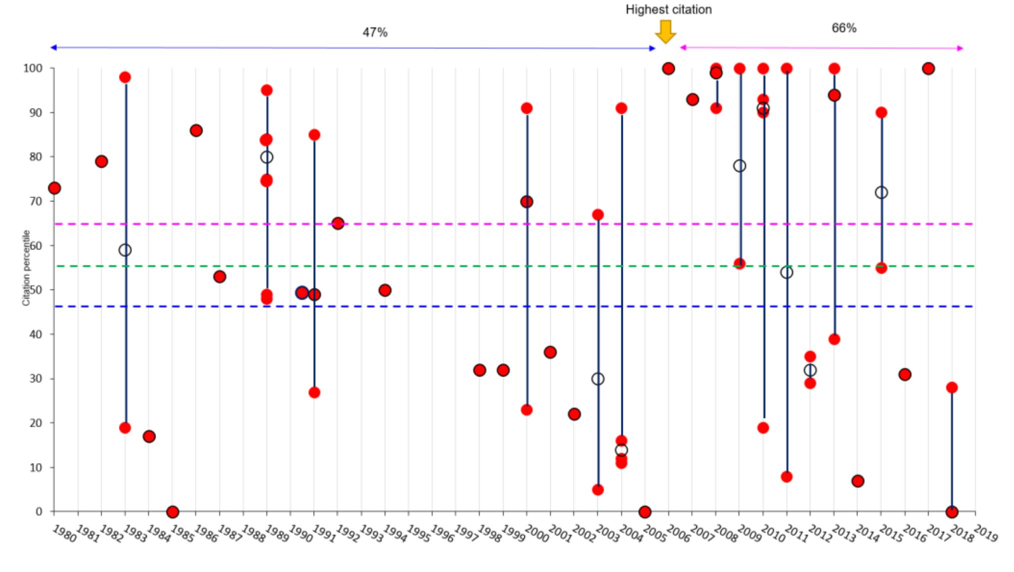

Thomas, Chris D

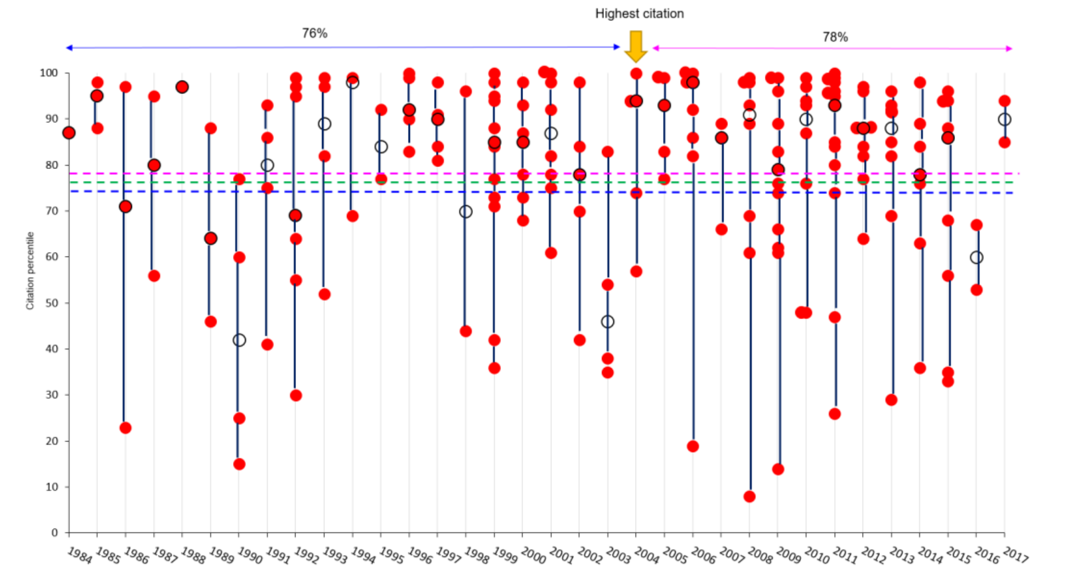

Figure S-2. Word dynamics and growth of the top 500 most cited papers for the CC/GW/CE and CL records.

CC/GW/CE

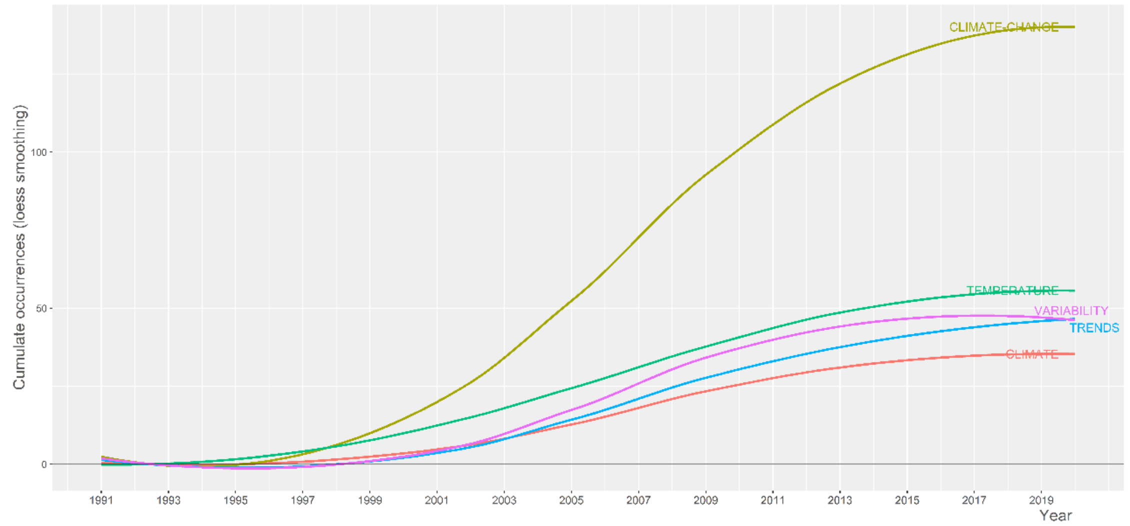

CL

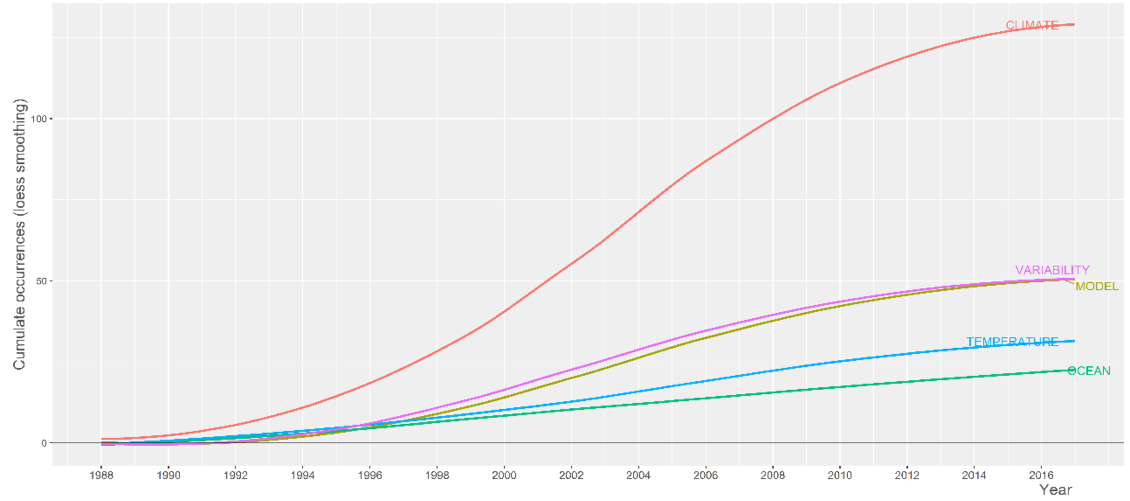

Figure S-3. Keywords co-occurrence of the top 500 most cited papers for the CC/GW/CE and CL records.

CC/GW/CE

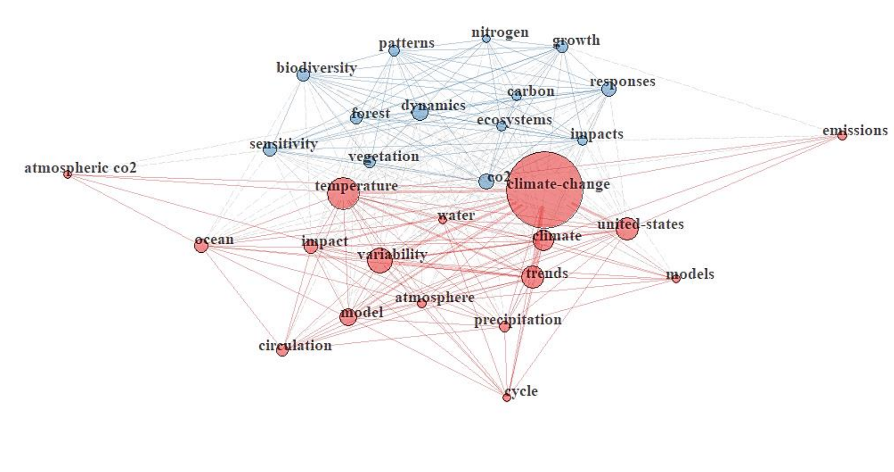

CL

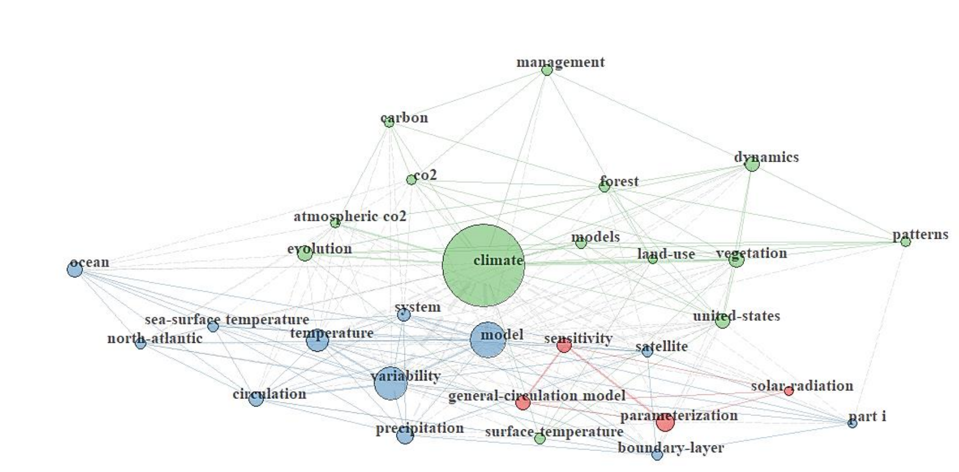

Table S-5. Data for number of articles in the CC/GW/CE and CL records, and the publication ratio ((CC/GW/CE)/(CL)) per year.

Year (↓)	CC/GW/CE	CL	ratio	Year (↓)	CC/GW/CE	CL	ratio	Year (↓)	CC/GW/CE	CL	ratio	Year (↓)	CC/GW/CE	CL	ratio
**2020**	37,336	23,866	1.56	**1989**	134	294	0.46	**1958**	0	17	0.00	**1927**	0	8	0.00
**2019**	32,091	21,612	1.48	**1988**	38	245	0.16	**1957**	1	25	0.04	**1926**	0	4	0.00
**2018**	28,062	19,075	1.47	**1987**	18	208	0.09	**1956**	0	22	0.00	**1925**	0	10	0.00
**2017**	24,878	17,096	1.46	**1986**	17	214	0.08	**1955**	0	5	0.00	**1924**	0	8	0.00
**2016**	22,741	16,230	1.40	**1985**	10	193	0.05	**1954**	0	9	0.00	**1923**	0	3	0.00
**2015**	20,251	14,718	1.38	**1984**	9	240	0.04	**1953**	0	11	0.00	**1922**	0	10	0.00
**2014**	16,601	12,484	1.33	**1983**	17	204	0.08	**1952**	0	5	0.00	**1921**	0	5	0.00
**2013**	15,468	11,685	1.32	**1982**	13	223	0.06	**1951**	0	6	0.00	**1920**	0	8	0.00
**2012**	13,334	10,347	1.29	**1981**	11	173	0.06	**1950**	0	3	0.00	**1919**	0	4	0.00
**2011**	12,152	9,178	1.32	**1980**	8	176	0.05	**1949**	0	6	0.00	**1918**	0	3	0.00
**2010**	10,218	8,331	1.23	**1979**	5	173	0.03	**1948**	0	10	0.00	**1917**	0	2	0.00
**2009**	8,629	7,501	1.15	**1978**	8	162	0.05	**1947**	0	5	0.00	**1916**	0	6	0.00
**2008**	6,854	7,055	0.97	**1977**	10	141	0.07	**1946**	0	14	0.00	**1915**	0	8	0.00
**2007**	5,402	6,163	0.88	**1976**	2	133	0.02	**1945**	0	9	0.00	**1914**	0	1	0.00
**2006**	3,982	5,368	0.74	**1975**	4	105	0.04	**1944**	0	10	0.00	**1913**	0	6	0.00
**2005**	3,458	4,837	0.71	**1974**	2	95	0.02	**1943**	0	4	0.00	**1912**	0	4	0.00
**2004**	2,871	4,437	0.65	**1973**	1	87	0.01	**1942**	0	3	0.00	**1911**	0	4	0.00
**2003**	2,590	4,146	0.62	**1972**	0	76	0.00	**1941**	0	3	0.00	**1910**	1	2	0.50
**2002**	2,195	3,756	0.58	**1971**	5	69	0.07	**1940**	0	8	0.00	**1909**	0	1	0.00
**2001**	2,126	3,475	0.61	**1970**	1	62	0.02	**1939**	1	10	0.10	**1908**	0	7	0.00
**2000**	1,877	3,208	0.59	**1969**	1	59	0.02	**1938**	0	10	0.00	**1907**	0	5	0.00
**1999**	1,705	3,082	0.55	**1968**	0	65	0.00	**1937**	0	7	0.00	**1906**	0	3	0.00
**1998**	1,576	2,709	0.58	**1967**	0	41	0.00	**1936**	0	8	0.00	**1905**	0	5	0.00
**1997**	1,399	2,556	0.55	**1966**	0	40	0.00	**1935**	0	6	0.00	**1904**	0	6	0.00
**1996**	1,133	2,326	0.49	**1965**	0	27	0.00	**1934**	0	13	0.00	**1903**	0	1	0.00
**1995**	1,082	2,071	0.52	**1964**	0	39	0.00	**1933**	0	9	0.00	**1902**	0	3	0.00
**1994**	750	1,858	0.40	**1963**	0	23	0.00	**1932**	0	10	0.00	**1901**	0	3	0.00
**1993**	764	1,609	0.47	**1962**	0	17	0.00	**1931**	0	13	0.00	**1900**	0	3	0.00
**1992**	707	1,481	0.48	**1961**	0	32	0.00	**1930**	0	16	0.00				
**1991**	614	1,327	0.46	**1960**	0	13	0.00	**1929**	0	8	0.00				
**1990**	318	437	0.73	**1959**	1	18	0.06	**1928**	0	8	0.00				

Table S-6. Data for number of articles in the CC/GW/CE record per year for each WoS database that make up the Core Collection.

Year (↓)	A&HCI	CPCI-SSH	ESCI	SCI-EXPANDED	SSCI	CPCI-S	Year (↓)	A&HCI	CPCI-SSH	ESCI	SCI-EXPANDED	SSCI	CPCI-S
**2020**	350	14	3,128	27,263	8,180	245	**1987**	2	0	0	39	4	0
**2019**	287	36	2,379	23,370	7,030	362	**1986**	1	0	0	39	2	0
**2018**	258	44	1,894	20,629	5,815	465	**1985**	2	0	0	32	5	0
**2017**	317	31	1,659	18,029	4,688	331	**1984**	1	0	0	16	2	0
**2016**	263	45	1,554	16,524	3,771	421	**1983**	1	0	0	30	4	0
**2015**	262	51	1,332	14,517	3,104	248	**1982**	2	0	0	24	6	0
**2014**	237	2	0	13,095	2,871	111	**1981**	0	0	0	14	1	0
**2013**	189	1	0	12,162	2,592	139	**1980**	0	0	0	23	5	0
**2012**	201	3	0	10,285	2,282	143	**1979**	0	0	0	23	3	0
**2011**	155	3	0	8,901	1,974	220	**1978**	0	0	0	18	4	0
**2010**	138	35	0	7,526	1,608	328	**1977**	0	0	0	31	2	0
**2009**	124	44	0	5,908	1,159	454	**1976**	0	0	0	25	6	0
**2008**	79	71	0	5,093	764	437	**1975**	0	0	0	19	6	0
**2007**	60	32	0	4,075	511	391	**1974**	0	0	0	4	3	0
**2006**	35	47	0	3,223	384	387	**1973**	0	0	0	9	1	0
**2005**	38	14	0	2,861	266	389	**1972**	0	0	0	5	1	0
**2004**	26	13	0	2,472	170	345	**1971**	0	0	0	11	2	0
**2003**	20	21	0	2,113	190	257	**1970**	0	0	0	4	3	0
**2002**	8	8	0	1,864	138	266	**1969**	0	0	0	6	1	0
**2001**	22	8	0	1,684	162	230	**1968**	0	0	0	0	0	0
**2000**	18	13	0	1,539	177	199	**1967**	0	0	0	0	0	0
**1999**	10	10	0	1,466	149	156	**1966**	0	0	0	0	0	0
**1998**	6	20	0	1,218	133	166	**1965**	0	0	0	0	0	0
**1997**	11	4	0	1,195	81	209	**1964**	0	0	0	0	0	0
**1996**	9	9	0	1,005	98	227	**1963**	0	0	0	0	0	0
**1995**	3	8	0	930	96	187	**1962**	0	0	0	0	0	0
**1994**	9	9	0	711	87	89	**1961**	0	0	0	0	0	0
**1993**	6	11	0	686	97	109	**1960**	0	0	0	0	0	0
**1992**	7	1	0	629	64	50	**1959**	0	0	0	1	1	0
**1991**	1	3	0	504	67	44	**1958**	0	0	0	0	0	0
**1990**	3	0	0	206	34	18	**1957**	0	0	0	1	0	0
**1989**	1	0	0	89	16	2	**1956**	0	0	0	0	0	0
**1988**	2	0	0	43	3	0	**1955**	0	0	0	0	0	0

Table S-7. Data for number of articles in the CL record per year for each WoS database that make up the Core Collection.

Year (↓)	A&HCI	CPCI-SSH	ESCI	SCI-EXPANDED	SSCI	CPCI-S	Year (↓)	A&HCI	CPCI-SSH	ESCI	SCI-EXPANDED	SSCI	CPCI-S
**2020**	371	10	2,484	18,251	4,657	127	**1987**	7	0	0	162	38	0
**2019**	343	26	2,183	16,495	4,166	246	**1986**	5	0	0	170	44	0
**2018**	273	17	1,900	14,793	3,326	289	**1985**	11	0	0	144	41	0
**2017**	297	16	1,668	13,230	2,835	197	**1984**	7	0	0	203	42	0
**2016**	264	24	1,618	12,639	2,296	288	**1983**	11	0	0	163	44	0
**2015**	236	23	1,342	11,558	2,097	207	**1982**	7	0	0	177	49	0
**2014**	269	3	0	10,775	1,929	94	**1981**	6	0	0	141	47	0
**2013**	230	3	0	10,132	1,730	124	**1980**	10	0	0	137	41	0
**2012**	211	7	0	8,892	1,567	119	**1979**	8	0	0	131	44	0
**2011**	205	9	0	7,852	1,410	173	**1978**	8	0	0	117	51	0
**2010**	164	21	0	7,044	1,233	209	**1977**	4	0	0	105	45	0
**2009**	149	39	0	6,411	1,071	354	**1976**	5	0	0	99	56	0
**2008**	105	60	0	6,075	877	368	**1975**	2	0	0	78	31	0
**2007**	94	42	0	5,356	719	419	**1974**	0	0	0	58	42	0
**2006**	84	36	0	4,735	553	428	**1973**	0	0	0	66	25	0
**2005**	60	33	0	4,261	460	443	**1972**	0	0	0	63	21	0
**2004**	57	42	0	3,899	419	464	**1971**	0	0	0	45	29	0
**2003**	71	42	0	3,635	396	373	**1970**	0	0	0	43	26	0
**2002**	40	35	0	3,246	366	376	**1969**	0	0	0	48	19	0
**2001**	45	29	0	3,012	307	318	**1968**	0	0	0	48	27	0
**2000**	43	25	0	2,786	324	298	**1967**	0	0	0	34	12	0
**1999**	32	28	0	2,640	328	278	**1966**	0	0	0	27	19	0
**1998**	22	29	0	2,290	318	233	**1965**	0	0	0	14	15	0
**1997**	13	27	0	2,182	288	210	**1964**	0	0	0	21	22	0
**1996**	14	26	0	1,977	269	230	**1963**	0	0	0	11	12	0
**1995**	25	24	0	1,749	279	227	**1962**	0	0	0	11	8	0
**1994**	16	18	0	1,569	227	137	**1961**	0	0	0	15	17	0
**1993**	23	15	0	1,330	229	121	**1960**	0	0	0	4	9	0
**1992**	38	9	0	1,208	206	63	**1959**	0	0	0	10	10	0
**1991**	10	1	0	1,166	114	122	**1958**	0	0	0	8	9	0
**1990**	7	6	0	363	50	16	**1957**	0	0	0	10	17	0
**1989**	10	0	0	244	44	8	**1956**	0	0	0	16	7	0
**1988**	8	0	0	203	33	0	**1955**	0	0	0	5	0	0

Table S-8. Data for CC/GW/CE and CL records for countries/regions of publication with more than 1000 articles in the CC/GW/CE record.

Countries/Regions	CC/GW/CE (↓)	CL	Ratio	Countries/Regions	CC/GW/CE (↓)	CL	Ratio
**USA**	76,681	78,153	0.98	**PORTUGAL**	3,484	2,496	1.40
**PEOPLES R CHINA**	33,258	27,451	1.21	**NEW ZEALAND**	3,403	2,976	1.14
**ENGLAND**	26,893	21,033	1.28	**MEXICO**	3,031	2,381	1.27
**GERMANY**	21,871	20,908	1.05	**POLAND**	2,941	2,883	1.02
**AUSTRALIA**	20,063	13,930	1.44	**TURKEY**	2,293	2,674	0.86
**CANADA**	18,846	13,892	1.36	**CZECH REPUBLIC**	2,167	1,793	1.21
**FRANCE**	14,443	13,871	1.04	**IRAN**	2,158	2,365	0.91
**SPAIN**	12,112	10,952	1.11	**TAIWAN**	2,117	2,077	1.02
**ITALY**	10,543	9,000	1.17	**ARGENTINA**	2,116	2,465	0.86
**NETHERLANDS**	9,798	7,711	1.27	**CHILE**	2,048	1,740	1.18
**SWEDEN**	8,298	6,713	1.24	**WALES**	2,017	1,379	1.46
**SWITZERLAND**	8,214	6,784	1.21	**GREECE**	2,014	1,881	1.07
**JAPAN**	7,577	6,697	1.13	**MALAYSIA**	1,605	1,251	1.28
**INDIA**	7,314	6,709	1.09	**ISRAEL**	1,485	2,201	0.67
**NORWAY**	6,224	5,264	1.18	**PAKISTAN**	1,444	923	1.56
**BRAZIL**	6,012	6,633	0.91	**IRELAND**	1,427	1,159	1.23
**DENMARK**	5,130	3,738	1.37	**KENYA**	1,281	624	2.05
**SCOTLAND**	4,983	3,211	1.55	**HUNGARY**	1,229	1,008	1.22
**FINLAND**	4,512	3,544	1.27	**COLOMBIA**	1,180	828	1.43
**SOUTH KOREA**	4,372	3,424	1.28	**SINGAPORE**	1,172	912	1.29
**SOUTH AFRICA**	4,222	3,047	1.39	**ROMANIA**	1,157	1,052	1.10
**RUSSIA**	4,199	5,194	0.81	**THAILAND**	1,144	807	1.42
**AUSTRIA**	4,091	3,024	1.35	**SAUDI ARABIA**	1,098	1,090	1.01
**BELGIUM**	3,823	3,678	1.04	**INDONESIA**	1,020	723	1.41

Table S-9. Data for CC/GW/CE and CL records for countries/regions that no longer exist and have been merged with their successor countries.

**Before merging**				**After merging**			
**Countries/Regions**	**CC/GW/CE**	**CL**	**Ratio**	**Countries/Regions**	**CC/GW/CE**	**CL**	**Ratio**
**CZECHOSLOVAKIA**	5	37	0.14	**RUSSIA**	3,329	5,999	0.55
**RUSSIA**	3,314	5,811	0.57	**CZECH REPUBLIC**	1,973	1,952	1.01
**SERBIA**	432	596	0.72	**GERMANY**	20,287	22,124	0.92
**GERMANY**	20,285	21,966	0.92	**ESWATINI**	26	10	2.6
**FED REP GER**	2	149	0.01	**SERBIA**	434	611	0.71
**SWAZILAND**	12	6	2.00				
**ESWATINI**	14	4	3.50				
**CZECH REPUBLIC**	1,968	1,915	1.03				
**USSR**	15	188	0.08				
**YUGOSLAVIA**	1	13	0.08				

Figure S-4. Data for CC/GW/CE and CL records for country/region of publication: a) Publication ratio ((CC/GW/CE)/(CL)) for top 105 countries/regions, ordered from highest to lowest ratio; b) Publication ratio ((CC/GW/CE)/(CL)) for bottom 105 countries/regions, ordered from highest to lowest ratio.

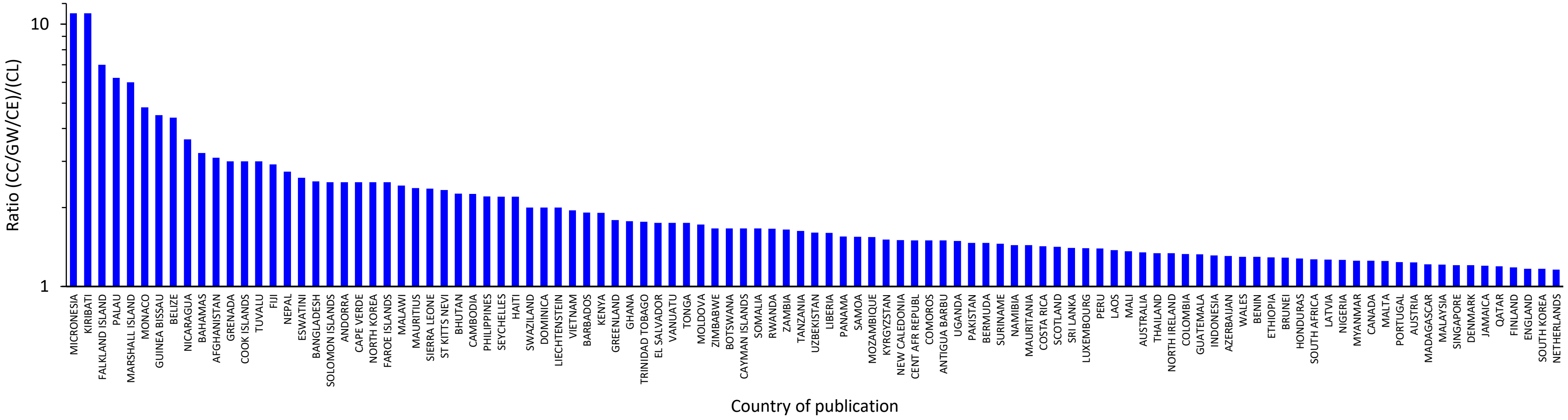

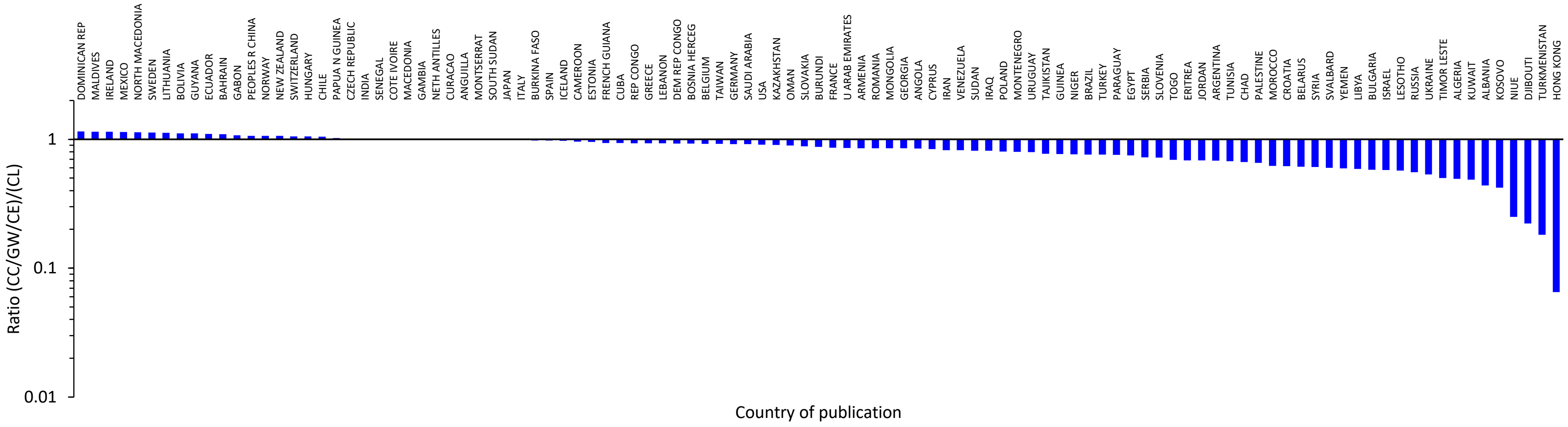

Figure S-5. Countries' scientific production of the top 500 most cited papers for CC/GW/CE and CL records.

CC/GW/CE	CL
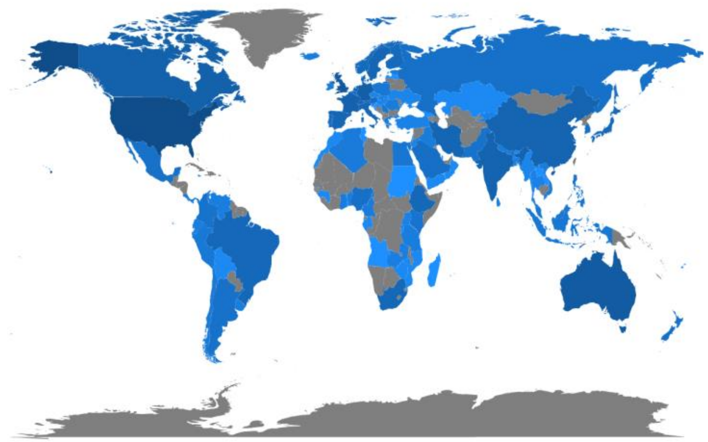	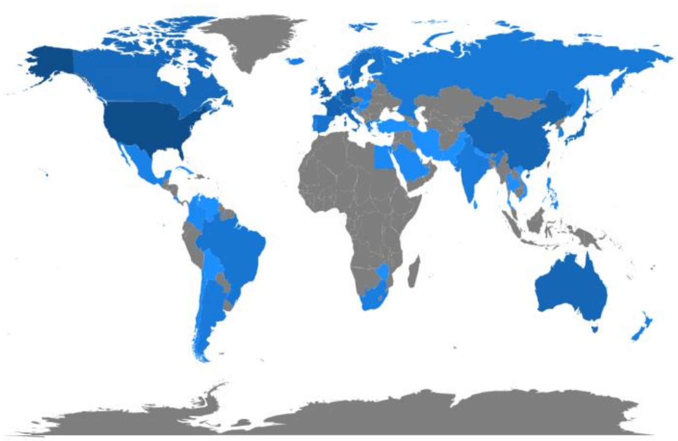

Figure S-6. The countries of collaboration for the top 5 most cited papers in CC/GW/CE and CL records.

<table>
<tr><th>Author</th><th>CC/GW/CE Record</th><th>Author</th><th>CL Record</th></tr>
<tr><td>Rayner, Nick A.</td><td>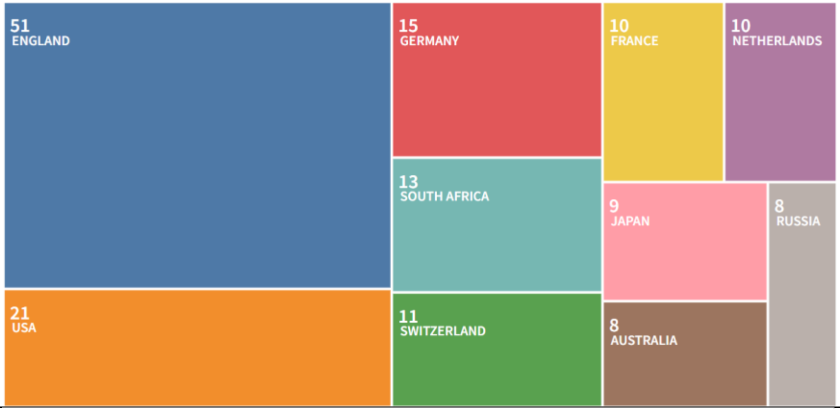
</td><td>Kalnay, Eugenia</td><td>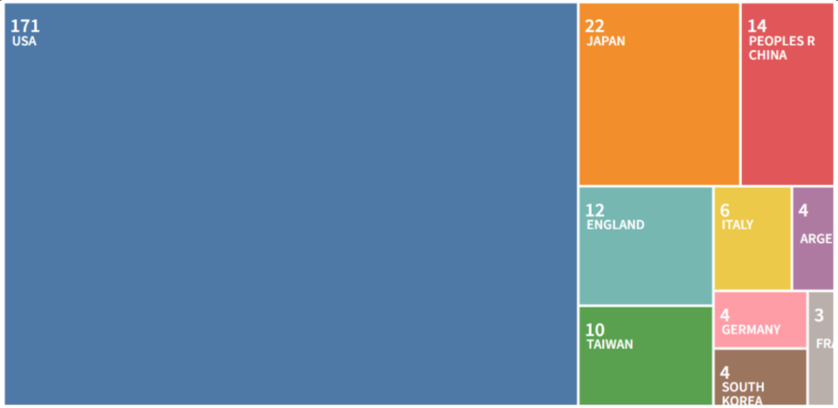
</td></tr>
<tr><td>Parmesan, Camille</td><td>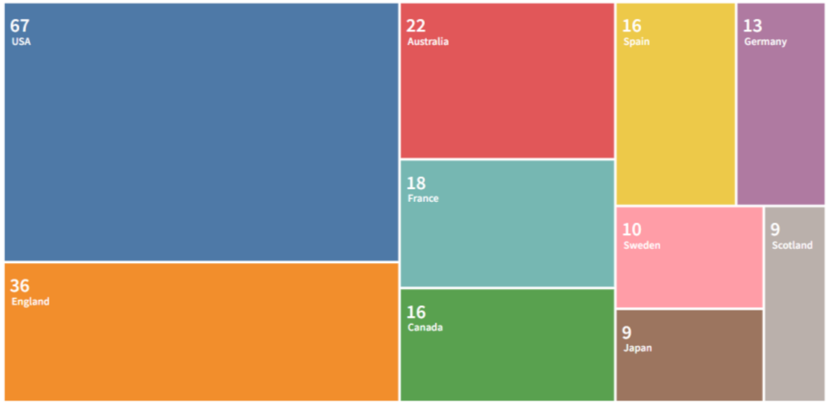
</td><td>Hijmans, Robert J.</td><td>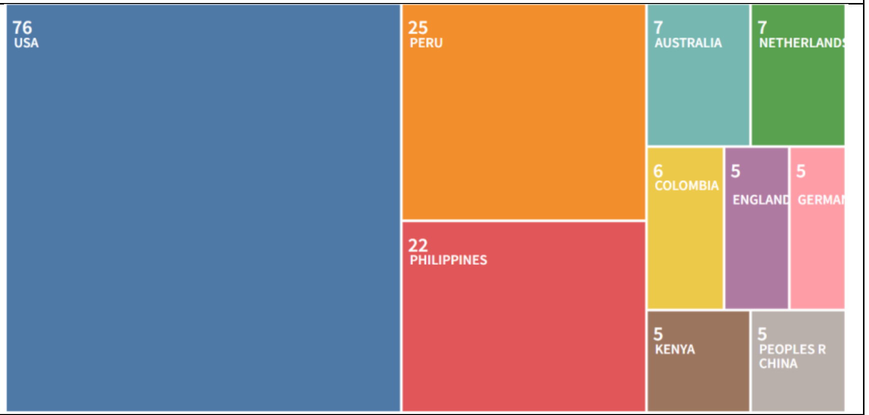
</td></tr>
</table>

Kottek, Markus

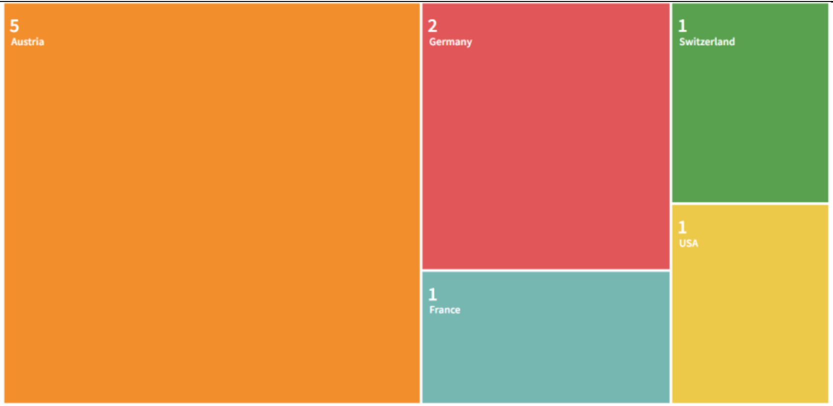

Taylor, Karl E.

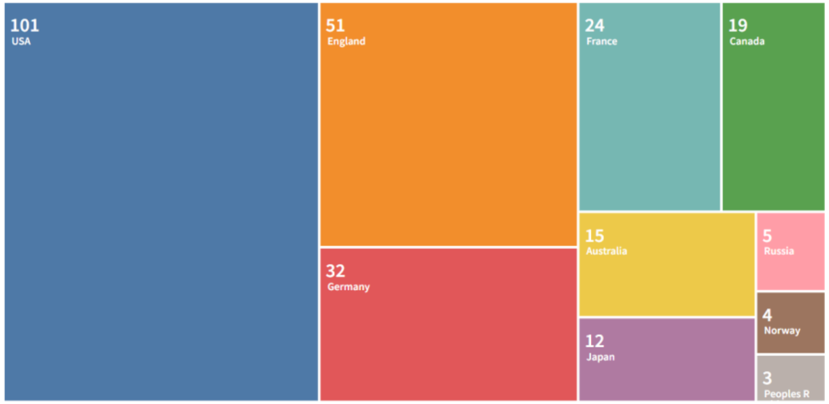

Thomas, Chris D.

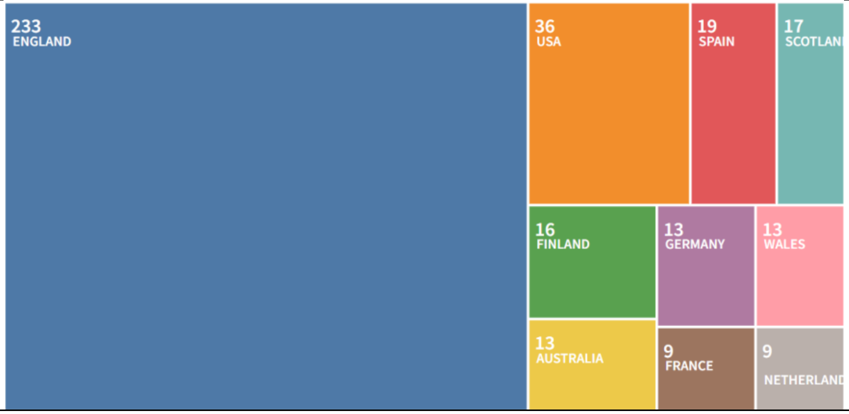

Phillips, Steven J.

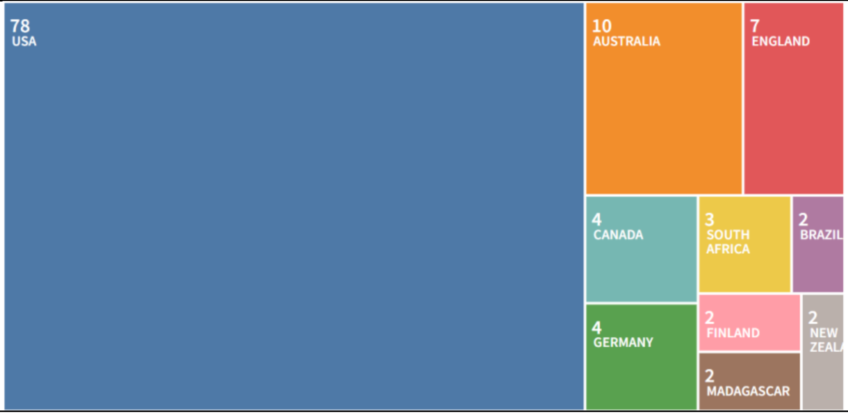

<table>
<tr>
<td>Allen,
Craig D.</td>
<td>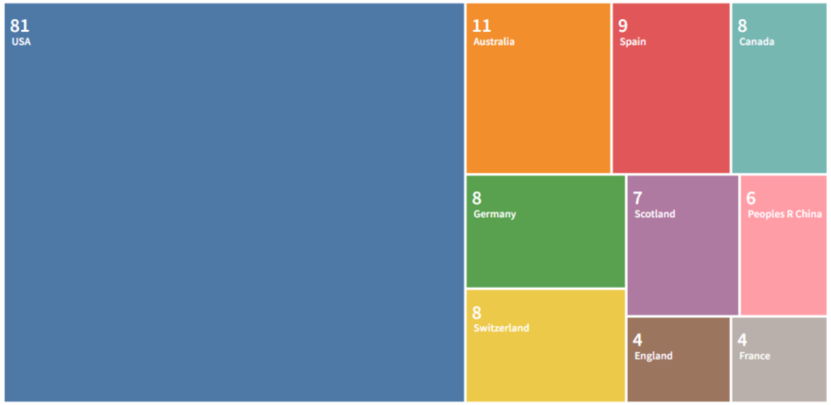
</td>
<td>Tenenbaum,
Joshua B.</td>
<td>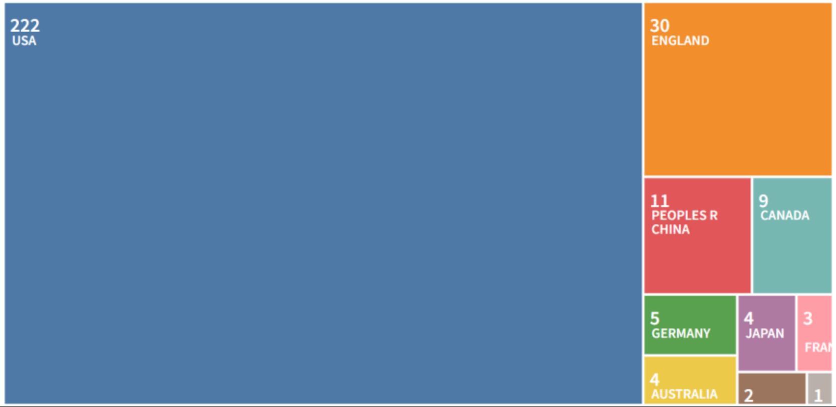
</td>
</tr>
</table>

Figure S-7. Country collaboration networks of the top 500 most cited papers for CC/GW/CE and CL records.

CC/GW/CE	CL
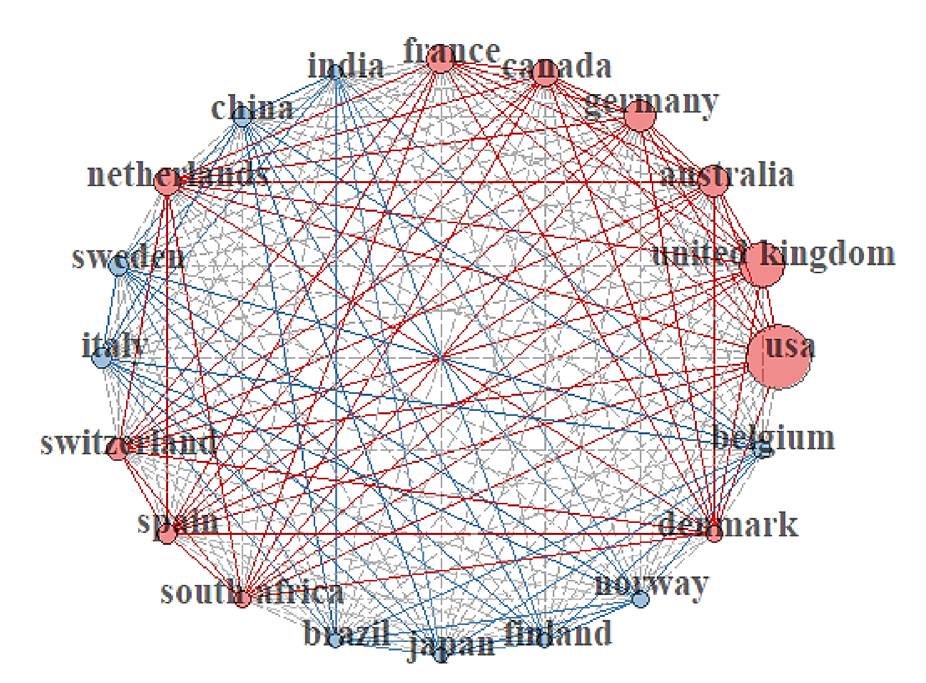	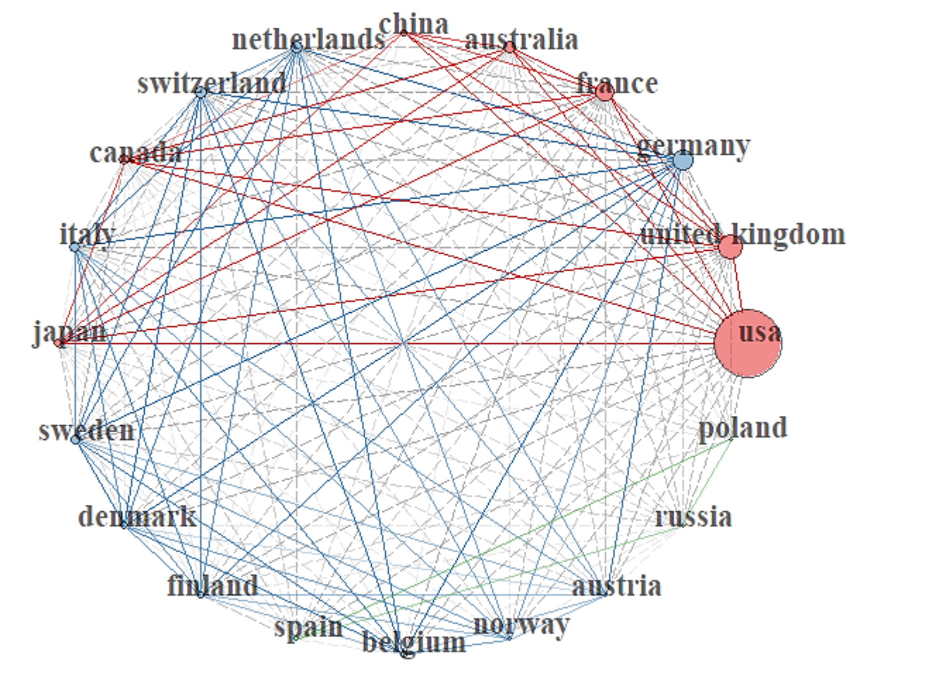

Figure S-8. Country collaboration maps of the top 500 most cited papers for CC/GW/CE and CL records.

CC/GW/CE	CL
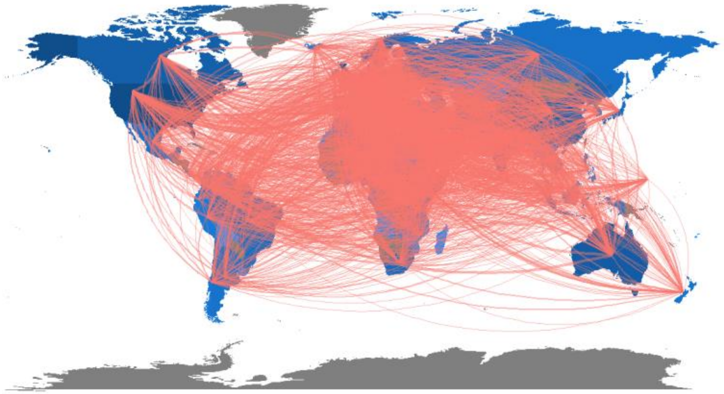	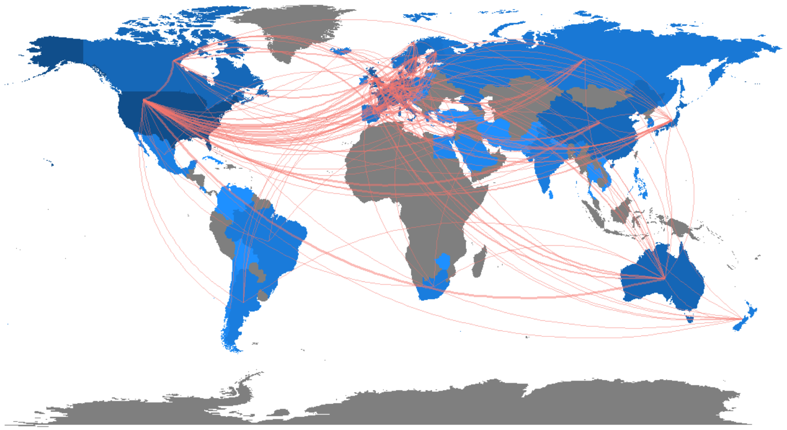

R-codes for the analyses and visualizations of this paper, based on 'bibliometrix' package (https://cran.r-project.org/web/packages/bibliometrix/bibliometrix.pdf).

```
library(bibliometrix)

#Data loading and converting

D <- ("C:/……")

#the object D can be converted in a data frame using the function convert2df

M <- convert2df(D, dbsource = "wos", format = "bibtex")

#Bibliometric Analysis

results <- biblioAnalysis(M, sep = ";")

S <- summary(object = results, k = 10, pause = FALSE) #summary and plot of results

plot(x = results, k = 10, pause = FALSE)

#Analysis of Cited References

M$CR[1]

CR <- citations(M, field = "article", sep = ";") #To obtain the most frequent cited manuscripts in the sample

CR$Cited[1:10]

CR <- citations(M, field = "author", sep = ";") #To obtain the most frequent cited first authors

CR$Cited[1:10]

CR <- citations(M, field = "Sources", sep = ";") #To obtain the most frequent cited first authors

CR$Cited[1:10]

CR <- localCitations(M, sep = "; ") #To obtain the most frequent local cited authors

#to run vosviewer in bibliometrix

#net=networkPlot(NetMatrix, type= "vosviewer", normalize = "association", cluster= "louvain",  n = 50, vos.path="C:/Users/ziaul/Documents/VOSviewer.jar")

#Bibliographic co-citation

NetMatrix <- biblioNetwork(M, analysis = "co-citation", network = "references", sep = ";")

net <- networkPlot(NetMatrix, n = 20, type = "kamada", Title = "Co-Citation",labelsize=0.5)
```

```
##Bibliographic collaboration

#authors' collaboration network:

NetMatrix <- biblioNetwork(M, analysis = "collaboration", network = "authors", sep = ";")

net <- networkPlot(NetMatrix, n = 20, type = "kamada", Title = "Author collaboration",labelsize=1)

#or a country collaboration network:

NetMatrix <- biblioNetwork(M, analysis = "collaboration", network = "countries", sep = ";")

net <- networkPlot(NetMatrix, n = 20, type = "kamada", Title = "Country collaboration",labelsize=1)


####

#Visualizing bibliographic networks

# Create a country collaboration network

M <- metaTagExtraction(M, Field = "AU_CO", sep = ";")

NetMatrix <- biblioNetwork(M, analysis = "collaboration", network = "countries", sep = ";")

# Plot the network

net=networkPlot(NetMatrix, n = 20, Title = "Country Collaboration", type = "circle", size=TRUE, remove.multiple=FALSE,labelsize=1.2)

#####Co-Citation Network

# Create a co-citation network

NetMatrix <- biblioNetwork(M, analysis = "co-citation", network = "references", sep = ";")

# Plot the network

net=networkPlot(NetMatrix, n = 20, Title = "Co-Citation Network", type = "fruchterman", size=T, remove.multiple=FALSE, labelsize=0.7,label.short = TRUE, edgesize = 5)


##Keyword co-occurrences

# Create keyword co-occurrencies network

NetMatrix <- biblioNetwork(M, analysis = "co-occurrences", network = "keywords", sep = ";")
```

```
# Plot the network

net=networkPlot(NetMatrix, normalize="association", weighted=T, n = 30, Title = "Keyword Co-occurrences", type = "fruchterman", size=T,edgesize = 5,labelsize=1)


#### Conceptual Structure using keywords

CS <- conceptualStructure(M, method="CA", field="ID", minDegree=2, k.max = 5, stemming=f, labelsize=8,documents=20)

Clusters=Map$words[order(Map$words$Cluster,-Map$words$Occurrences),]

### Create a historical citation network

histResults <- histNetwork(M, n = 20, sep = ".  ") #works with WOS data only


# Plot a historical co-citation network

net <- histPlot(histResults, size = FALSE,label=TRUE, arrowsize = 0.5)#works with WOS data only
```

References:

ADGER, W. N. 2006. Vulnerability. *Global Environmental Change-Human and Policy Dimensions,* 16**,** 268-281.

ALEXANDER, L. V., ZHANG, X., PETERSON, T. C., CAESAR, J., GLEASON, B., TANK, A., HAYLOCK, M., COLLINS, D., TREWIN, B., RAHIMZADEH, F., TAGIPOUR, A., KUMAR, K. R., REVADEKAR, J., GRIFFITHS, G., VINCENT, L., STEPHENSON, D. B., BURN, J., AGUILAR, E., BRUNET, M., TAYLOR, M., NEW, M., ZHAI, P., RUSTICUCCI, M. & VAZQUEZ-AGUIRRE, J. L. 2006. Global observed changes in daily climate extremes of temperature and precipitation. *J. Geophys. Res.-Atmos.,* 111.

ALLEN, C. D., BRESHEARS, D. D. & MCDOWELL, N. G. 2015. On underestimation of global vulnerability to tree mortality and forest die-off from hotter drought in the Anthropocene. *Ecosphere,* 6**,** 55.

ALLEN, C. D., MACALADY, A. K., CHENCHOUNI, H., BACHELET, D., MCDOWELL, N., VENNETIER, M., KITZBERGER, T., RIGLING, A., BRESHEARS, D. D., HOGG, E. H., GONZALEZ, P., FENSHAM, R., ZHANG, Z., CASTRO, J., DEMIDOVA, N., LIM, J. H., ALLARD, G., RUNNING, S. W., SEMERCI, A. & COBB, N. 2010a. A global overview of drought and heat-induced tree mortality reveals emerging climate change risks for forests. *Forest Ecology and Management,* 259**,** 660-684.

ALLEN, C. D., MACALADY, A. K., CHENCHOUNI, H., BACHELET, D., MCDOWELL, N., VENNETIER, M., KITZBERGER, T., RIGLING, A., BRESHEARS, D. D. & HOGG, E. T. 2010b. A global overview of drought and heat-induced tree mortality reveals emerging climate change risks for forests. *Forest ecology and management,* 259**,** 660-684.

ALLOUCHE, O., TSOAR, A. & KADMON, R. 2006. Assessing the accuracy of species distribution models: prevalence, kappa and the true skill statistic (TSS). *Journal of Applied Ecology,* 43**,** 1223-1232.

ALVARES, C. A., STAPE, J. L., SENTELHAS, P. C., GONÇALVES, J. D. M. & SPAROVEK, G. 2013a. Köppen's climate classification map for Brazil. *Meteorol. Z.,* 22**,** 711-728.

ALVARES, C. A., STAPE, J. L., SENTELHAS, P. C., GONCALVES, J. L. D. & SPAROVEK, G. 2013b. Koppen's climate classification map for Brazil. *Meteorol. Z.,* 22**,** 711-728.

ALYAMI, M., HENNING, M., KRAGELOH, C. U. & ALYAMI, H. Psychometric Evaluation of the Arabic Version of the Fear of COVID-19 Scale. *Int. J. Mental Health Addict.***,** 14.

AMABILE, T. M., CONTI, R., COON, H., LAZENBY, J. & HERRON, M. 1996. Assessing the work environment for creativity. *Acad. Manage. J.,* 39**,** 1154-1184.

ARAGAW, T. A. 2020. Surgical face masks as a potential source for microplastic pollution in the COVID-19 scenario. *Mar. Pollut. Bull.,* 159**,** 7.

ARNOLD, J. G., SRINIVASAN, R., MUTTIAH, R. S. & WILLIAMS, J. R. 1998. Large area hydrologic modeling and assessment - Part 1: Model development. *J. Am. Water Resour. Assoc.,* 34**,** 73-89.

BAE, S. Y. & CHANG, P. J. 2021. The effect of coronavirus disease-19 (COVID-19) risk perception on behavioural intention towards 'untact' tourism in South Korea during the first wave of the pandemic (March 2020). *Curr. Issues Tour.,* 24**,** 1017-1035.

BARNETT, T. P., ADAM, J. C. & LETTENMAIER, D. P. 2005. Potential impacts of a warming climate on water availability in snow-dominated regions. *Nature,* 438**,** 303-309.

BASTIN, J. F., FINEGOLD, Y., GARCIA, C., MOLLICONE, D., REZENDE, M., ROUTH, D., ZOHNER, C. M. & CROWTHER, T. W. 2019. The global tree restoration potential. *Science,* 365**,** 76-+.

BATJES, N. H. 1996. Total carbon and nitrogen in the soils of the world. *European Journal of Soil Science,* 47**,** 151-163.
BATJES, N. H. 2014. Total carbon and nitrogen in the soils of the world. *European Journal of Soil Science,* 65**,** 10-21.
BECK, H. E., ZIMMERMANN, N. E., MCVICAR, T. R., VERGOPOLAN, N., BERG, A. & WOOD, E. F. 2018. Present and future Koppen-Geiger climate classification maps at 1-km resolution. *Scientific Data,* 5.
BELKIN, M. & NIYOGI, P. 2003. Laplacian eigenmaps for dimensionality reduction and data representation. *Neural computation,* 15**,** 1373-1396.
BENGIO, Y., COURVILLE, A. & VINCENT, P. 2013. Representation learning: A review and new perspectives. *IEEE transactions on pattern analysis and machine intelligence,* 35**,** 1798-1828.
BERGER, A. & LOUTRE, M. F. 1991. INSOLATION VALUES FOR THE CLIMATE OF THE LAST 10000000 YEARS. *Quat. Sci. Rev.,* 10**,** 297-317.
BOND, T. C., DOHERTY, S. J., FAHEY, D. W., FORSTER, P. M., BERNTSEN, T., DEANGELO, B. J., FLANNER, M. G., GHAN, S., KARCHER, B., KOCH, D., KINNE, S., KONDO, Y., QUINN, P. K., SAROFIM, M. C., SCHULTZ, M. G., SCHULZ, M., VENKATARAMAN, C., ZHANG, H., ZHANG, S., BELLOUIN, N., GUTTIKUNDA, S. K., HOPKE, P. K., JACOBSON, M. Z., KAISER, J. W., KLIMONT, Z., LOHMANN, U., SCHWARZ, J. P., SHINDELL, D., STORELVMO, T., WARREN, S. G. & ZENDER, C. S. 2013. Bounding the role of black carbon in the climate system: A scientific assessment. *J. Geophys. Res.-Atmos.,* 118**,** 5380-5552.
BOUCHER, O., SERVONNAT, J., ALBRIGHT, A. L., AUMONT, O., BALKANSKI, Y., BASTRIKOV, V., BEKKI, S., BONNET, R., BONY, S., BOPP, L., BRACONNOT, P., BROCKMANN, P., CADULE, P., CAUBEL, A., CHERUY, F., CODRON, F., COZIC, A., CUGNET, D., D'ANDREA, F., DAVINI, P., DE LAVERGNE, C., DENVIL, S., DESHAYES, J., DEVILLIERS, M., DUCHARNE, A., DUFRESNE, J. L., DUPONT, E., ETHE, C., FAIRHEAD, L., FALLETTI, L., FLAVONI, S., FOUJOLS, M. A., GARDOLL, S., GASTINEAU, G., GHATTAS, J., GRANDPEIX, J. Y., GUENET, B., GUEZ, L. E., GUILYARDI, E., GUIMBERTEAU, M., HAUGLUSTAINE, D., HOURDIN, F., IDELKADI, A., JOUSSAUME, S., KAGEYAMA, M., KHODRI, M., KRINNER, G., LEBAS, N., LEVAVASSEUR, G., LEVY, C., LI, L., LOTT, F., LURTON, T., LUYSSAERT, S., MADEC, G., MADELEINE, J. B., MAIGNAN, F., MARCHAND, M., MARTI, O., MELLUL, L., MEURDESOIF, Y., MIGNOT, J., MUSAT, I., OTTLE, C., PEYLIN, P., PLANTON, Y., POLCHER, J., RIO, C., ROCHETIN, N., ROUSSET, C., SEPULCHRE, P., SIMA, A., SWINGEDOUW, D., THIEBLEMONT, R., TRAORE, A. K., VANCOPPENOLLE, M., VIAL, J., VIALARD, J., VIOVY, N. & VUICHARD, N. 2020. Presentation and Evaluation of the IPSL-CM6A-LR Climate Model. *J. Adv. Model. Earth Syst.,* 12**,** 52.
BURRELL, A. L., EVANS, J. P. & DE KAUWE, M. G. 2020. Anthropogenic climate change has driven over 5 million km(2) of drylands towards desertification. *Nat. Commun.,* 11**,** 11.
BUTCHART, S. H. M., WALPOLE, M., COLLEN, B., VAN STRIEN, A., SCHARLEMANN, J. P. W., ALMOND, R. E. A., BAILLIE, J. E. M., BOMHARD, B., BROWN, C., BRUNO, J., CARPENTER, K. E., CARR, G. M., CHANSON, J., CHENERY, A. M., CSIRKE, J., DAVIDSON, N. C., DENTENER, F., FOSTER, M., GALLI, A., GALLOWAY, J. N., GENOVESI, P., GREGORY, R. D., HOCKINGS, M., KAPOS, V., LAMARQUE, J. F., LEVERINGTON, F., LOH, J., MCGEOCH, M. A., MCRAE, L., MINASYAN, A., MORCILLO, M. H., OLDFIELD, T. E. E., PAULY, D., QUADER, S., REVENGA, C., SAUER, J. R., SKOLNIK, B., SPEAR, D., STANWELL-SMITH, D., STUART, S. N., SYMES, A., TIERNEY, M., TYRRELL, T. D., VIE, J. C. & WATSON, R. 2010. Global Biodiversity: Indicators of Recent Declines. *Science,* 328**,** 1164-1168.
CARDOSO, P., BARTON, P. S., BIRKHOFER, K., CHICHORRO, F., DEACON, C., FARTMANN, T., FUKUSHIMA, C. S., GAIGHER, R., HABEL, J. C., HALLMANN, C. A., HILL, M. J., HOCHKIRCH, A., KWAK, M. L., MAMMOLA, S., NORIEGA, J. A., ORFINGER, A. B., PEDRAZA, F., PRYKE, J. S., ROQUE, F. O., SETTELE, J., SIMAIKA, J. P., STORK, N. E., SUHLING, F., VORSTER, C. & SAMWAYS, M. J. 2020. Scientists' warning to humanity on insect extinctions. *Biol. Conserv.,* 242**,** 12.

CHARLSON, R. J., LOVELOCK, J. E., ANDREAE, M. O. & WARREN, S. G. 1987. OCEANIC PHYTOPLANKTON, ATMOSPHERIC SULFUR, CLOUD ALBEDO AND CLIMATE. *Nature,* 326**,** 655-661.

CHARLSON, R. J., SCHWARTZ, S. E., HALES, J. M., CESS, R. D., COAKLEY, J. A., HANSEN, J. E. & HOFMANN, D. J. 1992. CLIMATE FORCING BY ANTHROPOGENIC AEROSOLS. *Science,* 255**,** 423-430.

CHAZDON, R. & BRANCALION, P. 2019. Restoring forests as a means to many ends. *Science,* 365**,** 24-25.

CHEN, F. & DUDHIA, J. 2001a. Coupling an advanced land surface-hydrology model with the Penn State-NCAR MM5 modeling system. Part I: Model implementation and sensitivity. *Mon. Weather Rev.,* 129**,** 569-585.

CHEN, F. & DUDHIA, J. 2001b. Coupling an advanced land surface–hydrology model with the Penn State–NCAR MM5 modeling system. Part I: Model implementation and sensitivity. *Mon. Weather Rev.,* 129**,** 569-585.

CHEN, I.-C., HILL, J. K., OHLEMÜLLER, R., ROY, D. B. & THOMAS, C. D. 2011a. Rapid range shifts of species associated with high levels of climate warming. *Science,* 333**,** 1024-1026.

CHEN, I. C., HILL, J. K., OHLEMULLER, R., ROY, D. B. & THOMAS, C. D. 2011b. Rapid Range Shifts of Species Associated with High Levels of Climate Warming. *Science,* 333**,** 1024-1026.

CHOAT, B., JANSEN, S., BRODRIBB, T. J., COCHARD, H., DELZON, S., BHASKAR, R., BUCCI, S. J., FEILD, T. S., GLEASON, S. M., HACKE, U. G., JACOBSEN, A. L., LENS, F., MAHERALI, H., MARTINEZ-VILALTA, J., MAYR, S., MENCUCCINI, M., MITCHELL, P. J., NARDINI, A., PITTERMANN, J., PRATT, R. B., SPERRY, J. S., WESTOBY, M., WRIGHT, I. J. & ZANNE, A. E. 2012. Global convergence in the vulnerability of forests to drought. *Nature,* 491**,** 752-+.

CIAIS, P., SABINE, C., BALA, G., BOPP, L., BROVKIN, V., CANADELL, J., CHHABRA, A., DEFRIES, R., GALLOWAY, J. & HEIMANN, M. 2014. Carbon and other biogeochemical cycles. *Climate change 2013: the physical science basis. Contribution of Working Group I to the Fifth Assessment Report of the Intergovernmental Panel on Climate Change.* Cambridge University Press.

COLLINS, M., KNUTTI, R., ARBLASTER, J., DUFRESNE, J.-L., FICHEFET, T., FRIEDLINGSTEIN, P., GAO, X., GUTOWSKI, W. J., JOHNS, T. & KRINNER, G. 2013. Long-term climate change: projections, commitments and irreversibility. *Climate Change 2013-The Physical Science Basis: Contribution of Working Group I to the Fifth Assessment Report of the Intergovernmental Panel on Climate Change.* Cambridge University Press.

COLLINS, W. D., BITZ, C. M., BLACKMON, M. L., BONAN, G. B., BRETHERTON, C. S., CARTON, J. A., CHANG, P., DONEY, S. C., HACK, J. J., HENDERSON, T. B., KIEHL, J. T., LARGE, W. G., MCKENNA, D. S., SANTER, B. D. & SMITH, R. D. 2006. The Community Climate System Model version 3 (CCSM3). *Journal of Climate,* 19**,** 2122-2143.

COLLIVIGNARELLI, M. C., ABBA, A., BERTANZA, G., PEDRAZZANI, R., RICCIARDI, P. & MIINO, M. C. 2020. Lockdown for CoViD-2019 in Milan: What are the effects on air quality? *Science of the Total Environment,* 732**,** 9.

COMPO, G. P., WHITAKER, J. S., SARDESHMUKH, P. D., MATSUI, N., ALLAN, R. J., YIN, X., GLEASON, B. E., VOSE, R. S., RUTLEDGE, G. & BESSEMOULIN, P. 2011. The twentieth century reanalysis project. *Quarterly Journal of the Royal Meteorological Society,* 137**,** 1-28.

COX, P. M., BETTS, R. A., JONES, C. D., SPALL, S. A. & TOTTERDELL, I. J. 2000. Acceleration of global warming due to carbon-cycle feedbacks in a coupled climate model. *Nature,* 408**,** 184-187.

CUCCHI, M., WEEDON, G. P., AMICI, A., BELLOUIN, N., LANGE, S., SCHMIED, H. M., HERSBACH, H. & BUONTEMPO, C. 2020. WFDE5: bias-adjusted ERA5 reanalysis data for impact studies. *Earth Syst. Sci. Data,* 12**,** 2097-2120.

DAI, A. G. 2013. Increasing drought under global warming in observations and models. *Nature Climate Change,* 3**,** 52-58.
DALY, C., HALBLEIB, M., SMITH, J. I., GIBSON, W. P., DOGGETT, M. K., TAYLOR, G. H., CURTIS, J. & PASTERIS, P. P. 2008. Physiographically sensitive mapping of climatological temperature and precipitation across the conterminous United States. *International Journal of Climatology: a Journal of the Royal Meteorological Society,* 28**,** 2031-2064.
DANSGAARD, W., JOHNSEN, S. J., CLAUSEN, H. B., DAHLJENSEN, D., GUNDESTRUP, N. S., HAMMER, C. U., HVIDBERG, C. S., STEFFENSEN, J. P., SVEINBJORNSDOTTIR, A. E., JOUZEL, J. & BOND, G. 1993. EVIDENCE FOR GENERAL INSTABILITY OF PAST CLIMATE FROM A 250-KYR ICE-CORE RECORD. *Nature,* 364**,** 218-220.
DANTAS, G., SICILIANO, B., FRANCA, B. B., DA SILVA, C. M. & ARBILLA, G. 2020. The impact of COVID-19 partial lockdown on the air quality of the city of Rio de Janeiro, Brazil. *Science of the Total Environment,* 729**,** 10.
DEE, D. P., UPPALA, S. M., SIMMONS, A., BERRISFORD, P., POLI, P., KOBAYASHI, S., ANDRAE, U., BALMASEDA, M., BALSAMO, G. & BAUER, D. P. 2011. The ERA-Interim reanalysis: Configuration and performance of the data assimilation system. *Quarterly Journal of the royal meteorological society,* 137**,** 553-597.
DEUTSCH, C. A., TEWKSBURY, J. J., HUEY, R. B., SHELDON, K. S., GHALAMBOR, C. K., HAAK, D. C. & MARTIN, P. R. 2008a. Impacts of climate warming on terrestrial ectotherms across latitude. *Proceedings of the National Academy of Sciences,* 105**,** 6668-6672.
DEUTSCH, C. A., TEWKSBURY, J. J., HUEY, R. B., SHELDON, K. S., GHALAMBOR, C. K., HAAK, D. C. & MARTIN, P. R. 2008b. Impacts of climate warming on terrestrial ectotherms across latitude. *Proceedings of the National Academy of Sciences of the United States of America,* 105**,** 6668-6672.
DIAZ, S., SETTELE, J., BRONDIZIO, E. S., NGO, H. T., AGARD, J., ARNETH, A., BALVANERA, P., BRAUMAN, K. A., BUTCHART, S. H. M., CHAN, K. M. A., GARIBALDI, L. A., ICHII, K., LIU, J. G., SUBRAMANIAN, S. M., MIDGLEY, G. F., MILOSLAVICH, P., MOLNAR, Z., OBURA, D., PFAFF, A., POLASKY, S., PURVIS, A., RAZZAQUE, J., REYERS, B., CHOWDHURY, R. R., SHIN, Y. J., VISSEREN-HAMAKERS, I., WILLIS, K. J. & ZAYAS, C. N. 2019. Pervasive human-driven decline of life on Earth points to the need for transformative change. *Science,* 366**,** 1327-+.
DIDHAM, R. K., BASSET, Y., COLLINS, C. M., LEATHER, S. R., LITTLEWOOD, N. A., MENZ, M. H. M., MUELLER, J., PACKER, L., SAUNDERS, M. E., SCHONROGGE, K., STEWART, A. J. A., YANOVIAK, S. P. & HASSALL, C. 2020. Interpreting insect declines: seven challenges and a way forward. *Insect. Conserv. Divers.,* 13**,** 103-114.
DIXON, R. K., BROWN, S., HOUGHTON, R. A., SOLOMON, A. M., TREXLER, M. C. & WISNIEWSKI, J. 1994. CARBON POOLS AND FLUX OF GLOBAL FOREST ECOSYSTEMS. *Science,* 263**,** 185-190.
ELITH, J. & LEATHWICK, J. R. 2009. Species distribution models: ecological explanation and prediction across space and time. *Annual review of ecology, evolution, and systematics,* 40**,** 677-697.
ELITH, J., PHILLIPS, S. J., HASTIE, T., DUDÍK, M., CHEE, Y. E. & YATES, C. J. 2011. A statistical explanation of MaxEnt for ecologists. *Diversity and distributions,* 17**,** 43-57.
ELITH*, J., H. GRAHAM*, C., P. ANDERSON, R., DUDÍK, M., FERRIER, S., GUISAN, A., J. HIJMANS, R., HUETTMANN, F., R. LEATHWICK, J. & LEHMANN, A. 2006. Novel methods improve prediction of species' distributions from occurrence data. *Ecography,* 29**,** 129-151.
ELMER, T., MEPHAM, K. & STADTFELD, C. 2020. Students under lockdown: Comparisons of students' social networks and mental health before and during the COVID-19 crisis in Switzerland. *PLoS One,* 15**,** 22.

EMANUEL, K. 2005. Increasing destructiveness of tropical cyclones over the past 30 years. *Nature,* 436**,** 686-688.
EYRING, V., BONY, S., MEEHL, G. A., SENIOR, C. A., STEVENS, B., STOUFFER, R. J. & TAYLOR, K. E. 2016. Overview of the Coupled Model Intercomparison Project Phase 6 (CMIP6) experimental design and organization. *Geoscientific Model Development,* 9**,** 1937-1958.
FADARE, O. O. & OKOFFO, E. D. 2020. Covid-19 face masks: A potential source of microplastic fibers in the environment. *Science of the Total Environment,* 737**,** 4.
FARGIONE, J., HILL, J., TILMAN, D., POLASKY, S. & HAWTHORNE, P. 2008. Land clearing and the biofuel carbon debt. *Science,* 319**,** 1235-1238.
FOLEY, J. A., DEFRIES, R., ASNER, G. P., BARFORD, C., BONAN, G., CARPENTER, S. R., CHAPIN, F. S., COE, M. T., DAILY, G. C., GIBBS, H. K., HELKOWSKI, J. H., HOLLOWAY, T., HOWARD, E. A., KUCHARIK, C. J., MONFREDA, C., PATZ, J. A., PRENTICE, I. C., RAMANKUTTY, N. & SNYDER, P. K. 2005. Global consequences of land use. *Science,* 309**,** 570-574.
FOLEY, J. A., RAMANKUTTY, N., BRAUMAN, K. A., CASSIDY, E. S., GERBER, J. S., JOHNSTON, M., MUELLER, N. D., O'CONNELL, C., RAY, D. K., WEST, P. C., BALZER, C., BENNETT, E. M., CARPENTER, S. R., HILL, J., MONFREDA, C., POLASKY, S., ROCKSTROM, J., SHEEHAN, J., SIEBERT, S., TILMAN, D. & ZAKS, D. P. M. 2011. Solutions for a cultivated planet. *Nature,* 478**,** 337-342.
FORSTER, P. M., FORSTER, H. I., EVANS, M. J., GIDDEN, M. J., JONES, C. D., KELLER, C. A., LAMBOLL, R. D., LE QUERE, C., ROGELJ, J., ROSEN, D., SCHLEUSSNER, C. F., RICHARDSON, T. B., SMITH, C. J. & TURNOCK, S. T. 2020. Current and future global climate impacts resulting from COVID-19. *Nat. Clim. Chang.,* 10**,** 913-+.
FRIEDLINGSTEIN, P., O'SULLIVAN, M., JONES, M. W., ANDREW, R. M., HAUCK, J., OLSEN, A., PETERS, G. P., PETERS, W., PONGRATZ, J., SITCH, S., LE QUERE, C., CANADELL, J. G., CIAIS, P., JACKSON, R. B., ALIN, S., ARAGAO, L., ARNETH, A., ARORA, V., BATES, N. R., BECKER, M., BENOIT-CATTIN, A., BITTIG, H. C., BOPP, L., BULTAN, S., CHANDRA, N., CHEVALLIER, F., CHINI, L. P., EVANS, W., FLORENTIE, L., FORSTER, P. M., GASSER, T., GEHLEN, M., GILFILLAN, D., GKRITZALIS, T., GREGOR, L., GRUBER, N., HARRIS, I., HARTUNG, K., HAVERD, V., HOUGHTON, R. A., ILYINA, T., JAIN, A. K., JOETZJER, E., KADONO, K., KATO, E., KITIDIS, V., KORSBAKKEN, J. I., LANDSCHUTZER, P., LEFEVRE, N., LENTON, A., LIENERT, S., LIU, Z., LOMBARDOZZI, D., MARLAND, G., METZL, N., MUNRO, D. R., NABEL, J., NAKAOKA, S. I., NIWA, Y., O'BRIEN, K., ONO, T., PALMER, P. I., PIERROT, D., POULTER, B., RESPLANDY, L., ROBERTSON, E., RODENBECK, C., SCHWINGER, J., SEFERIAN, R., SKJELVAN, I., SMITH, A. J. P., SUTTON, A. J., TANHUA, T., TANS, P. P., TIAN, H., TILBROOK, B., VAN DER WERF, G., VUICHARD, N., WALKER, A. P., WANNINKHOF, R., WATSON, A. J., WILLIS, D., WILTSHIRE, A. J., YUAN, W. P., YUE, X. & ZAEHLE, S. 2020. Global Carbon Budget 2020. *Earth Syst. Sci. Data,* 12**,** 3269-3340.
GALLOWAY, J. N., TOWNSEND, A. R., ERISMAN, J. W., BEKUNDA, M., CAI, Z. C., FRENEY, J. R., MARTINELLI, L. A., SEITZINGER, S. P. & SUTTON, M. A. 2008. Transformation of the nitrogen cycle: Recent trends, questions, and potential solutions. *Science,* 320**,** 889-892.
GOODFELLOW, I., BENGIO, Y. & COURVILLE, A. 2016. *Deep learning*, MIT press.
GORHAM, E. 1991. NORTHERN PEATLANDS - ROLE IN THE CARBON-CYCLE AND PROBABLE RESPONSES TO CLIMATIC WARMING. *Ecol. Appl.,* 1**,** 182-195.
GOSSLING, S., SCOTT, D. & HALL, C. M. 2020. Pandemics, tourism and global change: a rapid assessment of COVID-19. *Journal of Sustainable Tourism,* 29**,** 1-20.
GRIMM, N. B., FAETH, S. H., GOLUBIEWSKI, N. E., REDMAN, C. L., WU, J. G., BAI, X. M. & BRIGGS, J. M. 2008. Global change and the ecology of cities. *Science,* 319**,** 756-760.

GUENTHER, A., HEWITT, C. N., ERICKSON, D., FALL, R., GERON, C., GRAEDEL, T., HARLEY, P., KLINGER, L., LERDAU, M., MCKAY, W. A., PIERCE, T., SCHOLES, B., STEINBRECHER, R., TALLAMRAJU, R., TAYLOR, J. & ZIMMERMAN, P. 1995. A GLOBAL-MODEL OF NATURAL VOLATILE ORGANIC-COMPOUND EMISSIONS. *J. Geophys. Res.-Atmos.,* 100**,** 8873-8892.

GURSOY, D. & CHI, C. G. 2020. Effects of COVID-19 pandemic on hospitality industry: review of the current situations and a research agenda. *J. Hosp. Market. Manag.,* 29**,** 527-529.

HAILE, G. G., TANG, Q. H., HOSSEINI-MOGHARI, S. M., LIU, X. C., GEBREMICAEL, T. G., LENG, G. Y., KEBEDE, A., XU, X. M. & YUN, X. B. 2020. Projected Impacts of Climate Change on Drought Patterns Over East Africa. *Earth Future,* 8**,** 23.

HALL, C. M., SCOTT, D. & GOSSLING, S. 2020. Pandemics, transformations and tourism: be careful what you wish for. *Tour. Geogr.,* 22**,** 577-598.

HANSEN, M. C., POTAPOV, P. V., MOORE, R., HANCHER, M., TURUBANOVA, S. A., TYUKAVINA, A., THAU, D., STEHMAN, S. V., GOETZ, S. J., LOVELAND, T. R., KOMMAREDDY, A., EGOROV, A., CHINI, L., JUSTICE, C. O. & TOWNSHEND, J. R. G. 2013. High-Resolution Global Maps of 21st-Century Forest Cover Change. *Science,* 342**,** 850-853.

HARRIGAN, S., ZSOTER, E., ALFIERI, L., PRUDHOMME, C., SALAMON, P., WETTERHALL, F., BARNARD, C., CLOKE, H. & PAPPENBERGER, F. 2020. GloFAS-ERA5 operational global river discharge reanalysis 1979-present. *Earth Syst. Sci. Data,* 12**,** 2043-2060.

HARRIS, I., JONES, P. D., OSBORN, T. J. & LISTER, D. H. 2014. Updated high-resolution grids of monthly climatic observations - the CRU TS3.10 Dataset. *Int. J. Climatol.,* 34**,** 623-642.

HARRIS, I., OSBORN, T. J., JONES, P. & LISTER, D. 2020. Version 4 of the CRU TS monthly high-resolution gridded multivariate climate dataset. *Scientific Data,* 7**,** 18.

HELD, I. M. & SODEN, B. J. 2006. Robust responses of the hydrological cycle to global warming. *Journal of Climate,* 19**,** 5686-5699.

HERSBACH, H., BELL, B., BERRISFORD, P., HIRAHARA, S., HORANYI, A., MUNOZ-SABATER, J., NICOLAS, J., PEUBEY, C., RADU, R., SCHEPERS, D., SIMMONS, A., SOCI, C., ABDALLA, S., ABELLAN, X., BALSAMO, G., BECHTOLD, P., BIAVATI, G., BIDLOT, J., BONAVITA, M., DE CHIARA, G., DAHLGREN, P., DEE, D., DIAMANTAKIS, M., DRAGANI, R., FLEMMING, J., FORBES, R., FUENTES, M., GEER, A., HAIMBERGER, L., HEALY, S., HOGAN, R. J., HOLM, E., JANISKOVA, M., KEELEY, S., LALOYAUX, P., LOPEZ, P., LUPU, C., RADNOTI, G., DE ROSNAY, P., ROZUM, I., VAMBORG, F., VILLAUME, S. & THEPAUT, J. N. 2020. The ERA5 global reanalysis. *Quarterly Journal of the Royal Meteorological Society,* 146**,** 1999-2049.

HIJMANS, R. J., CAMERON, S. E., PARRA, J. L., JONES, P. G. & JARVIS, A. 2005. Very high resolution interpolated climate surfaces for global land areas. *Int. J. Climatol.,* 25**,** 1965-1978.

HINTON, G. E. & SALAKHUTDINOV, R. R. 2006. Reducing the dimensionality of data with neural networks. *Science,* 313**,** 504-507.

HONG, S. Y., NOH, Y. & DUDHIA, J. 2006. A new vertical diffusion package with an explicit treatment of entrainment processes. *Mon. Weather Rev.,* 134**,** 2318-2341.

HOOPER, D. U., CHAPIN, F. S., EWEL, J. J., HECTOR, A., INCHAUSTI, P., LAVOREL, S., LAWTON, J. H., LODGE, D. M., LOREAU, M., NAEEM, S., SCHMID, B., SETALA, H., SYMSTAD, A. J., VANDERMEER, J. & WARDLE, D. A. 2005. Effects of biodiversity on ecosystem functioning: A consensus of current knowledge. *Ecol. Monogr.,* 75**,** 3-35.

HURRELL, J. W. 1995. DECADAL TRENDS IN THE NORTH-ATLANTIC OSCILLATION - REGIONAL TEMPERATURES AND PRECIPITATION. *Science,* 269**,** 676-679.

HURRELL, J. W., HOLLAND, M. M., GENT, P. R., GHAN, S., KAY, J. E., KUSHNER, P. J., LAMARQUE, J.-F., LARGE, W. G., LAWRENCE, D. & LINDSAY, K. 2013. The community earth system model: a framework for collaborative research. *Bull. Amer. Meteorol. Soc.,* 94**,** 1339-1360.
JACOB, D., PETERSEN, J., EGGERT, B., ALIAS, A., CHRISTENSEN, O. B., BOUWER, L. M., BRAUN, A., COLETTE, A., DÉQUÉ, M. & GEORGIEVSKI, G. 2014. EURO-CORDEX: new high-resolution climate change projections for European impact research. *Regional environmental change,* 14**,** 563-578.
JOBBAGY, E. G. & JACKSON, R. B. 2000. The vertical distribution of soil organic carbon and its relation to climate and vegetation. *Ecol. Appl.,* 10**,** 423-436.
JONES, J. W., HOOGENBOOM, G., PORTER, C. H., BOOTE, K. J., BATCHELOR, W. D., HUNT, L. A., WILKENS, P. W., SINGH, U., GIJSMAN, A. J. & RITCHIE, J. T. 2003. The DSSAT cropping system model. *European Journal of Agronomy,* 18**,** 235-265.
KALNAY, E., KANAMITSU, M., KISTLER, R., COLLINS, W., DEAVEN, D., GANDIN, L., IREDELL, M., SAHA, S., WHITE, G., WOOLLEN, J., ZHU, Y., CHELLIAH, M., EBISUZAKI, W., HIGGINS, W., JANOWIAK, J., MO, K. C., ROPELEWSKI, C., WANG, J., LEETMAA, A., REYNOLDS, R., JENNE, R. & JOSEPH, D. 1996. The NCEP/NCAR 40-year reanalysis project. *Bull. Amer. Meteorol. Soc.,* 77**,** 437-471.
KANAMITSU, M., EBISUZAKI, W., WOOLLEN, J., YANG, S.-K., HNILO, J., FIORINO, M. & POTTER, G. 2002. Ncep–doe amip-ii reanalysis (r-2). *Bull. Amer. Meteorol. Soc.,* 83**,** 1631-1644.
KECHNEBBOU, M., DE CARVALHO, D. L., DA SILVA, P. H. & SILVA, D. P. 2021. Global warming drives range shifts in spiny-tailed lizards (Squamata: Agamidae: Uromastyx) in the African and Arabian deserts. *J. Arid. Environ.,* 191**,** 11.
KELLEY, M., SCHMIDT, G. A., NAZARENKO, L. S., BAUER, S. E., RUEDY, R., RUSSELL, G. L., ACKERMAN, A. S., ALEINOV, I., BAUER, M., BLECK, R., CANUTO, V., CESANA, G., CHENG, Y., CLUNE, T. L., COOK, B., CRUZ, C. A., DEL GENIO, A. D., ELSAESSER, G. S., FALUVEGI, G., KIANG, N. Y., KIM, D., LACIS, A. A., LEBOISSETIER, A., LEGRANDE, A. N., LO, K. K., MARSHALL, J., MATTHEWS, E. E., MCDERMID, S., MEZUMAN, K., MILLER, R. L., MURRAY, L. T., OINAS, V., ORBE, C., GARCIA-PANDO, C. P., PERLWITZ, J. P., PUMA, M. J., RIND, D., ROMANOU, A., SHINDELL, D. T., SUN, S., TAUSNEV, N., TSIGARIDIS, K., TSELIOUDIS, G., WENG, E. S., WU, J. B. & YAO, M. S. 2020. GISS-E2.1: Configurations and Climatology. *J. Adv. Model. Earth Syst.,* 12**,** 38.
KOTTEK, M., GRIESER, J., BECK, C., RUDOLF, B. & RUBEL, F. 2006a. World map of the Koppen-Geiger climate classification updated. *Meteorologische Zeitschrift,* 15**,** 259-263.
KOTTEK, M., GRIESER, J., BECK, C., RUDOLF, B. & RUBEL, F. 2006b. World map of the Köppen-Geiger climate classification updated.
LAL, R. 2004. Soil carbon sequestration impacts on global climate change and food security. *Science,* 304**,** 1623-1627.
LARGE, W. G., MCWILLIAMS, J. C. & DONEY, S. C. 1994. OCEANIC VERTICAL MIXING - A REVIEW AND A MODEL WITH A NONLOCAL BOUNDARY-LAYER PARAMETERIZATION. *Rev. Geophys.,* 32**,** 363-403.
LAXMINARAYAN, R., DUSE, A., WATTAL, C., ZAIDI, A. K. M., WERTHEIM, H. F. L., SUMPRADIT, N., VLIEGHE, E., HARA, G. L., GOULD, I. M., GOOSSENS, H., GREKO, C., SO, A. D., BIGDELI, M., TOMSON, G., WOODHOUSE, W., OMBAKA, E., PERALTA, A. Q., QAMAR, F. N., MIR, F., KARIUKI, S., BHUTTA, Z. A., COATES, A., BERGSTROM, R., WRIGHT, G. D., BROWN, E. D. & CARS, O. 2013. Antibiotic resistance-the need for global solutions. *Lancet Infectious Diseases,* 13**,** 1057-1098.
LE QUERE, C., JACKSON, R. B., JONES, M. W., SMITH, A. J. P., ABERNETHY, S., ANDREW, R. M., DE-GOL, A. J., WILLIS, D. R., SHAN, Y. L., CANADELL, O. S., FRIEDLINGSTEIN, P. E. R., CREUTZIG, E. L. & PETERS, E. 2020. Temporary reduction in daily global CO2 emissions during the COVID-19 forced confinement. *Nat. Clim. Chang.,* 10**,** 647-+.

LEGATES, D. R. & MCCABE, G. J. 1999. Evaluating the use of "goodness-of-fit" measures in hydrologic and hydroclimatic model validation. *Water Resources Research,* 35**,** 233-241.

LEHNER, F., DESER, C., MAHER, N., MAROTZKE, J., FISCHER, E. M., BRUNNER, L., KNUTTI, R. & HAWKINS, E. 2020. Partitioning climate projection uncertainty with multiple large ensembles and CMIP5/6. *Earth Syst. Dynam.,* 11**,** 491-508.

LELIEVELD, J., EVANS, J. S., FNAIS, M., GIANNADAKI, D. & POZZER, A. 2015. The contribution of outdoor air pollution sources to premature mortality on a global scale. *Nature,* 525**,** 367-+.

LIAO, Y. H., KOELEWIJN, S. F., VAN DEN BOSSCHE, G., VAN AELST, J., VAN DEN BOSCH, S., RENDERS, T., NAVARE, K., NICOLAI, T., VAN AELST, K., MAESEN, M., MATSUSHIMA, H., THEVELEIN, J. M., VAN ACKER, K., LAGRAIN, B., VERBOEKEND, D. & SELS, B. F. 2020. A sustainable wood biorefinery for low-carbon footprint chemicals production. *Science,* 367**,** 1385-+.

LLOYD, J. & TAYLOR, J. A. 1994. ON THE TEMPERATURE-DEPENDENCE OF SOIL RESPIRATION. *Functional Ecology,* 8**,** 315-323.

LOSADA-BALTAR, A., JIMENEZ-GONZALO, L., GALLEGO-ALBERTO, L., PEDROSO-CHAPARRO, M. D., FERNANDES-PIRES, J. & MARQUEZ-GONZALEZ, M. 2021. "We Are Staying at Home." Association of Self-perceptions of Aging, Personal and Family Resources, and Loneliness With Psychological Distress During the Lock-Down Period of COVID-19. *J. Gerontol. Ser. B-Psychol. Sci. Soc. Sci.,* 76**,** E10-E16.

MAHATO, S., PAL, S. & GHOSH, K. G. 2020. Effect of lockdown amid COVID-19 pandemic on air quality of the megacity Delhi, India. *Science of the Total Environment,* 730**,** 23.

MANENTI, R., MORI, E., DI CANIO, V., MERCURIO, S., PICONE, M., CAFFI, M., BRAMBILLA, M., FICETOLA, G. F. & RUBOLINI, D. 2020. The good, the bad and the ugly of COVID-19 lockdown effects on wildlife conservation: Insights from the first European locked down country. *Biol. Conserv.,* 249**,** 9.

MANTUA, N. J., HARE, S. R., ZHANG, Y., WALLACE, J. M. & FRANCIS, R. C. 1997. A Pacific interdecadal climate oscillation with impacts on salmon production. *Bull. Amer. Meteorol. Soc.,* 78**,** 1069-1079.

MCDOWELL, N. G., ALLEN, C. D., ANDERSON-TEIXEIRA, K., AUKEMA, B. H., BOND-LAMBERTY, B., CHINI, L., CLARK, J. S., DIETZE, M., GROSSIORD, C., HANBURY-BROWN, A., HURTT, G. C., JACKSON, R. B., JOHNSON, D. J., KUEPPERS, L., LICHSTEIN, J. W., OGLE, K., POULTER, B., PUGH, T. A. M., SEIDL, R., TURNER, M. G., URIARTE, M., WALKER, A. P. & XU, C. G. 2020. Pervasive shifts in forest dynamics in a changing world. *Science,* 368**,** 964-+.

MCGILL, B. J., ENQUIST, B. J., WEIHER, E. & WESTOBY, M. 2006. Rebuilding community ecology from functional traits. *Trends Ecol. Evol.,* 21**,** 178-185.

MEEHL, G. A., COVEY, C., DELWORTH, T., LATIF, M., MCAVANEY, B., MITCHELL, J. F. B., STOUFFER, R. J. & TAYLOR, K. E. 2007. The WCRP CMIP3 multimodel dataset - A new era in climate change research. *Bull. Amer. Meteorol. Soc.,* 88**,** 1383-1394.

MEEHL, G. A., SENIOR, C. A., EYRING, V., FLATO, G., LAMARQUE, J. F., STOUFFER, R. J., TAYLOR, K. E. & SCHLUND, M. 2020. Context for interpreting equilibrium climate sensitivity and transient climate response from the CMIP6 Earth system models. *Science Advances,* 6**,** 10.

MEINSHAUSEN, M., SMITH, S. J., CALVIN, K., DANIEL, J. S., KAINUMA, M. L. T., LAMARQUE, J. F., MATSUMOTO, K., MONTZKA, S. A., RAPER, S. C. B., RIAHI, K., THOMSON, A., VELDERS, G. J. M. & VAN VUUREN, D. P. P. 2011. The RCP greenhouse gas concentrations and their extensions from 1765 to 2300. *Climatic Change,* 109**,** 213.

MENZEL, A., SPARKS, T. H., ESTRELLA, N., KOCH, E., AASA, A., AHAS, R., ALM-KUBLER, K., BISSOLLI, P., BRASLAVSKA, O., BRIEDE, A., CHMIELEWSKI, F. M., CREPINSEK, Z., CURNEL, Y., DAHL, A., DEFILA, C., DONNELLY, A., FILELLA, Y., JATCZA, K., MAGE, F., MESTRE, A., NORDLI, O., PENUELAS, J., PIRINEN, P., REMISOVA, V., SCHEIFINGER, H., STRIZ, M., SUSNIK, A., VAN VLIET, A. J. H., WIELGOLASKI, F. E., ZACH, S. & ZUST, A. 2006. European phenological response to climate change matches the warming pattern. *Global Change Biology,* 12**,** 1969-1976.

MESINGER, F., DIMEGO, G., KALNAY, E., MITCHELL, K., SHAFRAN, P. C., EBISUZAKI, W., JOVIC, D., WOOLLEN, J., ROGERS, E., BERBERY, E. H., EK, M. B., FAN, Y., GRUMBINE, R., HIGGINS, W., LI, H., LIN, Y., MANIKIN, G., PARRISH, D. & SHI, W. 2006. North American regional reanalysis. *Bull. Amer. Meteorol. Soc.,* 87**,** 343-360.

MILLIMAN, J. D. & SYVITSKI, J. P. M. 1992. GEOMORPHIC TECTONIC CONTROL OF SEDIMENT DISCHARGE TO THE OCEAN - THE IMPORTANCE OF SMALL MOUNTAINOUS RIVERS. *J. Geol.,* 100**,** 525-544.

MITCHELL, T. D. & JONES, P. D. 2005. An improved method of constructing a database of monthly climate observations and associated high-resolution grids. *Int. J. Climatol.,* 25**,** 693-712.

MLAWER, E. J., TAUBMAN, S. J., BROWN, P. D., IACONO, M. J. & CLOUGH, S. A. 1997. Radiative transfer for inhomogeneous atmospheres: RRTM, a validated correlated-k model for the longwave. *J. Geophys. Res.-Atmos.,* 102**,** 16663-16682.

MONTEITH, J. L. 1977. CLIMATE AND EFFICIENCY OF CROP PRODUCTION IN BRITAIN. *Philos. Trans. R. Soc. Lond. Ser. B-Biol. Sci.,* 281**,** 277-294.

MOSS, R. H., EDMONDS, J. A., HIBBARD, K. A., MANNING, M. R., ROSE, S. K., VAN VUUREN, D. P., CARTER, T. R., EMORI, S., KAINUMA, M., KRAM, T., MEEHL, G. A., MITCHELL, J. F. B., NAKICENOVIC, N., RIAHI, K., SMITH, S. J., STOUFFER, R. J., THOMSON, A. M., WEYANT, J. P. & WILBANKS, T. J. 2010. The next generation of scenarios for climate change research and assessment. *Nature,* 463**,** 747-756.

NAGHAVI, M., ABAJOBIR, A. A., ABBAFATI, C., ABBAS, K. M., ABD-ALLAH, F., ABERA, S. F., ABOYANS, V., ADETOKUNBOH, O., ARNLOV, J., AFSHIN, A., AGRAWAL, A., KIADALIRI, A. A., AHMADI, A., AHMED, M. B., AICHOUR, A. N., AICHOUR, I., AICHOUR, M. T. E., AIYAR, S., AL-EYADHY, A., ALAHDAB, F., AL-ALY, Z., ALAM, K., ALAM, N., ALAM, T., ALENE, K. A., ALI, S. D., ALIZADEH-NAVAEI, R., ALKAABI, J. M., ALKERWI, A., ALLA, F., ALLEBECK, P., ALLEN, C., AL-RADDADI, R., ALSHARIF, U., ALTIRKAWI, K. A., ALVIS-GUZMAN, N., AMARE, A. T., AMINI, E., AMMAR, W., AMOAKO, Y. A., ANBER, N., ANDERSEN, H. H., ANDREI, C. L., ANDROUDI, S., ANSARI, H., ANTONIO, C. A. T., ANWARI, P., ARORA, M., ARTAMAN, A., ARYAL, K. K., ASAYESH, H., ASGEDOM, S. W., ATEY, T. M., AVILA-BURGOS, L., AVOKPAHO, E., AWASTHI, A., PAULINA, B., QUINTANILLA, A., BEJOT, Y., BABALOLA, T. K., BACHA, U., BALAKRISHNAN, K., BARAC, A., BARBOZA, M. A., BARKER-COLLO, S. L., BARQUERA, S., BARREGARD, L., BARRERO, L. H., BAUNE, B. T., BEDI, N., BEGHI, E., BEKELE, B. B., BELL, M. L., BENNETT, J. R., BENSENOR, I. M., BERHANE, A., BERNABE, E., BETSU, B. D., BEURAN, M., BHATT, S., BIADGILIGN, S., BIENHOFF, K., BIKBOV, B., BISANZIO, D., BOURNE, R. R. A., BREITBORDE, N. J. K., NEGESA, L., BULTO, B., BUMGARNER, B. R., BUTT, Z. A., CARDENAS, R., CAHUANA-HURTADO, L., CAMERON, E., CAMPUZANO, J. C., CAR, J., CARRERO, J. J., CARTER, A., CASEY, D. C., CASTANEDA-ORJUELA, C. A., CATALA-LOPEZ, F., et al. 2017. Global, regional, and national age-sex specific mortality for 264 causes of death, 1980-2016: a systematic analysis for the Global Burden of Disease Study 2016. *Lancet,* 390**,** 1151-1210.

NOTZ, D., DORR, J., BAILEY, D. A., BLOCKLEY, E., BUSHUK, M., DEBERNARD, J. B., DEKKER, E., DEREPENTIGNY, P., DOCQUIER, D., FUCKAR, N. S., FYFE, J. C., JAHN, A., HOLLAND, M., HUNKE, E., IOVINO, D., KHOSRAVI, N., MADEC, G., MASSONNET, F., O'FARRELL, S., PETTY, A., RANA, A., ROACH, L., ROSENBLUM, E., ROUSSET, C., SEMMLER, T., STROEVE, J., TOYODA, T., TREMBLAY, B., TSUJINO, H., VANCOPPENOLLE, M. & COMMUNITY, S. 2020. Arctic Sea Ice in CMIP6. *Geophysical Research Letters,* 47**,** 11.

ORDONEZ, C., GARRIDO-PEREZ, J. M. & GARCIA-HERRERA, R. 2020. Early spring near-surface ozone in Europe during the COVID-19 shutdown: Meteorological effects outweigh emission changes. *Science of the Total Environment,* 747**,** 10.

OUTHWAITE, C. L., GREGORY, R. D., CHANDLER, R. E., COLLEN, B. & ISAAC, N. J. B. 2020. Complex long-term biodiversity change among invertebrates, bryophytes and lichens. *Nat. Ecol. Evol.,* 4**,** 384-+.

PARMESAN, C. 2006. Ecological and evolutionary responses to recent climate change. *Annu. Rev. Ecol. Evol. Syst.,* 37**,** 637-669.

PARMESAN, C. & YOHE, G. 2003. A globally coherent fingerprint of climate change impacts across natural systems. *Nature,* 421**,** 37-42.

PEARSON, R. G., RAXWORTHY, C. J., NAKAMURA, M. & TOWNSEND PETERSON, A. 2007. Predicting species distributions from small numbers of occurrence records: a test case using cryptic geckos in Madagascar. *Journal of biogeography,* 34**,** 102-117.

PEEL, M. C., FINLAYSON, B. L. & MCMAHON, T. A. 2007a. Updated world map of the Koppen-Geiger climate classification. *Hydrol. Earth Syst. Sci.,* 11**,** 1633-1644.

PEEL, M. C., FINLAYSON, B. L. & MCMAHON, T. A. 2007b. Updated world map of the Köppen-Geiger climate classification. *Hydrol. Earth Syst. Sci.,* 11**,** 1633-1644.

PEREZ-LOMBARD, L., ORTIZ, J. & POUT, C. 2008. A review on buildings energy consumption information. *Energy and Buildings,* 40**,** 394-398.

PERRY, A. L., LOW, P. J., ELLIS, J. R. & REYNOLDS, J. D. 2005. Climate change and distribution shifts in marine fishes. *Science,* 308**,** 1912-1915.

PETETIN, H., BOWDALO, D., SORET, A., GUEVARA, M., JORBA, O., SERRADELL, K. & GARCIA-PANDO, C. P. 2020. Meteorology-normalized impact of the COVID-19 lockdown upon NO2 pollution in Spain. *Atmos. Chem. Phys.,* 20**,** 11119-11141.

PETIT, J. R., JOUZEL, J., RAYNAUD, D., BARKOV, N. I., BARNOLA, J. M., BASILE, I., BENDER, M., CHAPPELLAZ, J., DAVIS, M., DELAYGUE, G., DELMOTTE, M., KOTLYAKOV, V. M., LEGRAND, M., LIPENKOV, V. Y., LORIUS, C., PEPIN, L., RITZ, C., SALTZMAN, E. & STIEVENARD, M. 1999. Climate and atmospheric history of the past 420,000 years from the Vostok ice core, Antarctica. *Nature,* 399**,** 429-436.

PHILLIPS, S. J., ANDERSON, R. P. & SCHAPIRE, R. E. 2006. Maximum entropy modeling of species geographic distributions. *Ecol. Model.,* 190**,** 231-259.

PHILLIPS, S. J. & DUDÍK, M. 2008. Modeling of species distributions with Maxent: new extensions and a comprehensive evaluation. *Ecography,* 31**,** 161-175.

PIAO, S. L., WANG, X. H., PARK, T., CHEN, C., LIAN, X., HE, Y., BJERKE, J. W., CHEN, A. P., CIAIS, P., TOMMERVIK, H., NEMANI, R. R. & MYNENI, R. B. 2020. Characteristics, drivers and feedbacks of global greening. *Nat. Rev. Earth Environ.,* 1**,** 14-27.

RAICH, J. W. & SCHLESINGER, W. H. 1992. THE GLOBAL CARBON-DIOXIDE FLUX IN SOIL RESPIRATION AND ITS RELATIONSHIP TO VEGETATION AND CLIMATE. *Tellus Series B-Chemical and Physical Meteorology,* 44**,** 81-99.

RAMANATHAN, V., CRUTZEN, P. J., KIEHL, J. T. & ROSENFELD, D. 2001. Atmosphere - Aerosols, climate, and the hydrological cycle. *Science,* 294**,** 2119-2124.

RAVISHANKARA, A. R., DANIEL, J. S. & PORTMANN, R. W. 2009. Nitrous Oxide (N2O): The Dominant Ozone-Depleting Substance Emitted in the 21st Century. *Science,* 326**,** 123-125.

RAYNER, N. A., PARKER, D. E., HORTON, E. B., FOLLAND, C. K., ALEXANDER, L. V., ROWELL, D. P., KENT, E. C. & KAPLAN, A. 2003a. Global analyses of sea surface temperature, sea ice, and night marine air temperature since the late nineteenth century. *J. Geophys. Res.-Atmos.,* 108.

RAYNER, N. A., PARKER, D. E., HORTON, E. B., FOLLAND, C. K., ALEXANDER, L. V., ROWELL, D. P., KENT, E. C. & KAPLAN, A. 2003b. Global analyses of sea surface temperature, sea ice, and night marine air temperature since the late nineteenth century. *J. Geophys. Res.-Atmos.,* 108**,** 37.

REID, A. J., CARLSON, A. K., CREED, I. F., ELIASON, E. J., GELL, P. A., JOHNSON, P. T. J., KIDD, K. A., MACCORMACK, T. J., OLDEN, J. D., ORMEROD, S. J., SMOL, J. P., TAYLOR, W. W., TOCKNER, K., VERMAIRE, J. C., DUDGEON, D. & COOKE, S. J. 2019. Emerging threats and persistent conservation challenges for freshwater biodiversity. *Biological Reviews,* 94**,** 849-873.

REYNOLDS, R. W., RAYNER, N. A., SMITH, T. M., STOKES, D. C. & WANG, W. Q. 2002. An improved in situ and satellite SST analysis for climate. *Journal of Climate,* 15**,** 1609-1625.

REYNOLDS, R. W., SMITH, T. M., LIU, C., CHELTON, D. B., CASEY, K. S. & SCHLAX, M. G. 2007. Daily high-resolution-blended analyses for sea surface temperature. *J. Clim.,* 20**,** 5473-5496.

RIENECKER, M. M., SUAREZ, M. J., GELARO, R., TODLING, R., BACMEISTER, J., LIU, E., BOSILOVICH, M. G., SCHUBERT, S. D., TAKACS, L., KIM, G. K., BLOOM, S., CHEN, J. Y., COLLINS, D., CONATY, A., DA SILVA, A., GU, W., JOINER, J., KOSTER, R. D., LUCCHESI, R., MOLOD, A., OWENS, T., PAWSON, S., PEGION, P., REDDER, C. R., REICHLE, R., ROBERTSON, F. R., RUDDICK, A. G., SIENKIEWICZ, M. & WOOLLEN, J. 2011. MERRA: NASA's Modern-Era Retrospective Analysis for Research and Applications. *J. Clim.,* 24**,** 3624-3648.

ROCKSTROM, J., STEFFEN, W., NOONE, K., PERSSON, A., CHAPIN, F. S., LAMBIN, E. F., LENTON, T. M., SCHEFFER, M., FOLKE, C., SCHELLNHUBER, H. J., NYKVIST, B., DE WIT, C. A., HUGHES, T., VAN DER LEEUW, S., RODHE, H., SORLIN, S., SNYDER, P. K., COSTANZA, R., SVEDIN, U., FALKENMARK, M., KARLBERG, L., CORELL, R. W., FABRY, V. J., HANSEN, J., WALKER, B., LIVERMAN, D., RICHARDSON, K., CRUTZEN, P. & FOLEY, J. A. 2009. A safe operating space for humanity. *Nature,* 461**,** 472-475.

ROOT, T. L., PRICE, J. T., HALL, K. R., SCHNEIDER, S. H., ROSENZWEIG, C. & POUNDS, J. A. 2003. Fingerprints of global warming on wild animals and plants. *Nature,* 421**,** 57-60.

RUBEL, F. & KOTTEK, M. 2010. Observed and projected climate shifts 1901-2100 depicted by world maps of the Koppen-Geiger climate classification. *Meteorol. Z.,* 19**,** 135-141.

SAHA, S., MOORTHI, S., PAN, H. L., WU, X. R., WANG, J. D., NADIGA, S., TRIPP, P., KISTLER, R., WOOLLEN, J., BEHRINGER, D., LIU, H. X., STOKES, D., GRUMBINE, R., GAYNO, G., WANG, J., HOU, Y. T., CHUANG, H. Y., JUANG, H. M. H., SELA, J., IREDELL, M., TREADON, R., KLEIST, D., VAN DELST, P., KEYSER, D., DERBER, J., EK, M., MENG, J., WEI, H. L., YANG, R. Q., LORD, S., VAN DEN DOOL, H., KUMAR, A., WANG, W. Q., LONG, C., CHELLIAH, M., XUE, Y., HUANG, B. Y., SCHEMM, J. K., EBISUZAKI, W., LIN, R., XIE, P. P., CHEN, M. Y., ZHOU, S. T., HIGGINS, W., ZOU, C. Z., LIU, Q. H., CHEN, Y., HAN, Y., CUCURULL, L., REYNOLDS, R. W., RUTLEDGE, G. & GOLDBERG, M. 2010. THE NCEP CLIMATE FORECAST SYSTEM REANALYSIS. *Bull. Amer. Meteorol. Soc.,* 91**,** 1015-1057.

SAJI, N. H., GOSWAMI, B. N., VINAYACHANDRAN, P. N. & YAMAGATA, T. 1999. A dipole mode in the tropical Indian Ocean. *Nature,* 401**,** 360-363.

SCHAR, C., VIDALE, P. L., LUTHI, D., FREI, C., HABERLI, C., LINIGER, M. A. & APPENZELLER, C. 2004. The role of increasing temperature variability in European summer heatwaves. *Nature,* 427**,** 332-336.

SCHEFFER, M., CARPENTER, S., FOLEY, J. A., FOLKE, C. & WALKER, B. 2001. Catastrophic shifts in ecosystems. *Nature,* 413**,** 591-596.

SCHMIDT, M. W. I., TORN, M. S., ABIVEN, S., DITTMAR, T., GUGGENBERGER, G., JANSSENS, I. A., KLEBER, M., KOGEL-KNABNER, I., LEHMANN, J., MANNING, D. A. C., NANNIPIERI, P., RASSE, D. P., WEINER, S. & TRUMBORE, S. E. 2011. Persistence of soil organic matter as an ecosystem property. *Nature,* 478**,** 49-56.

SEIBOLD, S., GOSSNER, M. M., SIMONS, N. K., BLUTHGEN, N., MULLER, J., AMBARLI, D., AMMER, C., BAUHUS, J., FISCHER, M., HABEL, J. C., LINSENMAIR, K. E., NAUSS, T., PENONE, C., PRATI, D., SCHALL, P., SCHULZE, E. D., VOGT, J., WOLLAUER, S. & WEISSER, W. W. 2019. Arthropod decline in grasslands and forests is associated with landscape-level drivers. *Nature,* 574**,** 671-+.

SHARIFI, A. & KHAVARIAN-GARMSIR, A. R. 2020. The COVID-19 pandemic: Impacts on cities and major lessons for urban planning, design, and management. *Science of the Total Environment,* 749**,** 14.

SHERWOOD, S. C., WEBB, M. J., ANNAN, J. D., ARMOUR, K. C., FORSTER, P. M., HARGREAVES, J. C., HEGERL, G., KLEIN, S. A., MARVEL, K. D., ROHLING, E. J., WATANABE, M., ANDREWS, T., BRACONNOT, P., BRETHERTON, C. S., FOSTER, G. L., HAUSFATHER, Z., HEYDT, A. S., KNUTTI, R., MAURITSEN, T., NORRIS, J. R., PROISTOSESCU, C., RUGENSTEIN, M., SCHMIDT, G. A., TOKARSKA, K. B. & ZELINKA, M. D. 2020. An Assessment of Earth's Climate Sensitivity Using Multiple Lines of Evidence. *Rev. Geophys.,* 58**,** 92.

SIBLEY, C. G., GREAVES, L. M., SATHERLEY, N., WILSON, M. S., OVERALL, N. C., LEE, C. H. J., MILOJEV, P., BULBULIA, J., OSBORNE, D., MILFONT, T. L., HOUKAMAU, C. A., DUCK, I. M., VICKERS-JONES, R. & BARLOW, F. K. 2020. Effects of the COVID-19 Pandemic and Nationwide Lockdown on Trust, Attitudes Toward Government, and Well-Being. *Am. Psychol.,* 75**,** 618-630.

SICARD, P., DE MARCO, A., AGATHOKLEOUS, E., FENG, Z. Z., XU, X. B., PAOLETTI, E., RODRIGUEZ, J. J. D. & CALATAYUD, V. 2020. Ampli fied ozone pollution in cities during the COVID-19 lockdown. *Science of the Total Environment,* 735**,** 10.

SIGALA, M. 2020. Tourism and COVID-19: Impacts and implications for advancing and resetting industry and research. *J. Bus. Res.,* 117**,** 312-321.

SMIT, B. & WANDEL, J. 2006. Adaptation, adaptive capacity and vulnerability. *Global Environmental Change-Human and Policy Dimensions,* 16**,** 282-292.

SMITH, T. M., REYNOLDS, R. W., PETERSON, T. C. & LAWRIMORE, J. 2008a. Improvements to NOAA's historical merged land-ocean surface temperature analysis (1880-2006). *J. Clim.,* 21**,** 2283-2296.

SMITH, T. M., REYNOLDS, R. W., PETERSON, T. C. & LAWRIMORE, J. 2008b. Improvements to NOAA’s historical merged land–ocean surface temperature analysis (1880–2006). *J. Clim.,* 21**,** 2283-2296.

SRIVASTAVA, J., MANJUNATHA, B. R., BALAKRISHNA, K., PRAJITH, A., MANJUNATHA, H. V., JOSE, J. & KUMAR, N. 2021. Quantitative pollen-based reconstruction of the vegetation diversity in response to the late-Holocene climate change near Karwar, south-west coast of India. *Quat. Int.,* 599**,** 95-106.

STEFFEN, W., RICHARDSON, K., ROCKSTROM, J., CORNELL, S. E., FETZER, I., BENNETT, E. M., BIGGS, R., CARPENTER, S. R., DE VRIES, W., DE WIT, C. A., FOLKE, C., GERTEN, D., HEINKE, J., MACE, G. M., PERSSON, L. M., RAMANATHAN, V., REYERS, B. & SORLIN, S. 2015. Planetary boundaries: Guiding human development on a changing planet. *Science,* 347.

STUART, S. N., CHANSON, J. S., COX, N. A., YOUNG, B. E., RODRIGUES, A. S. L., FISCHMAN, D. L. & WALLER, R. W. 2004. Status and trends of amphibian declines and extinctions worldwide. *Science,* 306**,** 1783-1786.

TAYLOR, K. E., STOUFFER, R. J. & MEEHL, G. A. 2012. An overview of CMIP5 and the experiment design. *Bull. Amer. Meteorol. Soc.,* 93**,** 485-498.

TENENBAUM, J. B., DE SILVA, V. & LANGFORD, J. C. 2000a. A global geometric framework for nonlinear dimensionality reduction. *Science,* 290**,** 2319-+.

TENENBAUM, J. B., DE SILVA, V. & LANGFORD, J. C. 2000b. A global geometric framework for nonlinear dimensionality reduction. *Science,* 290**,** 2319-2323.

THOMAS, C. D., CAMERON, A., GREEN, R. E., BAKKENES, M., BEAUMONT, L. J., COLLINGHAM, Y. C., ERASMUS, B. F., DE SIQUEIRA, M. F., GRAINGER, A. & HANNAH, L. 2004a. Extinction risk from climate change. *Nature,* 427**,** 145-148.

THOMAS, C. D., CAMERON, A., GREEN, R. E., BAKKENES, M., BEAUMONT, L. J., COLLINGHAM, Y. C., ERASMUS, B. F. N., DE SIQUEIRA, M. F., GRAINGER, A., HANNAH, L., HUGHES, L., HUNTLEY, B., VAN JAARSVELD, A. S., MIDGLEY, G. F., MILES, L., ORTEGA-HUERTA, M. A., PETERSON, A. T., PHILLIPS, O. L. & WILLIAMS, S. E. 2004b. Extinction risk from climate change. *Nature,* 427**,** 145-148.

THURNER, S. D., CONVERSE, S. J. & BRANCH, T. A. 2021. Modeling opportunistic exploitation: increased extinction risk when targeting more than one species. *Ecol. Model.,* 454**,** 12.

TICKNER, D., OPPERMAN, J. J., ABELL, R., ACREMAN, M., ARTHINGTON, A. H., BUNN, S. E., COOKE, S. J., DALTON, J., DARWALL, W., EDWARDS, G., HARRISON, I., HUGHES, K., JONES, T., LECLERE, D., LYNCH, A. J., LEONARD, P., MCCLAIN, M. E., MURUVEN, D., OLDEN, J. D., ORMEROD, S. J., ROBINSON, J., THARME, R. E., THIEME, M., TOCKNER, K., WRIGHT, M. & YOUNG, L. 2020. Bending the Curve of Global Freshwater Biodiversity Loss: An Emergency Recovery Plan. *Bioscience,* 70**,** 330-342.

TILMAN, D., BALZER, C., HILL, J. & BEFORT, B. L. 2011. Global food demand and the sustainable intensification of agriculture. *Proceedings of the National Academy of Sciences of the United States of America,* 108**,** 20260-20264.

TOBIAS, A., CARNERERO, C., RECHE, C., MASSAGUE, J., VIA, M., MINGUILLON, M. C., ALASTUEY, A. & QUEROL, X. 2020. Changes in air quality during the lockdown in Barcelona (Spain) one month into the SARS-CoV-2 epidemic. *Science of the Total Environment,* 726**,** 4.

TRENBERTH, K. E. & HURRELL, J. W. 1994. DECADAL ATMOSPHERE-OCEAN VARIATIONS IN THE PACIFIC. *Climate Dynamics,* 9**,** 303-319.

TURNER, B. L., KASPERSON, R. E., MATSON, P. A., MCCARTHY, J. J., CORELL, R. W., CHRISTENSEN, L., ECKLEY, N., KASPERSON, J. X., LUERS, A., MARTELLO, M. L., POLSKY, C., PULSIPHER, A. & SCHILLER, A. 2003. A framework for vulnerability analysis in sustainability science. *Proceedings of the National Academy of Sciences of the United States of America,* 100**,** 8074-8079.

UPPALA, S. M., KÅLLBERG, P., SIMMONS, A., ANDRAE, U., BECHTOLD, V. D. C., FIORINO, M., GIBSON, J., HASELER, J., HERNANDEZ, A. & KELLY, G. 2005a. The ERA-40 re-analysis. *Quarterly Journal of the Royal Meteorological Society: A journal of the atmospheric sciences, applied meteorology and physical oceanography,* 131**,** 2961-3012.

UPPALA, S. M., KALLBERG, P. W., SIMMONS, A. J., ANDRAE, U., BECHTOLD, V. D., FIORINO, M., GIBSON, J. K., HASELER, J., HERNANDEZ, A., KELLY, G. A., LI, X., ONOGI, K., SAARINEN, S., SOKKA, N., ALLAN, R. P., ANDERSSON, E., ARPE, K., BALMASEDA, M. A., BELJAARS, A. C. M., VAN DE BERG, L., BIDLOT, J., BORMANN, N., CAIRES, S., CHEVALLIER, F., DETHOF, A., DRAGOSAVAC, M., FISHER, M., FUENTES, M., HAGEMANN, S., HOLM, E., HOSKINS, B. J., ISAKSEN, L., JANSSEN, P., JENNE, R., MCNALLY, A. P., MAHFOUF, J. F., MORCRETTE, J. J., RAYNER, N. A., SAUNDERS, R. W., SIMON, P., STERL, A., TRENBERTH, K. E., UNTCH, A., VASILJEVIC, D., VITERBO, P. & WOOLLEN, J. 2005b. The ERA-40 re-analysis. *Quarterly Journal of the Royal Meteorological Society,* 131**,** 2961-3012.

VAN BAVEL, J. J., BAICKER, K., BOGGIO, P. S., CAPRARO, V., CICHOCKA, A., CIKARA, M., CROCKETT, M. J., CRUM, A. J., DOUGLAS, K. M., DRUCKMAN, J. N., DRURY, J., DUBE, O., ELLEMERS, N., FINKEL, E. J., FOWLER, J. H., GELFAND, M., HAN, S. H., HASLAM, S. A., JETTEN, J., KITAYAMA, S., MOBBS, D., NAPPER, L. E., PACKER, D. J., PENNYCOOK, G., PETERS, E., PETTY, R. E., RAND, D. G., REICHER, S. D., SCHNALL, S., SHARIFF, A., SKITKA, L. J., SMITH, S. S., SUNSTEIN, C. R., TABRI, N., TUCKER, J. A., VAN DER LINDEN, S., VAN LANGE, P., WEEDEN, K. A., WOHL, M. J. A., ZAKI, J., ZION, S. R. & WILLER, R. 2020. Using social and behavioural science to support COVID-19 pandemic response. *Nat. Hum. Behav.,* 4**,** 460-471.

VAN DER MAATEN, L. & HINTON, G. 2008. Visualizing data using t-SNE. *Journal of machine learning research,* 9.

VELDMAN, J. W., ALEMAN, J. C., ALVARADO, S. T., ANDERSON, T. M., ARCHIBALD, S., BOND, W. J., BOUTTON, T. W., BUCHMANN, N., BUISSON, E., CANADELL, J. G., DECHOUM, M. D., DIAZ-TORIBIO, M. H., DURIGAN, G., EWEL, J. J., FERNANDES, G. W., FIDELIS, A., FLEISCHMAN, F., GOOD, S. P., GRIFFITH, D. M., HERMANN, J. M., HOFFMANN, W. A., STRADIC, S., LEHMANN, C. E. R., MAHY, G., NERLEKAR, A. N., NIPPERT, J. B., NOSS, R. F., OSBORNE, C. P., OVERBECK, G. E., PARR, C. L., PAUSAS, J. G., PENNINGTON, R. T., PERRING, M. P., PUTZ, F. E., RATNAM, J., SANKARAN, M., SCHMIDT, I. B., SCHMITT, C. B., SILVEIRA, F. A. O., STAVER, A. C., STEVENS, N., STILL, C. J., STROMBERG, C. A. E., TEMPERTON, V. M., VARNER, J. M. & ZALOUMIS, N. P. 2019. Comment on "The global tree restoration potential". *Science,* 366**,** 4.

VENTER, Z. S., AUNAN, K., CHOWDHURY, S. & LELIEVELD, J. 2020. COVID-19 lockdowns cause global air pollution declines. *Proceedings of the National Academy of Sciences of the United States of America,* 117**,** 18984-18990.

VICENTE-SERRANO, S. M., BEGUERIA, S. & LOPEZ-MORENO, J. I. 2010. A Multiscalar Drought Index Sensitive to Global Warming: The Standardized Precipitation Evapotranspiration Index. *Journal of Climate,* 23**,** 1696-1718.

VON SCHUCKMANN, K., CHENG, L. J., PALMER, M. D., HANSEN, J., TASSONE, C., AICH, V., ADUSUMILLI, S., BELTRAMI, H., BOYER, T., CUESTA-VALERO, F. J., DESBRUYERES, D., DOMINGUES, C., GARCIA-GARCIA, A., GENTINE, P., GILSON, J., GORFER, M., HAIMBERGER, L., ISHII, M., JOHNSON, G. C., KILLICK, R., KING, B. A., KIRCHENGAST, G., KOLODZIEJCZYK, N., LYMAN, J., MARZEION, B., MAYER, M., MONIER, M., MONSELESAN, D. P., PURKEY, S., ROEMMICH, D., SCHWEIGER, A., SENEVIRATNE, S. I., SHEPHERD, A., SLATER, D. A., STEINER, A. K., STRANEO, F., TIMMERMANS, M. L. & WIJFFELS, S. E. 2020. Heat stored in the Earth system: where does the energy go? *Earth Syst. Sci. Data,* 12**,** 2013-2041.

VOROSMARTY, C. J., GREEN, P., SALISBURY, J. & LAMMERS, R. B. 2000. Global water resources: Vulnerability from climate change and population growth. *Science,* 289**,** 284-288.

VOS, T., ABAJOBIR, A. A., ABBAFATI, C., ABBAS, K. M., ABATE, K. H., ABD-ALLAH, F., ABDULLE, A. M., ABEBO, T. A., ABERA, S. F., ABOYANS, V., ABU-RADDAD, L. J., ACKERMAN, I. N., ADAMU, A. A., ADETOKUNBOH, O., AFARIDEH, M., AFSHIN, A., AGARWAL, S. K., AGGARWAL, R., AGRAWAL, A., AGRAWAL, S., KIADALIRI, A. A., AHMADIEH, H., AHMED, M. B., AICHOUR, A. N., AICHOUR, I., AICHOUR, M. T. E., AIYAR, S., AKINYEMI, R. O., AKSEER, N., AL LAMI, F. H., ALAHDAB, F., AL-ALY, Z., ALAM, K., ALAM, N., ALAM, T., ALASFOOR, D., ALENE, K. A., ALI, R., ALIZADEH-NAVAEI, R., ALKERWI, A., ALLA, F., ALLEBECK, P., ALLEN, C., AL-MASKARI, F., AL-RADDADI, R., ALSHARIF, U., ALSOWAIDI, S., ALTIRKAWI, K. A., AMARE, A. T., AMINI, E., AMMAR, W., AMOAKO, Y. A., ANDERSEN, H. H., ANTONIO, C. A. T., ANWARI, P., ARNLOV, J., ARTAMAN, A., ARYAL, K. K., ASAYESH, H., ASGEDOM, S. W., ASSADI, R., ATEY, T. M., ATNAFU, N. T., ATRE, S. R., AVILA-BURGOS, L., AVOKPAHO, E., AWASTHI, A., QUINTANILLA, B. P. A., SALEEM, H. O. B., BACHA, U., BADAWI, A., BALAKRISHNAN, K., BANERJEE, A., BANNICK, M. S., BARAC, A., BARBER, R. M., BARKER-COLLO, S. L., BARNIGHAUSEN, T., BARQUERA, S., BARREGARD, L., BARRERO, L. H., BASU, S., BATTISTA, B., BATTLE, K. E., BAUNE, B. T., BAZARGAN-HEJAZI, S., BEARDSLEY, J., BEDI, N., BEGHI, E., BEJOT, Y., BEKELE, B. B., BELL, M. L., BENNETT, D. A., BENSENOR, I. M., BENSON, J., BERHANE, A., BERHE, D. F., BERNABE, E., BETSU, B. D., BEURAN, M., et al. 2017. Global, regional, and national incidence, prevalence, and years lived with disability for 328 diseases and injuries for 195 countries, 1990-2016: a systematic analysis for the Global Burden of Disease Study 2016. *Lancet,* 390**,** 1211-1259.

WAN, Z. Y., WANG, J., WANG, K., HU, M. C. & WANG, X. T. 2021. Photocatalytic reduction of CO2 with H2O vapor into solar fuels over Ni modified porous In2O3 nanosheets. *Catal. Today,* 374**,** 44-52.

WANG, H. D., NAGHAVI, M., ALLEN, C., BARBER, R. M., BHUTTA, Z. A., CARTER, A., CASEY, D. C., CHARLSON, F. J., CHEN, A. Z., COATES, M. M., COGGESHALL, M., DANDONA, L., DICKER, D. J., ERSKINE, H. E., FERRARI, A. J., FITZMAURICE, C., FOREMAN, K., FOROUZANFAR, M. H., FRASER, M. S., PULLMAN, N., GETHING, P. W., GOLDBERG, E. M., GRAETZ, N., HAAGSMA, J. A., HAY, S. I., HUYNH, C., JOHNSON, C., KASSEBAUM, N. J., KINFU, Y., KULIKOFF, X. R., KUTZ, M., KYU, H. H., LARSON, H. J., LEUNG, J., LIANG, X. F., LIM, S. S., LIND, M., LOZANO, R., MARQUEZ, N., MENSAH, G. A., MIKESELL, J., MOKDAD, A. H., MOONEY, M. D., NGUYEN, G., NSOESIE, E., PIGOTT, D. M., PINHO, C., ROTH, G. A., SALOMON, J. A., SANDAR, L., SILPAKIT, N., SLIGAR, A., SORENSEN, R. J. D., STANAWAY, J., STEINER, C., TEEPLE, S., THOMAS, B. A., TROEGER, C., VANDERZANDEN, A., VOLLSET, S. E., WANGA, V., WHITEFORD, H. A., WOLOCK, T., ZOECKLER, L., ABATE, K. H., ABBAFATI, C., ABBAS, K. M., ABD-ALLAH, F., ABERA, S. F., ABREU, D. M. X., ABU-RADDAD, L. J., ABYU, G. Y., ACHOKI, T., ADELEKAN, A. L., ADEMI, Z., ADOU, A. K., ADSUAR, J. C., AFANVI, K. A., AFSHIN, A., AGARDH, E. E., AGARWAL, A., AGRAWAL, A., KIADALIRI, A. A., AJALA, O. N., AKANDA, A. S., AKINYEMI, R. O., AKINYEMIJU, T. F., AKSEER, N., AL LAMI, F. H., ALABED, S., AL-ALY, Z., ALAM, K., ALAM, N. K. M., ALASFOOR, D., ALDHAHRI, S. F., ALDRIDGE, R. W., ALEGRETTI, M. A., ALEMAN, A. V., ALEMU, Z. A., ALEXANDER, L. T., et al. 2016. Global, regional, and national life expectancy, all-cause mortality, and cause-specific mortality for 249 causes of death, 1980-2015: a systematic analysis for the Global Burden of Disease Study 2015. *Lancet,* 388**,** 1459-1544.

WATTS, N., AMANN, M., ARNELL, N., AYEB-KARLSSON, S., BEAGLEY, J., BELESOVA, K., BOYKOFF, M., BYASS, P., CAI, W. J., CAMPBELL-LENDRUM, D., CAPSTICK, S., CHAMBERS, J., COLEMAN, S., DALIN, C., DALY, M., DASANDI, N., DASGUPTA, S., DAVIES, M., DI NAPOLI, C., DOMINGUEZ-SALAS, P., DRUMMOND, P., DUBROW, R., EBI, K. L., ECKELMAN, M., EKINS, P., ESCOBAR, L. E., GEORGESON, L., GOLDER, S., GRACE, D., GRAHAM, H., HAGGAR, P., HAMILTON, I., HARTINGER, S., HESS, J., HSU, S. C., HUGHES, N., MIKHAYLOV, S. J., JIMENEZ, M. P., KELMAN, I., KENNARD, H., KIESEWETTER, G., KINNEY, P. L., KJELLSTROM, T., KNIVETON, D., LAMPARD, P., LEMKE, B., LIU, Y., LIU, Z., LOTT, M., LOWE, R., MARTINEZ-URTAZA, J., MASLIN, M., MCALLISTER, L., MCGUSHIN, A., MCMICHAEL, C., MILNER, J., MORADI-LAKEH, M., MORRISSEY, K., MUNZERT, S., MURRAY, K. A., NEVILLE, T., NILSSON, M., SEWE, M. O., ORESZCZYN, T., OTTO, M., OWFI, F., PEARMAN, O., PENCHEON, D., QUINN, R., RABBANIHA, M., ROBINSON, E., ROCKLOV, J., ROMANELLO, M., SEMENZA, J. C., SHERMAN, J., SHI, L. H., SPRINGMANN, M., TABATABAEI, M., TAYLOR, J., TRINANES, J., SHUMAKE-GUILLEMOT, J., VU, B., WILKINSON, P., WINNING, M., GONG, P., MONTGOMERY, H. & COSTELLO, A. 2021. The 2020 report of the Lancet Countdown on health and climate change: responding to converging crises. *Lancet,* 397**,** 129-170.

WAYCOTT, M., DUARTE, C. M., CARRUTHERS, T. J. B., ORTH, R. J., DENNISON, W. C., OLYARNIK, S., CALLADINE, A., FOURQUREAN, J. W., HECK, K. L., HUGHES, A. R., KENDRICK, G. A., KENWORTHY, W. J., SHORT, F. T. & WILLIAMS, S. L. 2009. Accelerating loss of seagrasses across the globe threatens coastal ecosystems. *Proceedings of the National Academy of Sciences,* 106**,** 12377-12381.

WEBSTER, P. J., HOLLAND, G. J., CURRY, J. A. & CHANG, H.-R. 2005. Changes in Tropical Cyclone Number, Duration, and Intensity in a Warming Environment. *Science,* 309**,** 1844-1846.

WEIGAND, H., BEERMANN, A. J., CIAMPOR, F., COSTA, F. O., CSABAI, Z., DUARTE, S., GEIGER, M. F., GRABOWSKI, M., RIMET, F., RULIK, B., STRAND, M., SZUCSICH, N., WEIGAND, A. M., WILLASSEN, E., WYLER, S. A., BOUCHEZ, A., BORJA, A., CIAMPOROVA-ZAT'OVICOVA, Z., FERREIRA, S., DIJKSTRA, K. D. B., EISENDLE, U., FREYHOF, J., GADAWSKI, P., GRAF, W., HAEGERBAEUMER, A., VAN DER HOORN, B. B., JAPOSHVILI, B., KERESZTES, L., KESKIN, E., LEESE, F., MACHER, J., MAMOS, T., PAZ, G., PESIC, V., PFANNKUCHEN, D. M., PFANNKUCHEN, M. A., PRICE, B. W., RINKEVICH, B., TEIXEIRA, M. A. L., VARBIRO, G. & EKREM, T. 2019. DNA barcode reference libraries for the monitoring of aquatic biota in Europe: Gap-analysis and recommendations for future work. *Science of the Total Environment,* 678**,** 499-524.

WEST, R., MICHIE, S., RUBIN, G. J. & AMLOT, R. 2020. Applying principles of behaviour change to reduce SARS-CoV-2 transmission. *Nat. Hum. Behav.,* 4**,** 451-459.

WESTERLING, A. L., HIDALGO, H. G., CAYAN, D. R. & SWETNAM, T. W. 2006. Warming and earlier spring increase western US forest wildfire activity. *Science,* 313**,** 940-943.

WILLIAMS, A. P., ALLEN, C. D., MACALADY, A. K., GRIFFIN, D., WOODHOUSE, C. A., MEKO, D. M., SWETNAM, T. W., RAUSCHER, S. A., SEAGER, R., GRISSINO-MAYER, H. D., DEAN, J. S., COOK, E. R., GANGODAGAMAGE, C., CAI, M. & MCDOWELL, N. G. 2013. Temperature as a potent driver of regional forest drought stress and tree mortality. *Nat. Clim. Chang.,* 3**,** 292-297.

WRIGHT, C. K. & WIMBERLY, M. C. 2013. Recent land use change in the Western Corn Belt threatens grasslands and wetlands. *Proceedings of the National Academy of Sciences of the United States of America,* 110**,** 4134-4139.

WRIGHT, I. J., REICH, P. B., WESTOBY, M., ACKERLY, D. D., BARUCH, Z., BONGERS, F., CAVENDER-BARES, J., CHAPIN, T., CORNELISSEN, J. H. C., DIEMER, M., FLEXAS, J., GARNIER, E., GROOM, P. K., GULIAS, J., HIKOSAKA, K., LAMONT, B. B., LEE, T., LEE, W., LUSK, C., MIDGLEY, J. J., NAVAS, M. L., NIINEMETS, U., OLEKSYN, J., OSADA, N., POORTER, H., POOT, P., PRIOR, L., PYANKOV, V. I., ROUMET, C., THOMAS, S. C., TJOELKER, M. G., VENEKLAAS, E. J. & VILLAR, R. 2004. The worldwide leaf economics spectrum. *Nature,* 428**,** 821-827.

XIA, B., XIAO, J. Z., DING, T. & ZHANG, K. J. 2021. Probabilistic sustainability design of structural concrete components under climate change. *Struct. Saf.,* 92**,** 13.

XIE, P. P. & ARKIN, P. A. 1997. Global precipitation: A 17-year monthly analysis based on gauge observations, satellite estimates, and numerical model outputs. *Bull. Amer. Meteorol. Soc.,* 78**,** 2539-2558.

ZACHOS, J., PAGANI, M., SLOAN, L., THOMAS, E. & BILLUPS, K. 2001. Trends, rhythms, and aberrations in global climate 65 Ma to present. *Science,* 292**,** 686-693.

ZELINKA, M. D., MYERS, T. A., MCCOY, D. T., PO-CHEDLEY, S., CALDWELL, P. M., CEPPI, P., KLEIN, S. A. & TAYLOR, K. E. 2020. Causes of Higher Climate Sensitivity in CMIP6 Models. *Geophysical Research Letters,* 47**,** 12.

ZHAO, M. S. & RUNNING, S. W. 2010. Drought-Induced Reduction in Global Terrestrial Net Primary Production from 2000 Through 2009. *Science,* 329**,** 940-943.
